# Polymorphic toxin systems: Comprehensive characterization of trafficking modes, processing, mechanisms of action, immunity and ecology using comparative genomics

**DOI:** 10.1186/1745-6150-7-18

**Published:** 2012-06-25

**Authors:** Dapeng Zhang, Robson F de Souza, Vivek Anantharaman, Lakshminarayan M Iyer, L Aravind

**Affiliations:** 1 National Center for Biotechnology Information, National Library of Medicine, National Institutes of Health, Bethesda, MD, 20894, USA; 2 Departamento de Microbiologia, Instituto de Ciências Biomédicas, Universidade de São Paulo, São Paulo, Brazil

## Abstract

**Background:**

Proteinaceous toxins are observed across all levels of inter-organismal and intra-genomic conflicts. These include recently discovered prokaryotic polymorphic toxin systems implicated in intra-specific conflicts. They are characterized by a remarkable diversity of C-terminal toxin domains generated by recombination with standalone toxin-coding cassettes. Prior analysis revealed a striking diversity of nuclease and deaminase domains among the toxin modules. We systematically investigated polymorphic toxin systems using comparative genomics, sequence and structure analysis.

****Results**:**

Polymorphic toxin systems are distributed across all major bacterial lineages and are delivered by at least eight distinct secretory systems. In addition to type-II, these include type-V, VI, VII (ESX), and the poorly characterized “*Photorhabdus* virulence cassettes (PVC)”, PrsW-dependent and MuF phage-capsid-like systems. We present evidence that trafficking of these toxins is often accompanied by autoproteolytic processing catalyzed by HINT, ZU5, PrsW, caspase-like, papain-like, and a novel metallopeptidase associated with the PVC system. We identified over 150 distinct toxin domains in these systems. These span an extraordinary catalytic spectrum to include 23 distinct clades of peptidases, numerous previously unrecognized versions of nucleases and deaminases, ADP-ribosyltransferases, ADP ribosyl cyclases, RelA/SpoT-like nucleotidyltransferases, glycosyltranferases and other enzymes predicted to modify lipids and carbohydrates, and a pore-forming toxin domain. Several of these toxin domains are shared with host-directed effectors of pathogenic bacteria. Over 90 families of immunity proteins might neutralize anywhere between a single to at least 27 distinct types of toxin domains. In some organisms multiple tandem immunity genes or immunity protein domains are organized into polyimmunity loci or polyimmunity proteins. Gene-neighborhood-analysis of polymorphic toxin systems predicts the presence of novel trafficking-related components, and also the organizational logic that allows toxin diversification through recombination. Domain architecture and protein-length analysis revealed that these toxins might be deployed as secreted factors, through directed injection, or via inter-cellular contact facilitated by filamentous structures formed by RHS/YD, filamentous hemagglutinin and other repeats. Phyletic pattern and life-style analysis indicate that polymorphic toxins and polyimmunity loci participate in cooperative behavior and facultative ‘cheating’ in several ecosystems such as the human oral cavity and soil. Multiple domains from these systems have also been repeatedly transferred to eukaryotes and their viruses, such as the nucleo-cytoplasmic large DNA viruses.

****Conclusions**:**

Along with a comprehensive inventory of toxins and immunity proteins, we present several testable predictions regarding active sites and catalytic mechanisms of toxins, their processing and trafficking and their role in intra-specific and inter-specific interactions between bacteria. These systems provide insights regarding the emergence of key systems at different points in eukaryotic evolution, such as ADP ribosylation, interaction of myosin VI with cargo proteins, mediation of apoptosis, hyphal heteroincompatibility, hedgehog signaling, arthropod toxins, cell-cell interaction molecules like teneurins and different signaling messengers.

****Reviewers**:**

This article was reviewed by AM, FE and IZ.

## Background

Production and deployment of “chemical armaments” is one of the most common strategies in inter-organismal conflict. Such molecules, namely toxins or antibiotics, are observed at practically every level of biological organization ranging from multicellular organisms like animals and plants, through bacteria, all the way down to intra-genomic selfish elements [1-4]. These molecules span an entire biochemical spectrum from diffusible small molecules (e.g. antibiotics) to some of the largest proteins in the biological world (secreted bacterial toxins)[5,6]. Beyond their natural roles, these molecules have considerable significance as biotechnological reagents, biodefense agents, therapeutic targets, and therapeutics against numerous disease-causing agents [1,2,4,6,7]. Traditional toxicology has now been joined by genomics and sequence analysis in uncovering the enormous biochemical diversity across life forms of such molecules and of the systems that synthesize and traffic them. This diversity is seen both in the structure and action of systems involved in synthesis of diffusible antibiotics and proteinaceous toxins [5,6]. It is becoming increasingly clear that proteinaceous toxins are a common feature of biological conflicts at every organizational level [7]: 1) In antagonistic interactions between different multicellular eukaryotes, such as the castor bean ricin, *Aspergillus* sarcin and various snake venom proteins [2,3,8,9]. 2) Action by multicellular organisms against their pathogens (e.g. anti-microbial peptide toxins and defensive RNases such as RNaseA and RNase L [10-13]). 3) Action of pathogenic and symbiotic bacteria directed against their hosts (e.g. the cholera toxin and the shiga toxin [4,14]). 4) Inter-specific conflict in bacteria [15]. 5) Conflict between bacterial sibling strains of the same species, namely contact dependent inhibition systems and related secreted toxins [16-19]. 6) Inter-genomic conflicts between cellular genomes and selfish replicons residing in the same cell (e.g. classical bacteriocins and plasmid addiction toxins [20]). 7) Intra-genomic conflicts between selfish elements and the host genome (restriction-modification systems [21] and genomic toxin-antitoxin systems [22-24]).

Studies in the past decade are pointing to certain unifying themes across the proteinaceous toxins deployed in each of these distinct types of biological conflict. The most prominent theme is the use of enzymatic toxins that disrupt the flow of biological information by targeting nucleic acids and proteins [7]. Thus, several toxin domains are nucleases targeting genomic DNA, tRNAs and rRNAs, nucleic acid base glycosylases, nucleic acid-modifying enzymes, peptidases that cleave key protein targets, and protein-modifying enzymes that alter the properties of proteins, such as components of the translation apparatus [4,6,7,17,18,25]. A secondary theme seen across toxins from phylogenetically diverse sources is the presence of domains that disrupt cellular integrity by forming pores in cellular membranes [26,27]. Genomic analysis has also revealed that the richest source of proteinaceous toxins is the bacterial superkingdom, wherein several systems involved in most of the levels of biological conflict enumerated above are encountered [4,6,17,18,21,22,25].

It is also becoming apparent that inter- and intra- specific and inter- and intra- genomic conflicts in prokaryotes have resulted in an intense arms race with respect to proteinaceous toxins. There is evidence for multiple episodes of escalation of the conflict in terms of the evolution of immunity proteins, followed by alterations in the toxins to evade the action of the immunity proteins [15,17,18,24,28]. Another major evolutionary theme seen in secreted proteinaceous toxins is the exploration of several alternative secretory mechanisms for their effective trafficking and delivery to potential targets. In particular, bacteria display at least eight distinct secretory mechanisms over and beyond the ancestral Sec (or Type II) system that is shared with the other branches of life (Table 1). Both the T2SS and alternative secretory mechanisms have been repeatedly coopted for trafficking toxins [15,17,18,29,30]. In addition to the T2SS, examples of other widely utilized secretory pathways that have been frequently coopted for trafficking of toxins include three distinct systems dependent on ATPase pumps: 1) ABC ATPase-dependent Type I system, which has been adapted for the delivery of the large RTX toxins [31]; 2) the FtsK-like ATPase-dependent type VII (ESX) system of Gram-positive bacteria, which has been recruited for delivering several toxins, including those frequently deployed in intraspecific conflict [17,32,33]; 3) the plasmid conjugation apparatus-derived type IV system [34], which is also dependent on FtsK-related ATPases [33]. On the other hand some of the other alternative secretory mechanisms appear to be primarily utilized in trafficking toxins rather than any other function: 1) The type III system based on the flagellar basal body-like apparatus [35]; 2) the two-partner or Type V system which resembles the porins [36,37]; 2) the type VI [38,39]; 3) *Photorhabdus* virulence cassette (PVC)-type secretory system [40,41]. Both T6SS and the PVC-SS utilize caudate bacteriophage tail-derived proteins as an “injection syringe” and distinct AAA + ATPases to recycle the injection apparatus in an ATP-dependent manner after a single use [39]; 4) TcdB/TcaC-like export pathway [42]; 4) the PrsW-like peptidase-dependent system export system [43]. Depending on the secretory pathway, toxins might either be directly injected into target cells (e.g. T6SS delivered toxins) or diffuse into the surrounding medium (e.g. certain T2SS or T7SS toxins) or be anchored on the surface of producing cells to be delivered upon contact with the target cell (e.g. T5SS and certain T2SS, T6SS and T7SS delivered toxins). Additionally, these prokaryotic toxins might also display further adaptations that allow their processing subsequent to their secretion – these include the presence of “pre-toxin domains” that might be sites for proteolytic processing or in-built peptidase domains that cleave off the toxin domain to facilitate its delivery into the target cell [17,20] (Table 1).

**Table 1:** Features of secretion pathways by which polymorphic toxins are exported

**Secretion pathway**	**Signature N-terminal leader domains or pre-toxin-domains**	**Signature genes in neighborhood**	**Processing proteases/repeats in toxin proteins**	**Phyletic patterns**	**Additional Notes**
T2SS/Sec-dependent system	Signal peptide	-	*Proteases:* Caspase, HINT, MCF1-SHE, subtilisin^3^, ZU5^4^	In all bacteria	Default pathway for protein export. Might contain MAFB-N (DUF1020), MicroscillaN, APD1, APD2, Inactive transglutaminase
			*Repeats:* ALF, ankyrins, β-propeller, RHS, Sel1^1^, TPR^1^, Tail-fiber^2^		
T5SS	N-terminal TpsA-like secretion domain (TPSASD)	FhaB/CdiB coding for porin-like protein	*Proteases:* HINT *Repeats:* FilH	α,β,γ,δ,ϵ-proteobacteria, acidobacteria, bacteroidetes/chlorobi, firmicutes^5^, fusobacteria	The TPSASD domain binds the outer-membrane FhaB/CdiB during the export of the toxin domain
	*Pre-toxin domains:* DUF637(PT637),DUF637-N, PT-VENN				
T6SS	VgrG domain, PAAR domain, Hcp1	ClpV-like AAA + Atpase, MOG1/PspB-like, VgrG, Hcp1, Phage tail/base-plate related proteins	*Repeats:* RHS	All proteobacteria, acidobacteria, bacteroidetes/chlorobi, firmicutes	Complete T6SS delivered toxins are often typified by a N-terminal PAAR domain
*Photorhabdus* virulence cassette pathway (PVC)	PVC-Metallopeptidase	CDC48-like AAA + ATPase, VgrG, Phage tail/base-plate related proteins	*Proteases:* Metallopeptidase, Subtilisin, Caspase, MCF1-SHE	Euryarchaeota, α,β,γ,δ,ϵ-proteobacteria, acidobacteria, actinobacteria, bacteroidetes, chlorobi, chloroflexi, cyanobacteria, deinococci, firmicutes, nitrospirae, spirochaetes	
			*Repeats:* RHS, tail fiber		
T7SS/ESX/ESAT-6 secretion system	WxG, LxG, LDxD domains	YueA-like FtsK/HerA ATPase, EsaC	*Proteases:* HINT, Caspase, MCF1-SHE *Repeats:* RHS, Tail-fiber	Firmicutes, actinobacteria, chloroflexi, other bacterial lineages^6^	Toxins exported by these systems may or may not possess repeat domains
TcdB/TcaC	A signal peptide followed by a SpvB domain coupled to a C-terminal integrin-like β-propeller domain	TcdB	*Repeats****:*** Integrin-like beta propeller, RHS, tail-fiber	Euryarchaeota, α,β,γ,δ-proteobacteria, actinobacteria, bacteroidetes	
				Chloroflexi, fibrobacteres, firmicutes, lentisphaerae, spirochaetes	
			*Proteases*: HINT, Caspase, ZU5		
PrsW	PrsW-peptidase domain		*Repeats:* RHS	Euryarchaeota, α,β,γ,δ-proteobacteria, actinobacteria Bacteroidetes, chloroflexi, cyanobacteria,deinococci, dictyoglomi, firmicutes, fusobacteria, gemmatimonadetes, spirochaetes, verrucomicrobia	PrsW is a transmembrane peptidase with several transmembrane helices
			*Proteases*: PrsW		
Phage DNA packaging system	MuF	MuF, large and small subunits of terminase	*Proteases:* Papain-like	Euryarchaeota, acidobacteria, α,γ,δ-proteobacteria, actinobacteria, bacteroidetes, chlorobi, firmicutes, fusobacteria, spirochaetes, caudovirales	The toxin is predicted to be packaged into the phage head as in phage transduction systems

1: Note only fused to toxins exported by the SEC-dependent pathway in *Amoebophilus asiaticus*; 2. Note only fused to toxins exported by the SEC-dependent pathway in *Microscilla marina*; 3: Note only fused to toxins exported by the SEC-dependent pathway in *Acetivibrio cellulolyticus*; 4: Note only fused to toxins exported by the SEC-dependent pathway in *Caldicellulosiruptor* species; 5: Note in firmicutes, the export pathway is only present in *Veillonella* and *Selenomonas* species, also referred to as the Negativicutes species; 6: Certain bacterial lineages within the β,ϵ,γ-proteobacteria, planctomycetes, verrucomicrobia, cyanobacteria and bacteroidetes have solo WXG domains that have a distinct YueA-like ATPase with 3 HerA/FtsK domains of which only the middle one is active. These appear to be mobile versions of T7SS.

**Figure 1:**
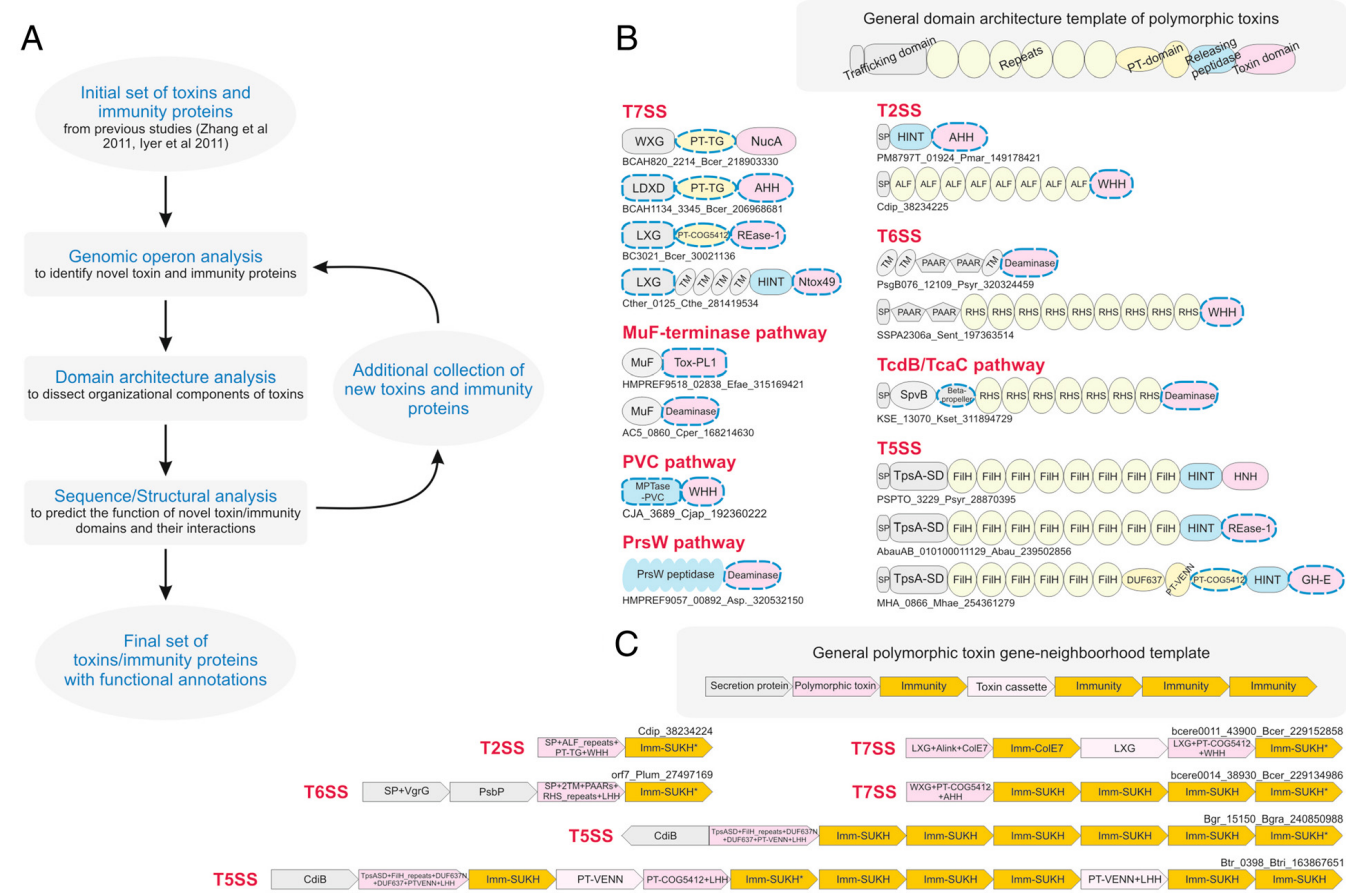
**(A) Workflow for identification and analysis of toxin and immunity domains in bacterial polymorphic toxin systems.** (**B**) General domain architecture template for polymorphic toxins along with representative architectures seen in different secretory systems. Trafficking domains are colored grey, repeats light green, pre-toxin domains (PT-domain) yellow, releasing peptidases blue, and toxin domains pink. Newly identified domains are encircled in dashed lines in all figures in this paper. Proteins are not drawn to scale. Note, only repeats automatically detected by profiles are shown in all figures; the proteins usually have much longer repeat units than shown due to repeats being below the detection threshold. Toxins are grouped based on their secretion pathways that are defined by their canonical trafficking domains (Table 1). Proteins are denoted by their gene name, species abbreviations and GI (Genbank Index) numbers separated by underscores. (**C**) General gene-neighborhoods template for polymorphic toxin operons. Individual genes are represented as arrows pointing from the 5′ to the 3′-end of the coding frame. Genes are labeled by their domain architectures. The gene neighborhood is labeled by the gene name, species abbreviation and GI number of the SUKH gene marked with an asterisk. Toxins are colored pink, immunity proteins orange, and other trafficking related proteins grey. For species abbreviations refer to supplementary material.

The selective pressures related to the above-described adaptations for trafficking, processing and delivery appear to have been instrumental in shaping the domain architectures of plasmid-encoded bacteriocins and prokaryotic toxins deployed in inter- and intra-specific conflicts [17,20]. Consequently, most toxin proteins have N-terminal domains involved in secretion and/or cell surface anchorage, central domains involved in adhesion or presentation to target cells and C-terminal domains that bear the actual toxin activity (Figure 1, Table 1). These might be occasionally combined with further processing-peptidase or pre-toxin domains [17,18,20]. These stereotypic architectural features strongly distinguish such toxins from those involved in intra-genomic conflicts, such as those from classical toxin-antitoxin systems and restriction-modification systems, even though certain domains with toxin activity might be common across these different systems [17,22,28]. Hence, domain architectural analysis considerably aids in the detection of new toxins involved in inter-organismal conflicts and the delineation of specific domains associated with each of the above-listed trafficking related roles. This has led to an exciting discovery in the past two years, namely the identification and characterization of an extremely widespread system of secreted toxins, primarily involved in intra-specific conflict between related strains of prokaryotes [16-19]. These toxin systems are found in practically all major bacterial lineages and also a small number of archaea. Toxin proteins of these systems are as a rule multi-domain and display a bewildering diversity in terms of domains possessing toxin activity [17,18]. An important feature of these proteins is the tendency to vary their toxin domains through a process of recombination that might replace an existing toxin domain by a distinct one encoded by standalone cassettes, while retaining the rest of the protein’s architecture (i.e. parts related to trafficking and delivery) intact. As a consequence these toxins might be termed *polymorphic toxins* and encompass the so called contact dependent inhibition (CDI) systems that were recently described in proteobacteria [17,44,45]. Further, these systems typically possess a chromosomally linked immunity protein that helps in protecting cells against their own toxin. These systems might also display several more chromosomally linked or distantly located immunity proteins that could serve as a potential line of defense against toxins delivered by “non-self” strains. The presence of immunity proteins is a key feature that distinguishes the polymorphic toxins from conventional toxins whose primary targets are in distantly related organisms (hence, no “self” immunity is required). Thus, these polymorphic secreted toxins could play a central role in “self versus non-self” or kin recognition in bacteria and thereby have an important role in regulating intra-specific altruistic and cooperative behavior [17,18].

**Figure 2:**
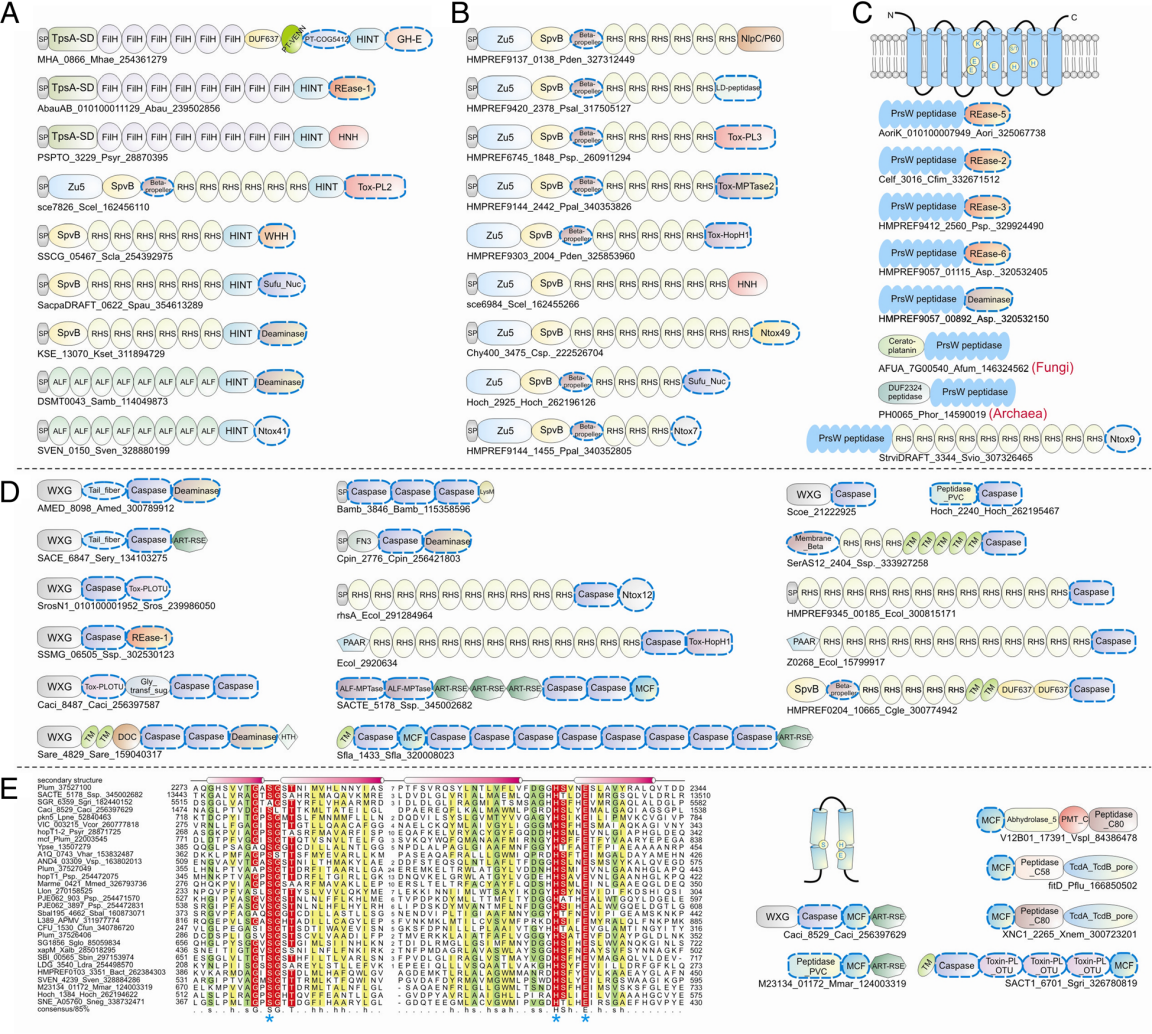
**Domain architectures of selected examples of polymorphic toxins containing distinct releasing peptidases: (A) HINT, (B) ZU5, (C) PrsW peptidase, (D) Caspase peptidase, (E) MCF1-SHE-like predicted peptidase.** The alignment of MCF1-SHE domain is shown with predicted catalytic residues marked with blue asterisks. For all alignments in this study, proteins are denoted by their gene name, species abbreviations and GI (Genbank Index) numbers separated by underscores. Secondary structure assignments are shown above the alignment, where the blue arrow represents the β-strand and the red cylinder the α-helix. Poorly conserved inserts are excluded in the alignment and replaced by the length of the inserts. Columns in the alignment are colored based on their amino acid conservation at consensus shown below the alignment. The coloring scheme and consensus abbreviations are as follows: h, hydrophobic (ACFILMVWY), l, aliphatic (LIV) and a, aromatic (FWY) residues shaded yellow; b, big residues (LIYERFQKMW), shaded gray; s, small residues (AGSVCDN) and u, tiny residues (GAS), shaded green; p, polar residues (STEDKRNQHC) shaded blue; and c, charged residues (DEHKR) shaded magenta. Absolutely conserved residues are shaded red.

 Our studies on the toxin domains of these polymorphic toxin systems have uncovered a remarkable array of nucleases and deaminases that are likely to target different cellular nucleic acids [17,18]. Our preliminary investigations also uncovered some other toxin domains in these systems with alternative modes of action, such as protein AMP/UMPylating enzymes, ADP-ribosyltransferases and peptidases. Interestingly, we observed that several of the toxin and processing peptidase domains from polymorphic secreted toxins are also present as toxin domains of conventional toxins deployed in inter-specific conflict, such as against eukaryotic hosts by pathogenic or symbiotic bacteria [46-54]. In a similar vein, we observed that both the polymorphic toxins deployed in intra-specific conflicts and toxins used in inter-specific conflict often rely on similar secretory mechanisms, such as the T5SS, T6SS and T7SS [17,18]. These observations suggested that both types of secreted toxins have been “constructed” in course of evolution from a common pool of domains and consequently possess similarities in their domain architectures. We also observed that several domains seen in secreted prokaryotic toxins and their immunity proteins have been transferred to eukaryotes and their viruses, and have contributed to the provenance of major regulatory molecules in the development of multicellular animals, RNA-editing, DNA-mutagenesis and virus-host interactions [17,18]. Thus, the evolutionary and functional significance of domains found in prokaryotic toxin systems extends beyond the mechanisms and dynamics of intra-organismal conflict.

Our previous studies on the polymorphic toxins focused on identifying and characterizing the diversity of toxin domains that operate on nucleic acids, in particular nucleases and deaminases, and characterizing some of the most prevalent immunity proteins, such as those with the SUKH and SuFu domains. We also reported a preliminary characterization of the major secretory systems involved in toxin trafficking and processing peptidases. Here, we build on our previous studies to systematically characterize novel domains in polymorphic toxin systems, with a particular focus on those involved in toxin activity, immunity and maturation of toxins. Consequently, we report herein a greatly expanded repertoire of toxin domains and immunity proteins directed against them. Thus, we also considerably extend their structural and mechanistic diversity to include a diverse array of peptidases, ADP ribosyltransferases, glycosyltransferases, kinases, membrane perforators and domains with several other activities. Even in terms of toxin acting on nucleic acids we report numerous previously unrecognized nucleases and deaminases. This expanded repertoire of toxin domains also helps to better understand the commonalities between the polymorphic toxin systems and the classical secreted toxins deployed against distantly related organisms. This comprehensive characterization also provides a handle to investigate the ecological significance of such secreted toxin systems in prokaryotes. Our analysis also uncovered novel features regarding the secretory systems that traffic these toxins. The detailed analysis of these toxin systems and their immunity proteins further pointed to several additional examples of domains from them being acquired by eukaryotes and their viruses. Thereby we greatly widen the contributions of components of these systems to the evolution of several eukaryotic regulatory systems. We present a comprehensive inventory of intra-specific polymorphic toxin systems and related components from toxin systems deployed in inter-specific conflicts. This database is likely to serve as an useful reference for future studies on this enormously significant group of proteins.

## Results and discussion 

### **Search strategy to identify new toxins and immunity proteins**

In order to identify novel polymorphic toxins we adopted a strategy of matching diagnostic domain-architecture and gene-neighborhood templates, similar to what we had done earlier to identify novel type II toxin-antitoxin systems [22]. In the case of polymorphic toxins the domain architecture template is defined by the presence of multi-domain proteins, wherein the C-terminal-most domain has toxin activity, while the N-terminal-most domains are associated with trafficking (Table 1, Figure 1). The central domains might be involved in adhesion, presentation or processing. One of the most common features of this central region is the presence of RHS (Recombination hot spot)/YD or filamentous hemagglutinin (FilH) repeats which form extended fibrous or filamentous structures that help in displaying the C-terminal toxin domain on the cell-surface [17,18,37,45,55,56]. With the above domain-architecture template (Figure 1), we identified an initial set of exemplars, which were used in sequence similarity searches to identify homologs that were similar over most of their length but differing in their C-terminal-most domains – a hallmark of polymorphic toxins (Figure 1B). This enabled us to precisely define the boundaries of the C-terminal toxin domains and use them as seeds in iterative sequence profile searches with the PSI-BLAST and JACKHMMER programs. These searches allowed us to recover both standalone toxin domain cassettes and examples where they are combined with other types of N-terminal trafficking, presentation and processing domains, distinct from those found in the starting queries. This process was used transitively to detect further toxin domains and full length toxins. As a result, we were able to not only capture other polymorphic toxins but also identify cases where these toxin domains might be used as the active domains of other secreted toxins that are deployed against more distantly related organisms (e.g. T3SS or T4SS delivered host-directed toxins). To further understand the sequence and structure affinities of toxin domains, we also used their multiple alignments in profile-profile comparisons with the HHpred program to recover distant homologs and determine their protein fold. Additionally, detailed domain-architecture analysis of the associated domains in the case of the full length toxins allowed us to delineate the domains involved in the other processes mentioned above.

In terms of gene-neighborhood templates (Figure 1), we exploited the fact that the polymorphic toxin genes are accompanied by several solo toxin cassettes and genes for immunity proteins and in some cases genes encoding trafficking components (e.g. T6SS or PVC-SS). Hence, we systematically extracted the genomic neighborhoods for all detected toxin-encoding genes from complete genome sequences or assembled CONTIGs and subjected them to gene-neighborhood analysis. Matches to the above template allowed us to distinguish the classical polymorphic toxins from related toxin systems that are deployed against more distantly related organisms. A combination of the gene-neighborhood analysis with the domain architecture analysis also allowed us to determine the trafficking mechanisms of full-length toxins in the majority of cases. Further, this genomic analysis also led to the recovery of potential immunity proteins associated with the polymorphic toxins. The identification of novel immunity proteins utilized the fact that the immunity protein gene/s are invariably adjacent to the toxin gene in an operon and typically encode a small single domain protein (Figure 1). We confirmed novel immunity proteins by initiating sequence searches with them and using the newly detected homologs in gene-neighborhood analysis to check if they showed any co-occurrence with toxin genes. The gene-neighborhood analysis of the newly identified immunity proteins also helped recover any loci that might have been missed in the initial toxin-centric analysis and also pointed to certain novel types of loci comprised primarily of multiple immunity genes (See below).

 As a result of the above searches, we were able to assemble a comprehensive inventory of toxins and immunity proteins, which we provide as a resource accompanying this article (Table 2, 3 and Additional File 1). For the sake of systematic nomenclature we adopted the following convention: 1) The toxin domains are labeled ‘Tox’ followed by the name of the superfamily they belong to. Thus, a toxin domain of the restriction endonuclease (REase) superfamily would be labeled Tox-REase. 2) The domain might be further distinguished by a numeral if there are multiple distinct toxin families within a given superfamily, e.g. Tox-REase-1, Tox-REase-2 and so on. 3) In the case of certain highly divergent families, each with their own structurally distinct features, such as those belonging to the HNH/EndoVII nuclease fold, each family of toxin domains might receive a separate label, e.g., Tox-HNH, Tox-AHH, Tox-LHH or Tox-NucA that identifies the specific family of nucleases. 4) Novel toxins that could not be unified with any previously known superfamily are labeled as ‘Ntox’ followed by a number, e.g. Ntox1, Ntox2 etc. (we identified a total of 50 such novel, monophyletic toxin groups in this study). 5) The immunity proteins were similarly named according to their superfamily. Thus, immunity proteins of the SUKH, SuFu and LRR superfamilies are respectively labeled as Imm-SUKH, Imm-SUFU or Imm-LRR. 6) Novel immunity proteins that could not be unified with any known superfamily were labeled as Imm followed by a number, e.g. Imm1, Imm2 etc. (we detected 73 such immunity proteins in this work).

**Table 2:** Phyletic distribution, export pathways, and contextually-associated domains and proteins of polymorphic toxin domains

**Toxin**^**1**^	**Fold; conserved residues or motifs**^**2**^**and additional notes**	**Phyletic spread**^**3**^	**Export pathway**^**4**^	**Immunity proteins**	***Repeats*/processing*Proteases***
**DNase toxins**					
Tox-NucA	HNH/EndoVII fold; GH, N, N, E	Actinobacteria, α,β,γ,δ-proteobacteria, bacteroidetes, chloroflexi, firmicutes, spirochaetes, verrucomicrobia	T2SS, T5SS, T6SS, T7SS (WXG,LXG,LDXD), PVC	Imm36, Imm-SUKH, Imm-NTF2	*Proteases:* PVC-Metallopeptidase, Caspase; *Repeats*: FilH, RHS, Tail-fiber
Tox-ColE7	HNH/EndoVII fold (PDB: 1zns);HH, H, H	Bacteroidetes, α,γ,δ,ϵ-proteobacteria, firmicutes	T2SS, T5SS, T6SS, T7SS (WXG,LXG), PyocinS	Imm-ColE7, Imm-SUKH	*Repeats*: FilH, RHS
Tox-HNH (including Tox-HNH-CIDE)	HNH/EndoVII fold; A DHxxE characterizes the Tox-HNH-CIDE clade.	Acidobacteria, actinobacteria, bacteroidetes, chlorobi, firmicutes, proteobacteria, ***Eukaryotes*:metazoa**	T2SS, T5SS, T7SS (WXG,LXG, LDXD), PVC, TcdB/TcaC	Imm-SUKH, Imm-SuFu, Imm14, Imm18, Imm24, Imm33,	*Proteases:* PVC-Metallopeptidase, HINT, Tox-PLOTU, ZU5; *Repeats*: FilH, RHS
Tox-AHH	HNH/EndoVII fold; [AG]HH, N, H, H, Y motif and residues	Actinobacteria, α,β,γ,δ,ϵ-proteobacteria, bacteroidetes, cyanobacteria, firmicutes, fusobacteria, lentisphaerae, planctomycetes, spirochaetes, verrucomicrobia, ***eukaryotes:*hexapoda,*Viruses:*Ostreococcus lucimarinus virus,*Bathycoccus*sp. RCC1105 virus**	T2SS, T5SS, T6SS, T7SS (LXG, WXG, LDxD), TcdB/TcaC	Imm-PA2201, Imm-ank, Imm11, Imm20, Imm23, Imm24, Imm43	*Proteases:* HINT; *Repeats*: RHS, FilH
Tox-DHNNK	HNH/EndoVII fold; DH, N, N, N, K motif and residues	Acidobacteria, actinobacteria α,β,γ,δ,ϵ-proteobacteria, firmicutes, fusobacteria, planctomycetes, spirochaetes, *archaea:* euryarchaeota, ***eukaryotes:*fungi(ascomycota, basidiomycota)**	T2SS, T6SS, T7SS (LXG, LDXD,WXG), PVC	Imm-SUKH, Imm-SuFu, Imm33	*Proteases:* PVC-Metallopeptidase, HINT
Tox-EHHH	HNH/EndoVII fold; [ED]H, H, H	Actinobacteria, bacteroidetes, β,γ,δ-proteobacteria, firmicutes	T2SS, T5SS T6SS, T7SS (WXG, LxG), TcdB/TcaC	Imm8, Imm50	*Repeats*: FilH, RHS
Tox-GH-E	HNH/EndoVII fold; GH, E, N, E motif and residues	Actinobacteria, bacteroidetes, β,γ,δ,ϵ-proteobacteria, chloroflexi, firmicutes, planctomycete, spirochaetes, *archaea:* euryarchaeota	T2SS (MafBN), T5SS, T6SS, T7SS (WXG, LxG, LDXD), PVC	Imm-SuFu, Imm-ank	*Proteases:* HINT, PVC-Metallopeptidase; *Repeats*: RHS, FilH, Tail Fiber
Tox-GHH	HNH/EndoVII fold; WxxE, W, G[HQ]H, NIxF, [DE]H; Eukaryotic versions lack the conserved histidines and a C-terminal helix	Acidobacteria, bacteroidetes, firmicutes, γ-proteobacteria, planctomycete, ***eukaryotes:*metazoa**	T2SS, T6SS, T7SS (LXG), TcdB/TcaC	Imm-SUKH	*Repeats*: RHS
Tox-GHH2	HNH/EndoVII fold; s[AGP]HH, HxxxH	β,γ-proteobacteria, bacteroidetes, firmicutes	T2SS, T6SS	-	*Repeats*: RHS
Tox-HHH	HNH/EndoVII fold; N, s[GD]xxR, HHH, H	Actinobacteria, bacteroidetes, γ-proteobacteria, firmicutes	T2SS, T5SS,T6SS, T7SS (LXG,LDXD), PVC	Imm-SUKH	*Proteases:* PVC-Metallopeptidase; *Repeats*: FilH, RHS
Tox-LHH	HNH/EndoVII fold; N, LHH, E, H, H, W	Actinobacteria, α,β,γ,δ,ϵ-proteobacteria, bacteroidetes, firmicutes, fusobacteria, planctomycetes	T2SS, T5SS, T6SS, T7SS (WXG,LXG), PVC	Imm-SUKH	*Proteases:* PVC-Metallopeptidase, HINT; *Repeats*: FilH, RHS, Tail-fiber
Tox-SHH	HNH/EndoVII fold; [SG]HH, H motif and residue	Actinobacteria, α,β,γ,δ-proteobacteria, bacteroidetes, cyanobacteria, firmicutes, planctomycetes, ***eukaryotes:*crustacea,*viruses*: caudovirales**	T2SS, T5SS, T6SS, T7SS (LDXD, LXG, WXG)	Imm-SUKH, Imm11, Imm24, Imm30, Imm55	*Proteases:* HINT *Repeats*: FilH, RHS, ALF
NGO1392-like (Also known as Tox-SuFu-Nuc)	HNH/EndoVII fold; CxxC, DH, CXXC, Q	Actinobacteria, α,β,γ,δ-proteobacteria, chlorobi, chloroflexi, cyanobacteria, firmicutes, spirochaetes, ***eukaryotes:*alveolata(apicomplexa), choanoflagellida, metazoa, stramenopiles, viridiplantae,*Viruses:*several Mycobacteriophages, caudovirales**	T2SS (MafBN), T5SS, TcdB/TcaC, PVC	Imm-SuFu, Imm13, Imm21, Imm33, Imm38	*Proteases:* HINT, PVC-Metallopeptidase, ZU5; *Repeats*: FilH, RHS, Tail fiber
Tox-WHH	HNH/EndoVII fold; WHH, L, H, HxG	Actinobacteria, α,β,γ,δ,ϵ-proteobacteria, bacteroidetes, chloroflexi, firmicutes, fusobacteria, planctomycete, synergistetes	T2SS, T5SS, T6SS, T7SS (WXG, LXG, LDXD), PVC, TcdB/TcaC	Imm-SUKH, Imm28, Imm37	*Proteases:* HINT, PVC-Metallopeptidase; *Repeats*: RHS, ALF, FilH
Tox-REase-1	Restriction endonuclease fold; E, D, ExK, Q	Actinobacteria, bacteroidetes, β,γ,ϵ-proteobacteria, cyanobacteria, fusobacteria, firmicutes, gemmatimonadetes, planctomycetes, ***eukaryotes:*alveolata, heterolobosea**	T2SS,T5SS, T6SS, T7S (WXG,LXG), TcdB/TcaC	Imm-PA2201, Imm49	*Proteases:* HINT, Caspase, ZU5; *Repeats*: FilH, RHS, Tail-fiber
Tox-REase-2	Restriction endonuclease fold; E, DG, [DE]xK, T, W	Actinobacteria	T2SS, T7SS (WXG), PrsW	-	*Proteases:* PrsW-peptidase
Tox-REase-3	Restriction endonuclease fold; [KR]ExD, K, ExQxK	β,γ-proteobacteria, firmicutes	T2SS (MafBN), T6SS, T7SS (WXG), PrsW	Imm-SUKH, Imm7	*Proteases:* PrsW-peptidase; *Repeats*: RHS
Tox-REase-4	Restriction endonuclease fold; D, ExK	Actinobacteria, α,β,γ,δ-proteobacteria, bacteroidetes, cyanobacteria, firmicutes, planctomycetes, spirochaetes, ***eukaryotes:*stramenopiles**	T2SS, T5SS, T6SS, T7SS (WXG,LDXD), PrsW	Imm-SUKH, Imm22, Imm54	*Proteases:* PrsW-peptidase; HINT; *Repeats*: FilH, RHS, Tail fiber
Tox-REase-5	Restriction endonuclease fold; Y, FDG, EAK, Y, Q,W	Actinobacteria, α,β,γ,δ-proteobacteria, firmicutes, fusobacteria, ***Viruses:*caudovirales**	T2SS, T5SS, T6SS, PrsW	Imm52	*Proteases:* PrsW-peptidase; *Repeats*: FilH, RHS
Tox-REase-6	Restriction endonuclease fold; E, D, ExK, Q, Y	Actinobacteria, α,β,γ-proteobacteria, bacteroidetes, cyanobacteria, firmicutes, ***eukaryotes:*heterolobosea**	T2SS, T5SS, T6SS, T7SS (WXG), PrsW	Imm49	*Proteases:* PrsW-peptidase; *Repeats*: RHS, Tail fiber
Tox-REase-7	Restriction endonuclease fold; GxxxE, IxD, ExK, Q	Actinobacteria, α,γ,ϵ-proteobacteria, bacteroidetes, cyanobacteria, firmicutes, planctomycetes, verrucomicrobia	T2SS, T5SS, T6SS, T7SS (WXG)	ImmHEAT, Imm23, Imm54	*Proteases:* HINT; *Repeats*: FilH, RHS, Tail-fiber
Tox-REase-8	Restriction endonuclease fold; GxxxQ, DD, QxK	Actinobacteria, α,β,γ,δ-proteobacteria, bacteroidetes, chlorobi, chloroflexi, firmicutes, spirochaetes, verrucomicrobia, ***eukaryotes:*metazoa(crustacea, hexapoda,placozoa)**	T2SS (APD1)	-	*Repeats*: Ankyrin repeats, TPR repeats, RHS
Tox-Rease-9	Restriction endonuclease fold; GxxxH, E, D, ELKP, YxxE	Actinobacteria, γ-proteobacteria, bacteroidetes, chlamydiae, firmicutes	T2SS, T7SS (LxG)	Imm54	*Proteases:* HINT; *Repeats*: RHS
Tox-Rease-10	Restriction endonuclease fold; E, Q, [DE], ExKNY, R, DxRG	β,γ,ϵ-proteobacteria, firmicutes, fusobacteria, spirochaetes	T2SS, T5SS, T7SS (WXG, LXG),	Imm54, Imm70	*Repeats*: FilH
Tox-URI1	URI nuclease fold; Y, YxG, R, [RK]xxE, N	Actinobacteria, α,β,γ,δ-proteobacteria, bacteroidetes, chlamydiae, chloroflexi, firmicutes, lentisphaerae, nitrospirae, verrucomicrobia, *archaea:* euryarchaeota, **v*iruses:*Ostreococcus lucimarinus virus*, eukaryotes:*fungi**	T2SS, T5SS, T6SS, TcdB/TcaC	Imm14, Imm26, Imm44, Imm51	*Proteases:* HINT; *Repeats*: RHS, FilH, Tail fiber
Tox-URI2	URI nuclease fold; Y, KxG, [EQ]	Actinobacteria, α,β,γ-proteobacteria, bacteroidetes, firmicutes	T2SS, T6SS	Imm9, Imm39, Imm12, Imm44	*Proteases:* HINT; *Repeats*: RHS, Tail fiber
**RNase toxins of known fold**					
Tox-Barnase	Barnase-EndoU-ColicinE5/D-RelE like nuclease (BECR) fold (α + β); H, H, [ST], FP, [STD]	Actinobacteria, bacteroidetes, β,γ,δ,ϵ-proteobacteria, chlamydiae, chloroflexi, cyanobacteria, deinococci, fibrobacteres, firmicutes, fusobacteria, nitrospirae, planctomycetes *archaea:* euryarchaeota	T2SS, T6SS, T7SS (WXG), TcdB/TcaC, MuF, PVC	Imm-Barstar	*Proteases:* PVC-Metallopeptidase; *Repeats*: RHS
Tox-Colicin D	BECR fold (α + β); (PDB: 1v74); [KH]K, Hxx[ED], [ST], [TS]xxK; Of the conserved residues in ColicinD (PDB: 1v74), K607, K608, H611, D614, and S677 are essential for activity	β,γ,δ-proteobacteria, chloroflexi, firmicutes, spirochaetes, *archaea:* euryarchaeota, ***eukaryotes:*fungi (ascomycota)**	T2SS, T5SS, Cloacin, TcdB/TcaC, PVC, MuF	ImmD, Imm64; ImmD is the major immunity protein share with plasmid borne colicin systems	*Proteases:* PVC-Metallopeptidase; *Repeats*: RHS, FilH
Tox-ColicinC/E5 tRNase	BECR fold (α + β, PDB: 2dfx); K, W, Y, Y, Q, [RK], W; Of the conserved residues in Colicin E5 (PDB: 2dfx), Y81 and S95 are predicted to be involved in catalysis	β,γ-proteobacteria, firmicutes, Plasmid ColE5-099	T2SS, T5SS, T7SS (LXG), Cloacin/PyocinS, TcdB/TcaC	ImmE5	*Repeats*: RHS, FilH
Tox-EndoU (including XendoU)	BECR fold (α + β, PDB: 2c1w); H, H, [SNT],[SNT]; This structural core contains two BECR fold units, where the N-terminal unit has lost strand-4, while the helix in the C-terminal unit has flipped to the opposite end. In 2c1w, H162 and T278 form one pair of catalytic residues and H178 and S229 form the other. Some members use a Mn^2+^ probably as a transition state stabilizer	Actinobacteria, α,β,γ-proteobacteria, bacteroidetes, chlamydiae, cyanobacteria, fibrobacteres, firmicutes, fusobacteria, tenericutes, ***eukaryotes:*hemichordata, viridiplantae, stramenopiles, metazoa**	T2SS (MafBN), T5SS, T6SS, T7SS (WXG,LXG)	Imm-SUKH, Imm-SuFu, Imm28	*Proteases:* HINT; *Repeats*: FilH, RHS
Tox-RelE	BECR fold (α + β); [KR], R; The active site residues in the classical RelE (PDB: 3kha) correspond to residues R61 and R81	Actinobacteria, α, γ, -proteobacteria, bacteroidetes, cyanobacteria, firmicutes, fusobacteria	T2SS	Imm54	*Proteases:* HINT; *Repeats*: RHS
Ntox7	Predicted BECR fold (α + β); DGx + xhR, N motif	Actinobacteria, bacteroidetes, β,γ, δ- proteobacteria, chlamydiae, chloroflexi, firmicutes	T2SS (MafBN), T2SS (APD1), T5SS, T7SS, TcdB/TcaC	Imm8, Imm31, Imm32, Imm-NMB0513, Imm-SuFu; Imm8 is the predominant immunity protein across a wide phyletic range	*Proteases****:*** HINT, ZU5*; Repeats***:** FilH, RHS
Ntox19	Predicted BECR fold (α + β); D,H,DxxxR,E,HxxF; Also found in mimivirus, where it is fused to ankyrin repeats,	β,γ,δ- proteobacteria, firmicutes, fusobacteria, bacteroidetes, ***Viruses:*Acanthamoeba polyphaga mimivirus**	T2SS (MafBN), T5SS, T7SS (LxG and WxG), TcdB/TcaC	Imm38, Imm40. These associations are seen across many different bacterial lineages	*Repeats***:** FilH, RHS
Ntox21; Also referred to as the *E. clocae* CdiAC; Shown to be a tRNAse	Predicted BECR fold (α + β); K, [DS]xDxxxH, K, RxG[ST], RxxD	Actinobacteria, α,β,γ-proteobacteria bacteroidetes, firmicutes	T2SS (MafBN), T5SS, T4SS, T7SS	Imm-Barstar, Imm41	*Proteases****:*** HINT; *Repeats***:** RHS, FilH
Ntox35	Predicted BECR fold (α + β); H, KH	Actinobacteria, bacteroidetes, β-proteobacteria, chlamydiae, chloroflexi, firmicutes, planctomycetes	T2SS (MafBN)	-	*Repeats*: RHS
Ntox36	Predicted BECR fold (α + β); N, [RY], [DE]	Acidobacteria, actinobacteria, β,γ-proteobacteria, cyanobacteria, elusimicrobia, firmicutes	T2SS, T5SS	-	*Proteases:* HINT; *Repeats*: RHS, FilH
Ntox41	Predicted BECR fold (α + β); [RK]H, [KR], [ST]xxP	Actinobacteria, α,β,γ-proteobacteria, bacteroidetes, firmicutes, planctomycetes	T2SS, T5SS, T7SS (WXG,LXG)	-	*Proteases:* HINT; *Repeats*: RHS, FilH, ALF
Ntox47	Predicted BECR fold (α + β); D, [HRK], RT, E, D, PH, H, [DE], R	β,γ-proteobacteria, firmicutes	T2SS, T6SS, T7SS (LXG,WXG)	-	*Proteases:* HINT; *Repeats*: RHS
Ntox48	Predicted BECR fold (α + β); R, [RK], Q, Q	Acidobacteria, actinobacteria, α,β,γ,δ-proteobacteria, bacteroidetes, cyanobacteria, firmicutes, fusobacteria, planctomycetes	T2SS, T5SS, T6SS T7SS (WXG,LXG),	Imm60, Imm62, Imm66, Imm71	*Proteases:* HINT; *Repeats*: RHS, FilH
Ntox49	Predicted BECR fold (α + β); H, [KR]	Actinobacteria, α,β,γ,δ-proteobacteria, bacteroidetes, chlamydiae, chloroflexi, cyanobacteria, firmicutes, thermotogae, *archaea:* euryarchaeota, ***eukaryotes:*stramenopiles, viridiplantae,*viruses:*caudovirales**	T2SS (MafBN), T5SS, T7SS (WXG,LXG), MuF, PVC	Imm22	*Proteases:* PVC-Metallopeptidase, HINT, ZU5; *Repeats*: RHS
Ntox50	Predicted BECR fold (α + β); H, S, K, T, H, K, HxVP	Actinobacteria, β,γ,δ-proteobacteria, chlamydiae, firmicutes, fusobacteria, ***viruses:*caudovirales**	T2SS (MafBN), T6SS, T7SS (WXG,LXG), MuF	-	*Proteases:* HINT
**Predicted metal-independent RNase toxins**					
Tox-CdiAC	All-β; N, [DSN],E	β,γ,δ-proteobacteria	T2SS, T5SS, T6SS, TcdB/TcaC	Imm-CdiI, Imm5 + Imm36. Imm-CdiI is the most prominent immunity protein to this toxin	*Repeats*: RHS, FilH
Tox-ColE3	All-β; ColE3 cytotoxic ribonuclease fold, R, Dxx + [HK], E, H	Actinobacteria, α,β,γ-proteobacteria, bacteroidetes, cyanobacteria, firmicutes, fusobacteria	T2SS (MafBN), T5SS, T7SS (WXG,LXG)	Imm-Cloacin, Imm45	*Proteases:* HINT; *Repeats*: RHS, FilH
Tox-RES; PF08808 in Pfam. Also found in toxin-antitoxin systems (see text);	α + β; R, R, E, S	Acidobacteria, actinobacteria, α,β,γ,δ,ϵ-proteobacteria, bacteroidetes, chlorobi, chloroflexi, cyanobacteria, deinococci, firmicutes, nitrospirae, spirochaetes, synergistetes, verrucomicrobia, ***Viruses:*caudovirales**	T2SS, T5SS, T6SS	Imm51, Antitoxin-DUF2384(in AT system)	*Repeats*: RHS, FilH
Ntox2	α + β + α-helical C-terminus; GEsH motif and conserved E, RE, H and K; Multiple copies in the same gene neighborhood in *Microscilla*	*Microscilla marina* (Bacteroidetes)	PVC	-	*Proteases:* PVC-Metallopeptidase
Ntox4	α + β; Several charged residues	*Nitrosococcus, Frankia*	PVC	-	*Proteases****:*** PVC-Metallopeptidase
Ntox5	α + β; Several charged residues	*Streptomyces, Nitrobacter*	PVC	-	*Proteases****:*** PVC-Metallopeptidase
Ntox9	Mostly β; RxY, E, WxE and H; Catalytic mechanism likely to be similar to that of Colicin-E3	Actinobacteria, α,β,γ-proteobacteria bacteroidetes, chlamydiae, fusobacteria	T2SS (MafBN), T5SS, T6SS	-	*Proteases:* PrsW peptidase; *Repeats*: RHS
Ntox12	All-β; D, D, H	Actinobacteria, chlamydiae, firmicutes, α,β,γ- proteobacteria	T2SS, T5SS T6SS, T7SS (WxG and LxG), TcdB/TcaC	Imm32; Note immunity protein also present in intracellular parasite *Odyssella*	*Proteases:* OUT; *Repeats*: RHS, FilH
Ntox13	β/α, KxxxxxxE motif	Firmicutes, β-proteobacteria	T2SS	Imm59	*Repeats*: RHS
					*Proteases:* Transglutaminase
Ntox15	Mostly α, HxxD motif	Actinobacteria, firmicutes, α,β,γ- proteobacteria	T2SS, T6SS, T7SS (LDxD and LxG), PVC	Imm-SUKH	*Proteases:* PVC-Metallopeptidase, HINT
Ntox16	α-helical domain; R, [DNE]xxH; part of polytoxin in *Xanthomonas fuscans*	Cyanobacteria, β,γ, δ proteobacteria, verrucomicrobia	T2SS, T6SS, PVC	-	*Proteases:* PVC-Metallopeptidase; *Repeats*: RHS
Ntox17	Mostly β; ExD, H, several charged residues	α,β,γ proteobacteria, firmicutes	T2SS (MafB), TcdB/TcaC, T7SS	Imm31; association widespread several lineages	*Repeats*: RHS
Ntox20	Mostly β; conserved R	Acidobacteria, α,β,γ,ϵ-proteobacteria	T2SS (MafBN), T5SS	Imm-NMB0513, Imm-SUKH Imm28	*Repeats*: FilH
Ntox23	All-β;	Bacteroidetes	T2SS, TcdB/TcaC	-	*Repeats*: RHS
	ND, DxxR, H				
Ntox24	All-β; Y, H, H; Also found in Toxin-Antitoxin systems (see text)	Actinobacteria, α,β,γ-proteobacteria, chlamydiae, chloroflexi, firmicutes, fusobacteria	T2SS, T5SS T7SS (WXG,LXG), MuF	Imm50, Imm53	*Proteases:* HINT; *Repeats*: RHS, FilH
Ntox25	Mostly β; FGPY motif	α,γ-proteobacteria, bacteroidetes	T2SS, T5SS	-	*Repeats*: FilH
Ntox27	α + β; D, E, RxW	Actinobacteria, bacteroidetes, fusobacteria	T2SS, T7SS (WXG)	-	*Proteases:* HINT; *Repeats*: ALF, RHS
Ntox28	All-α; D,K[DE], [DN]HxxE, E	Actinobacteria, α,γ-proteobacteria, firmicutes	T2SS, T5SS T7SS (WXG)	-	*Repeats*: FilH
Ntox31	α + β; K, E, E	Actinobacteria, α,γ-proteobacteria, bacteroidetes, firmicutes, ***eukaryotes:*ciliophora**	T2SS, T5SS, T6SS, T7SS (WXG, LXG)	Imm62	*Repeats*: RHS, FilH
Ntox32	All-α; H, [KR], [ED], [DE]	Bacteroidetes, α,γ-proteobacteria, firmicutes, ***eukaryotes:*insects**	T2SS	-	*Proteases:* Peptidase S8 (Subtilisin family); *Repeats*: RHS
Ntox34	All-α; GNxxD, K, C, C, K, WxCxH and other charged residues	γ,δ,ϵ-proteobacteria, firmicutes	T2SS, T6SS	Imm-HEAT	*Repeats*: RHS
Ntox37	All-β; E, [KR] Hx[DH]	Actinobacteria, γ-proteobacteria, chlamydiae, chloroflexi, firmicutes	T2SS, T7SS(WXG)	Imm32	*Proteases:* Tox-PLOTU; *Repeats*: RHS
Ntox39	All-β; Several basic residues	Firmicutes	T2SS	-	*Repeats*: RHS
Ntox40	All-β; DRxxG, R, Y	Acidobacteria, actinobacteria, α,β,γ,ϵ-proteobacteria, bacteroidetes, firmicutes, planctomycetes, synergistetes, ***eukaryotes:*fungi**	T2SS, T5SS, T6SS, T7SS (WXG,LXG,LDXD), TcdB/TcaC	Imm35, Imm36, Imm59, Imm60, Imm61, Imm63	*Repeats*: RHS, FilH
Ntox42	α + β; GK, ExxxH, DxYxF[ED]	Firmicutes (negativicutes)	T5SS	-	*Repeats*: FilH
Ntox44	All-α; DxK, GNxxxG, and DxxxD.	Actinobacteria, α,β,γ,δ-proteobacteria, bacteroidetes, chloroflexi, firmicutes, proteobacteria, spirochaetes, ***eukaryotes:*fungi (microsporidia)**	T2SS, T6SS, T7SS(WXG,LXG)	-	*Proteases:* Papain-like protease; *Repeats*: RHS, ALF
**Predicted RNase toxins with two conserved histidine residues**					
Tox-EDA39C	α + β; H, Sx[HS]Y; Present in a wide range of eukaryotes where it might be a defensive RNAse	Acidobacteria, actinobacteria, α,β,γ,δ-proteobacteria, bacteroidetes, chlamydiae, chloroflexi, firmicutes, gemmatimonadetes, planctomycetes, verrucomicrobia, ***eukaryotes:*plants, chlorophytes, fungi, dictyosteliida, stramenopiles**	T2SS, T5SS, T6SS, T7SS (LXG)	Imm-SuFu	*Proteases:* HINT; *Repeats*: RHS
Ntox18	α/β; H, S, H	α,β,γ- proteobacteria, bacteroidetes, chloroflexi, cyanobacteria, firmicutes, ***eukaryotes:*metazoan: Lateral transfer to*Branchiostoma***	T2SS (MafBN), T2SS	Imm29, Imm42; Imm29 association is widespread across bacteria	*Proteases:* HINT; *Repeats*: RHS, FilH
Ntox22	Mostly β, D, D, H, E, H	*Ralstonia*, *Burkholderia phymatum*	T5SS	-	*Repeats*: FilH
Ntox26	α + β; KHxx[DE], Q, W, H	Actinobacteria, α,β,γ-proteobacteria, firmicutes, fusobacteria	T2SS, T5SS T7SS (LXG)	-	*Proteases:* HINT; *Repeats*: RHS, FilH, Tail fiber
Ntox30	All-β; RxH, R THIP	Actinobacteria, bacteroidetes, α,γ-proteobacteria, firmicutes, spirochaetes	T2SS, T6SS, T7SS (WXG, LXG), TcdB/TcaC	-	*Repeats*: RHS
Ntox43; *Pseudomonas* RhsT-C belongs to this clade	α + β; with two conserved H	Actinobacteria, γ,δ-proteobacteria, firmicutes, verrucomicrobia	T2SS, TcdB/TcaC	-	*Repeats*: RHS
Tox-JAB-1	Deaminase fold (α + β); NxxxE, HxH, S, D	Bacteroidetes	T2SS	Imm65	*Repeats*: RHS
Tox-JAB-2 (DUF4329 in Pfam)	Deaminase fold (α + β); E, H[ST]H, S, D	α,γ,δ-proteobacteria bacteroidetes, cyanobacteria, firmicutes, ***eukaryotes:*fungi (ascomycota),*viruses:*caudovirales**	T2SS, T6SS, T7SS (WXG), TcdB/TcaC	Imm-NTF2 family 2	*Repeats*: RHS
Tox-ComI	α + β fold; DE motif	Actinobacteria, α,β,γ-proteobacteria, bacteroidetes, firmicutes, verrucomicrobia, ***eukaryotes:*dictyosteliida, fungi (ascomycota, basidiomycota),*viruses: Bacillus*phage SP10**	T2SS, T6SS	Imm-ComJ, Imm-SUKH	*Proteases:* HINT; *Repeats*: RHS
Tox-HET-C	All-α; H, [DE], HxD, HxxxDxxxH, Nxx[DE], [ST]G; We predict that the Het-C domain is related to phospholipase C and the S1-P1 nuclease and shares a common active site and fold (see text)	Actinobacteria, cyanobacteria, γ,δ-proteobacteria, dictyoglomi, *eukaryotes:***fungi (ascomycota, basidiomycota), metazoa**	T2SS, T6SS, PVC	-	*Proteases:* PVC-Metallopeptidase
Ntox29	All-β; D,D, HxE, D, K, R residues	β,γ-proteobacteria, firmicutes	T2SS, T5SS,T7SS (LXG)	Imm41	*Proteases:* HINT; *Repeats*: RHS, FilH
**Predicted RNase toxins with uncertain metal dependence**					
Ntox1	α + β fold; C, C, H, E	Acidobacteria, α-proteobacteria	PVC		*Proteases:* PVC-Metallopeptidase
Ntox3	All-β; several charged residues including as D, R, H, C; associated with Annexin domain in *Haliangium*	*Haliangium* (δ-proteobacteria)*, Microscilla* (Bacteroidetes)	PVC	-	*Proteases:* PVC- Metallopeptidase; *Repeats*: Annexin
Ntox6	α + β; several charged residues;	*Microcoleus*(Cyanobacteria)*, Haliangium(δ-proteobacteria)*	PVC	-	*Proteases:* PVC- Metallopeptidase
Ntox8	α + β fold; HxR and HxxxH motifs	β-proteobacteria, bacteroidetes, firmicutes, ***eukaryotes:*dictyosteliida**	T2SS, T6SS	Imm16	*Repeats*: RHS
Ntox10	α + β; Several charged residues	Bacteroidetes, verrucomicrobia	T2SS	Imm27, Imm53; Imm27 primary immunity protein across most lineages	*Repeats*: RHS
					*Proteases:* Transglutaminase
Ntox11	α/β followed by β rich C-terminus; N-terminal GxR, RxxxoH motif, C-terminal domain has H, GxE, GxxH and an acidic residues; *Naegleria* possibly secreted	Actinobacteria, cyanobacteria, firmicutes α, δ,γ-proteobacteria, ***eukaryotes: Trichoplax, Naegleria***	PVC	-	*Proteases:* PVC- Metallopeptidase
Ntox14	α + β; Several charged residues	*Desulfobacca, Pelobacter* (δ-proteobacteria)	PVC	Imm22	*Proteases:* PVC-Metallopeptidase
Ntox33	α + β; [DN]xHxxK, DxxxD	Actinobacteria, cyanobacteria, firmicutes, γ-proteobacteria, verrucomicrobia	T2SS	-	-
Ntox45	α + β; DxD motif	Actinobacteria, α-proteobacteria, bacteroidetes	T2SS	-	*Proteases:* HINT; *Repeats*: RHS
**Other toxins that act on nucleic acids**					
Tox-Deaminase	Deaminase fold (α + β); [HCD]xE, CxxC; As previously reported, nine distinct families of deaminase belonging to two distinct clades are present in polymorphic toxin systems as toxins. We report two additional families below	Acidobacteria, actinobacteria, bacteroidetes, chlorobi, cyanobacteria, firmicutes, α,β,γ,δ,-proteobacteria ***Eukaryotes*: See text and previous publication**	T2SS (MafBN), T5SS, T6SS, T7SS (WXG, LDXD, LXG), PVC, TcdB/TcaC	Imm1, Imm2, Imm3, Imm4, Imm5,	*Proteases:* PVC-Metallopeptidase, HINT, CPD, PrsW peptidase, Caspase; *Repeats*: RHS, FilH, ALF, PPR
				Imm6, Imm10, Imm18, Imm-SUKH, Imm-ank	
Tox-Deaminase (sce3516-like)	Deaminase fold (α + β); H[occasionally D]xE, CxxC; Toxins of this family belong to the strand-hairpin clade of deaminases	Actinobacteria, β,γ,δ,-proteobacteria	T2SS, T5SS, T6SS T7SS, TcdB/TcaC	Imm-SUKH	*Proteases:* HINT
					*Repeats*: RHS, FilH
Tox-Deaminase (WD0512-like)	Deaminase fold (α + β); CxE, CxxC; Toxins of this family belong to the Helix-4 clade of deaminases. These proteins additionally contain a C-terminal toxin, the Tox-Latrotoxin-CTD	α- proteobacteria (*Wolbachia*)	T2SS	-	*Repeats*: RHS
Tox-ParB	ParB fold (α + β); R	Actinobacteria, α,β,γ,δ-proteobacteria, bacteroidetes, firmicutes	T2SS (MafBN), T5SS, T6SS, T7SS (WXG), PVC	Imm20, Imm27, Imm-SuFu	*Proteases:* PVC-Metallopeptidase, HINT; *Repeats*: RHS, FilH
Tox-ParBL1	Predicted ParB fold (α + β); [ST], [NT][RT][RT]; note the latter two residues of this motif are mostly R	Actinobacteria,α,β,γ-proteobacteria, firmicutes, euryarchaea, ***eukaryotes:*stramenopiles, viridiplantae, ascomycota, chlorophyta, choanoflagellida,metazoa,ciliophora, kinetoplastida**	T2SS (MafBN), T5SS, T6SS, T7SS (WXG, LXG)	Imm-SUKH, Imm44	*Proteases:* HINT; *Repeats*: FilH, RHS
Tox-HTH	HTH fold; RxxY, R, [ST]	Acidobacteria, actinobacteria, α,β,γ,δ,ϵ-proteobacteria, bacteroidetes, cyanobacteria, firmicutes, proteobacteria, archaea, ***eukaryotes:*ascomycota, viridiplantae,**	T2SS, T5SS, T6SS, T7SS (LXG, WXG, LDXD), PVC, MuF	-	*Proteases:* PVC-Metallopeptidase; *Repeats*: FilH
**Peptidase toxins**					
Tox-ALFMetallopeptidase(Anthrax lethal factor)	metallopeptidase fold (α + β); HExxH	Actinobacteria, bacteroidetes, δ-proteobacteria, firmicutes, fibrobacteres	PVC, T2SS	Imm-SuFu	*Proteases:* PVC-Metallopeptidase
					*Repeats*: FilH
Tox-HopH1	metallopeptidase fold (α + β); HExxH, [DE]N	Actinobacteria, α,β,γ-proteobacteria bacteroidetes, planctomycetes	T2SS,T3SS,T5SS, T6SS,T7SS (WXG), PVC, TcdB/TcaC	-	*Proteases:* PVC-Metallopeptidase, ZU5, caspase; *Repeats*: RHS
Tox-MPTase1	metallopeptidase fold (α + β); HExxH	Actinobacteria, α,β,γ,δ-proteobacteria, bacteroidetes, chlorobi, cyanobacteria, deinococci, planctomycetes, spirochaetes, thermotogae	T2SS,T7SS (WXG), TcdB/TcaC	-	*Repeats*: RHS
Tox-MPTase2	metallopeptidase fold (α + β); Y, HExxH,	Bacteroidetes	TcdB/TcaC	-	*Proteases:* ZU5; *Repeats*: RHS
Tox-MPTase3	metallopeptidase fold (α + β); K, HExxH, F[DE]	α-proteobacteria, bacteroidetes	T2SS, PVC	-	*Proteases:* PVC-Metallopeptidase; *Repeats*: RHS
Tox-MPTase4	metallopeptidase fold (α + β); F[DN], [RK], HExxH	γ-proteobacteria, fusobacteria, firmicutes, planctomycetes	T2SS, T6SS, T7SS (WXG,LDXD,LXG)	-	*Repeats*: RHS
Tox-MPTase5	metallopeptidase fold (α + β); HEELH	Actinobacteria, γ-proteobacteria	T2SS	-	*Repeats*: RHS
PVC-Metallopeptidase	metallopeptidase fold (α + β); HExxH; Most versions of this domain are releasing peptidases in polymorphic toxins. However, some versions, often present at the C-terminal end of polymorphic toxins, are likely to additionally function as toxins	Acidobacteria, actinobacteria, α,β,γ,δ-proteobacteria, bacteroidetes, chlorobi, chloroflexi, cyanobacteria, deinococci, firmicutes, nitrospirae, verrucomicrobia, *archaea:* euryarchaeota, ***eukaryotes:*fungi(ascomycota)**	PVC	-	*Proteases:* PVC-Metallopeptidase; *Repeats*: RHS
Tox-MCF1-SHE	All-α; S, T, HSxxE	Actinobacteria, α,β,γ,δ-proteobacteria, bacteroidetes, chlamydiae, ***viruses:*Acanthamoeba polyphaga mimivirus**	T2SS, T7SS(WXG), PVC	-	*Proteases:* PVC-Metallopeptidase, Caspase, Tox-PLOTU
Tox-SerPeptidase	α + β; H, R, R	Actinobacteria, α,β,γ,δ,ϵ-proteobacteria	T2SS, T7SS (WXG)	-	*Proteases:* Tox-PLOTU
Tox-YabG	α + β; HxD, Y, E, [DE], GHD, Y, R	Bacteroidetes, firmicutes	PVC	DUF1021(antitoxin in toxin-antitoxin systems), Imm-SUKH	*Proteases:* PVC-Metallopeptidase
Tox-LD-peptidase	LD-peptidase (PDB: 1ZAT); H, S, C	Actinobacteria, bacteroidetes, β,γ,δ-proteobacteria, chloroflexi, firmicutes	T2SS,T6SS, TcdB/TcaC	Imm16, Imm57	*Proteases:* ZU5; *Repeats*: RHS
Tox-Caspase	Caspase-like fold (α/β); H, C; Most versions of this domain are releasing peptidases in polymorphic toxins. However, some versions, often present at the C-terminal end of polymorphic toxins, are likely to additionally function as toxins	Actinobacteria, α,β,γ,δ,ϵ-proteobacteria, bacteroidetes, chloroflexi, cyanobacteria, firmicutes, ***viruses:*caudovirales**	T2SS,T6SS, T7SS (WXG,PPE), PVC	Imm36	*Proteases:* PVC-Metallopeptidase; *Repeats*: RHS
Tox-HDC	α + β; H, D, C	β,γ-proteobacteria, ***viruses:*caudovirales**	T2SS	-	*Proteases:* Caspase; *Repeats*: RHS
Tox-NLPC/P60	Papain-like peptidase fold (α + β); C, H, D	Bacteroidetes, δ-proteobacteria	T6SS, PVC, TcdB/TcaC	-	*Proteases:* PVC-Metallopeptidase, ZU5; *Repeats*: RHS
Tox-PL1	Papain-like peptidase fold (α + β); NC, H, D; Most versions of this domain are toxins in polymorphic toxins. However, some versions are, additionally, likely to be releasing peptidases	Actinobacteria, bacteroidetes, γ,δ-proteobacteria, firmicutes, fusobacteria, gemmatimonadetes	T2SS, T3SS, T6SS, T7SS (WXG), MuF	-	*Proteases*: Tox-Caspase, HINT; *Repeats*: RHS
Tox-PL-2	Papain-like peptidase fold (α + β); C, NxxH, DN	β,δ-proteobacteria, cyanobacteria, firmicutes	T2SS, TcdB/TcaC	Imm73	*Proteases:* HINT, PLOTU, ZU5; *Repeats*: RHS
Tox-PL3	Papain-like peptidase fold (α + β); C, [DE]H, [DE], R	Bacteroidetes, fibrobacteres, δ,ϵ-proteobacteria	T2SS, TcdB/TcaC	-	*Proteases:* ZU5; *Repeats*: RHS
Tox-PLOTU	Papain-like peptidase fold (α + β); C, H, D; Most versions of this domain are releasing peptidases in polymorphic toxins. However, some versions, often present at the C-terminal end of polymorphic toxins, are likely to additionally function as toxins	Actinobacteria, α,γ-proteobacteria, bacteroidetes, chlamydiae, ***eukaryotes:*fungi (ascomycota), metazoa, viridiplantae,*viruses:*Invertebrate iridescent virus 3, Wiseana iridescent virus**	T2SS (APD1), T7SS (WXG)	-	*Repeats*: Ankyrin, Sel1, FilH
Tox-PLC39	Papain-like peptidase fold (α + β); C, H, D	Bacteroidetes, chloroflexi, firmicutes	T2SS, T6SS, PVC	-	*Proteases:* PVC-Metallopeptidase; *Repeats*: RHS
Tox-PLDMTX	Papain-like peptidase fold (α + β); C, W, H, D, Q	α,β,γ-proteobacteria	T2SS	-	-
Tox-TGase	Papain-like fold (α + β); C, H, D	β,γ,δ-proteobacteria, bacteroidetes, cyanobacteria	T2SS, T3SS, PVC	-	*Proteases:* PVC-Metallopeptidase
Tox-UCH	Papain-like fold (α + β) C, H, D	β-proteobacteria	PVC	-	*Proteases:* PVC-Metallopeptidase
Tox-OmpA	α + β;	α,β,γ-proteobacteria, cyanobacteria	PVC	-	*Proteases:* PVC-Metallopeptidase
**Protein-modifying toxins**					
Tox-ART-RSE;	ADP-ribosyltransferase fold (α + β); RxDxR, S, [DN]xN, E	Actinobacteria, α,β,γ,δ-proteobacteria, bacteroidetes, chloroflexi, firmicutes, planctomycetes, spirochaetes, tenericutes**,*eukaryotes:*fungi (ascomycota, basidiomycota), metazoan (hexapoda, mollusca), viridiplantae,*viruses:*Vibrio phage CTX**	T2SS, T6SS, T7SS (WXG, LXG, LDXD)	Imm41, Imm-ADP-RGHD (ADP-ribosyl glycohydrolase)	*Proteases:* HINT, Caspase, MCF1-SHE *Repeats*: RHS, Tail-fiber
Tox-ART-PARP	ADP-ribosyltransferase fold (α + β); HG[ST], Y, K, E	Actinobacteria	PVC	-	*Proteases:* PVC-Metallopeptidase
Tox-ART-HYE1	ADP-ribosyltransferase fold (α + β); H, Y, E	γ-proteobacteria	TcdB/TcaC?	-	*Repeats*: RHS
Tox-ART-HYD1	ADP-ribosyltransferase fold (α + β); H,[RK], [FY], [DE]	Actinobacteria, β,γ-proteobacteria, bacteroidetes, firmicutes	T2SS, T6SS, T7SS	Imm-My6CBD;	*Proteases*: HINT*; Repeats*: RHS
Tox-ART-HYD2	ADP-ribosyltransferase fold (α + β); H, D, GFY, W, R	Actinobacteria, bacteroidetes, deinococci, fibrobacteres, firmicutes, fusobacteria, γ-proteobacteria, lentisphaerae, spirochaetes, synergistetes, ***eukaryotes:*choanoflagellida, Capsaspora, fungi, cnidaria**	T2SS, PVC	-	*Proteases:* HINT, PVC-Metallopeptidase; *Repeats*: RHS, Tail-Fiber
Tox-ARC (ADP-Ribosyl cyclase)	Flavodoxin fold (α/β); [ST] [DE], S, E	Actinobacteria, bacteroidetes, cyanobacteria, firmicutes, β, γ- proteobacteria, spirochaetes ***eukaryotes:*fungi (ascomycota, basidiomycota),*Capsaspora*, choanoflagellida, metazoa;** This domain appears to have independently been acquired by the fungi and the animals from the bacteria.	T2SS, T5SS, T6SS, T7SS (LXG, WXG)	Imm74, Imm63; Imm74 is the primary immunity protein across wide phyletic range	*Repeats*: RHS, FilH
Tox-Doc	Doc/Fic fold (PDB: 2f6s, All-α); HxFx[DE]GNxR; (See Pfam PF02661)	Actinobacteria, γ-proteobacteria	T5SS, T7SS (WXG)	Imm23, Imm-SUKH, Imm13	*Proteases:* Caspase; *Repeats*: FilH
Tox-CNF (Cytotoxic necrotizing factor)	CNF1/YfiH fold (α + β, PDB: 1hzg); D, C, H; See Pfam PF05785	γ-proteobacteria	T6SS	-	*Repeats*: RHS
Tox-Glycosyltransferase	Nucleotide diphospho-sugar transferase fold (α/β); [DNE]xxR, YxDxD; See Pfam PF04488	Actinobacteria	T7SS (WXG), PVC	-	*Proteases:* PVC-Metallopeptidase
Tox-Peptide Kinase	α + β; DxH, YKP[KR], DxHxEN, DxE, S, R; Related to the kinase domain found in lantibiotic synthetases	Firmicutes	PVC	-	*Proteases: PVC-Metallopeptidase*
**Pore-forming toxins**					
Tox-WTIP	Two membrane spanning α-helices; RxxR, Wx[ST]IP	α,β,γ-proteobacteria	T2SS, PVC	-	*Proteases:* PVC-Metallopeptidase; *Repeats*: RHS
**Toxins that act on carbohydrates**					
Tox-Aldo-ketoreductase	Rossmann (α/β);	Bacteroidetes, cyanobacteria	PVC	-	*Proteases:* PVC-Metallopeptidases;
Tox-Glucosaminidase	Lysozyme-like fold (α + β); E, N, Y (See Pfam PF01832)	Firmicutes	T6SS, PVC	-	*Proteases:* PVC-Metallopeptidase
**Toxins that act on lipids**					
Tox-AB hydrolase1 (Pfam DUF2235)	α/β hydrolase (α/β); DG, [ST]N, [KR], D, ExE, GxHxD	Acidobacteria, actinobacteria, α,β,γ,δ,ϵ-proteobacteria, bacteroidetes, cyanobacteria, nitrospirae planctomycetes, verrucomicrobia, ***eukaryotes:*fungi(ascomycota, basidiomycota), rhodophyta, viridiplantae**	T2SS, T6SS	-	*Repeats*: RHS
Tox- AB hydrolase3	α/β hydrolase (α/β); G[ST], GHSxG	Actinobacteria, α,β,γ-proteobacteria, bacteroidetes, firmicutes	T2SS, T6SS,T7SS (WXG), TcdB/TcaC	Imm66, Imm69	*Repeats*: RHS, FilH
Tox-PLA2	Phospholipase A2 fold (All-α, PDB: 1kp4); DxC[ST], CxxHxxxYxN, C	Actinobacteria, α,β,γ,δ-proteobacteria, aquificae, bacteroidetes, chlorobi, chloroflexi, cyanobacteria, deinococci, firmicutes, fusobacteria, nitrospirae, planctomycetes, spirochaetes, ***eukaryotes:*fungi(ascomycota), heterolobosea, metazoa, stramenopiles, viridiplantae,*Viruses:*Campylobacter phage**	T2SS	-	*Repeats*: RHS, ALF
Tox-CDP-alcohol phosphatidyltransferase	All-α; DxxDGxxxR, DxxxD; See Pfam PF01066	β-proteobacteria (mainly Neisseria species)	PVC	-	*Proteases:* PVC-Metallopeptidase
Tox-Glycerophosphoryl diester phosphodiesterase (GDPD)	TIM Barrel (PDB: 1VD6; α/β); HRG, E, ExD, D, H; See Pfam PF03009	*Cyanothece* sp. (Cyanobacteria)	PVC	-	*Proteases:* PVC-Metallopeptidase
**Miscellaneous toxins**					
Tox-AB hydrolase2	α/β hydrolase superfamily (α/β); NG, [DE], [KR], HSxG, D, H	acidobacteria, α,β,γ,δ,ϵ-proteobacteria, chlamydiae, fusobacteria, verrucomicrobia, ***eukaryotes:*fungi(ascomycota, basidiomycota), stramenopiles**	T2SS, T5SS, T6SS	Imm-SUKH	*Repeats*: FilH, RHS
Tox-ODYAM1	All-α; Several charged residues	α-proteobacteria, bacteroidetes	T2SS (APD1)	-	*Proteases:* Tox-PLOTU; *Repeats*: Sel1
Tox-LatrotoxinCTD	Two conserved α-helices; D, [ST], Y, E	α,γ-proteobacteria, ***eukaryotes:*metazoa (*Latrodectus hasseltii, Latrodectus tredecimguttatus*)**	T2SS	-	*Proteases:* Tox-PLOTU; *Repeats*: ankyrin
Tox-SGS (salivary gland secreted protein)	α + β; C, C, C, C, [DE}xx[ND]	***Eukaryotes:*metazoan (crustacea, hexapoda)**	T2SS	-	*Repeats*: RHS
Ntox38	All-β; PXhhG and several hydrophobic residues	Actinobacteria	T2SS, T7SS (WXG)	Imm56	*Proteases:* Mycosin (Subtilisin)-like protease in the neighborhood
Ntox46	α + β; [KR]STxxPxxDxx[ST], Q	α,γ,δ-proteobacteria, bacteroidetes	T2SS, T6SS	-	*Repeats*: RHS, FilH

1. Toxins are grouped and arranged based on the similarity of their known or predicted biochemical functions.

2. Where possible, known or predicted folds are described. The folds are further classified as All-α (composed entirely of α-helices), All-β (composed entirely of β-strands), α + β (Containing α-helices and β-strands) or α/β (comprising repeated α-helix-β-strand units) depending on the arrangement of their structural elements. Individual conserved residues and motifs are separated by commas. Alternative residues are enclosed in square brackets; ‘x’ denotes any residue, ‘h’ indicates a hydrophobic residue (LIYVFMCW).

3. By default most lineages are bacterial unless stated otherwise. Eukaryotes and viruses are shown in bold.

4. T2SS: Type 2 secretion system; T5SS: Type 5 secretion system, T6SS: Type 6 secretion system, T7SS: Type 7 secretion system. The secretory domains for T7SS are shown next to it in parentheses.

**Table 3:** Phyletic distribution and associated toxins of Immunity proteins associated with polymorphic toxin systems

**Immunity protein**	**Fold; Conservation**^**1**^	**Associated toxins**^**2**^	**Phyletic distribution**	**Additional Notes**
Imm-SUKH	α + β (PDB: 3D5P); Several hydrophobic residues and family-specific differences. Refer to previous paper for details	*HNH fold families:* Tox-SHH,Tox-HNH,Tox-HNH-CIDE, Tox-WHH, Tox-DHNNK, Tox-LHH, Tox-GHH, Tox-HHH, Tox-NucA, Tox-ColE7;	Acidobacteria, actinobacteria, αβγδϵ-proteobacteria, bacteroidetes,, chloroflexi, cyanobacteria, deinococci, firmicutes, fusobacteria, planctomycetes, spirochaetes, synergistetes, verrucomicrobia ***Eukaryotes: Giardia*, ciliophora, choanoflagellida, fungi,*Naegleria*, metazoa, stramenopiles, viridiplantae, chlorophyta, eukaryotic viruses**	This superfamily comprises 5 major families (SUKH1-5), which have been combined in this study; Shows fusions on occasions to toxins and immunity domains; For e.g. fusions to Tox-GHH, Imm-SuFu, Imm33, Imm37, Imm66, Imm67, Imm68, Imm69. Found in homogeneous and heterogenous polyimmunity loci
		*Restriction endonuclease fold families:* Tox-REase-4, Tox-REase-3;		
		*Deaminase families:* YwqJ, XOO2897, BURPS668_1122		
		Proteases: YabG, Tox-PL1; *Other toxins:* Tox-EndoU, Tox-DOC, Caspase, Tox-ParBL1, Tox-ComI, Ntox15, Ntox20, Tox-ABhydrolase2, Tox-ABhydrolase3		
Imm-SuFu	α + β (PDB: 1M1L); GxS, E, E, DxxR	NGO1392-like Tox-HNH fold domain^a^ (SuFu-associated nuclease), Tox-GHE^b^, Tox-ParB^c^, Tox-DHNNK ^d^, Tox-AHH^e^, Tox-HNH^f^, Tox-EndoU^g^, Tox-EDA39C^h^, Tox-PL-C39-like peptidase^i^,Tox- ALF-MPTase^j^, Ntox7^k^	Acidobacteria, actinobacteria^ab,d^, α,β^a,b,c, f^,γa^,c, d^,δ^e, h^,ϵ-proteobacteria, bacteroidetes^b,I,k^, chloroflexi, firmicutes^b,e,g^, fusobacteria, planctomycetes, spirochaetes^j^, tenericutes verrucomicrobia.	Fused to members of the SUKH family, ankyrin repeats, Imm5, Imm11, Imm33, Imm36, Imm66, Imm67, Imm68, Imm69, PsbP/MOG1. Found in homo- and heterogeneous polyimmunity loci. See Pfam PF05076
			***Eukaryotes:*chlorophyta, ascomycota, choanoflagellida, metazoa**	
Imm-SuFu- family 2	α + β; [ST]xxG, [DE]	Tox-ColE7^a^, Tox-DHNNK, Tox-HNH fold^b^, Tox-ALFMPTase^c^,Tox-GDPD^d^	actinobacteria α^d^,β,γ,δ,ϵ-proteobacteria, bacteroidetes, cyanobacteria, fibrobacteres, firmicutes ^a,b^, fusobacteria, gammaproteobacteria, planctomycetes, proteobacteria, spirochaetes^c^, verrucomicrobia	Fused to Imm34, Imm33, Imm66, Imm67, Imm68, Imm69; Found in heterogeneous polyimmunity loci
Imm-Cloacin	FKBP-like α + β; EYSxD, NxG	Tox-ColE3^a^	Plasmid ^a^,ColE6-CT14 ^a^, γ-proteobacteria ^a^	
HEAT repeats	All α;	Tox-REase-7^a^	Actinobacteria^a^,bacteroidetes,cyanobacteria,γ-proteobacteria,planctomycetes^a^,verrucomicrobia^a^	
Ankyrin repeats (Imm-ank)	All α;	Tox-AHH^a^	Firmicutes^a^, planctomycetes^a^, γ-proteobacteria^a^	Fused to SuFu-like immunity domains in firmicutes and found in heterogeneous polyimmunity loci
LRR-repeats	α/β;	Next to T5SS^a^ toxins	actinobacteria,bacteria,β,γ^a^,ϵ-proteobacteria, firmicutes, tenericutes	Found in heterogeneous polyimmunity loci
Imm-CdiI	Two transmembrane helices; several hydrophobic residues	CdiAC	γ-proteobacteria	
Imm-NTF2	NTF2 fold (α + β); W, W, W	Tox-NucA ^a^	Bacteroidetes, β,γ ^a^,ϵ-proteobacteria, firmicutes, fusobacteria, verrucomicrobia	Fused to ankyrin repeats and Imm13 in some proteins
Imm-NTF2-2	NTF2 fold (α + β); Y,W	Tox-JAB-2	γ –proteobacteria (*E. coli* only)	Although related in structure to Imm-NTF2, the sequences are quite divergent from each other
Imm-PA2201	Two all-α domains(PDB: 2FEF); D, W,GxWxxE, D, YPxD	Tox-REase-1^a^, Tox-AHH^b^	Bacteroidetes^a^, β ^a^,γ ^a, b^,ϵ ^a^ -proteobacteria, firmicutes ^a^	See Pfam DUF1910 + DUF1911
Imm-Barstar	α/β (PDB: 1BRS); DxxxD and several hydrophobic residues	Tox-Barnase-like ribonuclease^a^	Acidobacteria, actinobacteria^a^, α, β^a^,γ^a^,δ^a^,ϵ^a^-proteobacteria bacteroidetes^a^, chlamydiae^a^, chloroflexi^a^, cyanobacteria^a^, deinococci^a^, elusimicrobia, firmicutes^a^,fusobacteria^a^, nitrospirae^a^, planctomycetes^a^, verrucomicrobia, Archaea: euryarchaea^a^, ***Eukaryotes:*dictyosteliida,*Naegleria,*chlorophyta**	See Pfam PF01337
Imm-ADP-RGHD; ADP ribosyl glycohydrolase	All-α; (PDB: 1t5j); D, D[DE], [RK], H	Tox-ART-RSE^a^	acidobacteria, β,γ^a^-proteobacteria, firmicutes^a^	See Pfam Pf03747; an example of an enzymatic immunity protein
Imm-NMB0513	wHTH fold (α + β, PDB: 2O5H); W, W	Ntox20^a^, Ntox7^b^	betaproteobacteria^a,b^ gammaproteobacteria^a^	Corresponds to Pfam DUF596
Imm-ComJ	Mostly β; W, F[DE], PF, Y, Y	Tox-ComI-like competence nuclease^a^	α^a^β^a^γ^a^-proteobacteria, bacteroidetes^a^, cyanobacteria, firmicutes^a^,	
			***Eukaryotes:*viridiplantae**	
Imm-VC0424	α + β; α + β RRM fold, W at C-terminus	-	Firmicutes, fusobacteria, α,β,γ-proteobacteria	Also known as DUF1260 in the Pfam database. Only a subset of members is found in polymorphic toxin systems as potential immunity proteins. These species are listed in column 3
Imm-My6CBD	α + β; E, R, F, W	Tox-ART-HYD1^a^	actinobacteria ^a^, bacteroidetes ^a^, firmicutes ^a^, fusobacteria, β ^a^,γ ^a^ –proteobacteria, ***Eukaryotes*: Metazoa**	The type VI myosin cargo-binding domain of metazoa appears to have been acquired by lateral transfer from a bacterial version
Imm1	α + β; aromatic and W at C-terminus	SCP1.201 deaminases^a^	Actinobacteria^a^, bacteroidetes, cyanobacteria, firmicutes, planctomycetes α,β,γ-proteobacteria, verrucomicrobia	
Imm2	All α; acidic and hydrophobic residues	BURPS668_1122 deaminases	β, γ- proteobacteria	
Imm3	All α; charged, V	BURPS668_1122 deaminases	Firmicutes	found in heterogeneous polyimmunity loci
Imm4	α + β	SCP1.201 deaminases	*Burkholderia pseudomallei*	
Imm5	Mostly α; R, D	DYW deaminases^a^, CdiAC^b^	Actinobacteria^a^, bacteroidetes^a^, firmicutes^a^, α,β,γ^a, b^,proteobacteria	Fused to Imm36 on occasions
Imm6	Mostly α; P, [DE]	YwqJ deaminases^a^	Actinobacteria^a^, α-proteobacteria, firmicutes^a^	Found in homo and heterogeneous polyimmunity loci
Imm7	α + β; GxaG	Tox-REase-3^a^	actinobacteria, firmicutes ^a^, planctomycetes	
Imm8	α + β; WEa (a:aromatic) at C-terminus	Ntox7^a^	Acidobacteria, actinobacteria, bacteroidetes ^a^, firmicutes ^a^, α, β ^a^, γ ^a^, δ-proteobacteria	
Imm9	α + β; K and several conserved acidic residues	Tox-URI2	Bacteroidetes, γ-proteobacteria	Found in heterogeneous polyimmunity loci
Imm10	Mostly β; R and several hydrophobic residues	Pput_2613 deaminase^a^	actinobacteria bacteroidetes chloroflexi firmicutes β, γ^a^,δ,ϵ-proteobacteria; ***Eukaryotes:*ascomycetes**	Lateral transfer to fungi, found in heterogeneous polyimmunity loci
Imm11	α + β; several conserved hydrophobic residues	Tox-AHH^a^, Tox-HNH^b^, Tox-SHH^c^	Bacteroidetes^a^, chloroflexi, cyanobacteria, firmicutes^a^, planctomycetes^a^, α,β^a^,γ^a^,δ^a,b,c^,ϵ^a^-proteobacteria spirochaetes^a^ verrucomicrobia^a^	Listed in the Pfam database as DUF1629. Fused to SuFu on occasions. Found in heterogeneous and homogeneous polyimmunity loci.
Imm12	α + β; several conserved charged and hydrophobic residues	Tox-URI2^a^	Bacteroidetes^a^, spirochaetes	Found in heterogeneous polyimmunity loci
Imm13	α + β; D, D, D, D	Tox-DOC^a^	Actinobacteria, bacteroidetes cyanobacteria, firmicutes, fusobacteria^a^, spirochaetes, verrucomicrobia, α,β,γ,δ-proteobacteria,	Note lateral transfer to eukaryotes. Found in heterogeneous polyimmunity loci. Fused to Imm33 in some instances
			***Eukaryotes: Naegleria***	
Imm14	Mostly β; several hydrophobic residues	Tox-URI1^a^, Tox-HNH^b^	Actinobacteria^a^, α,β^a^,γ^a,^,δ^b^-proteobacteria, bacteroidetes^a^, chlamydiae^a^, chloroflexi^a^, cyanobacteria, firmicutes^a^, fusobacteria, spirochaetes, verrucomicrobia	Found in heterogeneous polyimmunity loci; Fused to Imm51 in one instance
Imm15	α + β; several polar and hydrophobic residues		Bacteroidetes, firmicutes, synergistetes	Found in heterogeneous polyimmunity loci
Imm16	α + β; several hydrophobic residues including a highly conserved W	Ntox8^a^	Actinobacteria, bacteroidetes ^a^, β ^a^,γ,δ-proteobacteria, firmicutes ^a^, planctomycetes, spirochaetes, verrucomicrobia	Also known as DUF2750
Imm17	Two TM helices; WxW and a R in the region between them		Bacteroidetes, firmicutes, fusobacteria, spirochaetes	Found in heterogeneous polyimmunity loci
Imm18	Mostly β; highly conserved D	Tox-HNH ^a^	Actinobacteria ^a^, αβ ^a^ γ ^a^ δ ^a^ -proteobacteria, bacteroidetes ^a^, firmicutes	Found in heterogeneous polyimmunity loci
Imm19	α + β; HxxRN motif and several conserved hydrophobic residues	-	Bacteroidetes	Found in heterogeneous polyimmunity loci
Imm20	α + β; several conserved hydrophobic residues	Tox-AHH ^a^, Tox-ParB ^b^	Acidobacteria, actinobacteria, bacteroidetes, β ^a, b^,γ ^a^,δ-proteobacteria, cyanobacterium firmicutes ^a^, fusobacteria, planctomycetes, spirochaetes, verrucomicrobia, ***Eukaryotes:*ascomycota**	Found in heterogeneous polyimmunity loci. Note presence in ascomycetes
Imm21	α + β; absolutely conserved WxG, YxxxC and several hydrophobic residues	NGO1392-like HNH fold^a^	Actinobacteria, α,δ-proteobacteria, bacteroidetes, firmicutes^a^, verrucomicrobia	Found in heterogeneous polyimmunity loci
Imm22	α + β; W, Y, and an acidic residue (mostly D)	ColD/E5 fold^a^, Tox-REase-4^b^, Ntox49^c^, Ntox14^d^	Actinobacteria, bacteroidetes^a,c^, β,γ-proteobacteria, firmicutes ^b,d^, fusobacteria, planctomycetes, verrucomicrobia, ***Eukaryotes:*ascomycota**	Previously known as SNCF1. Found in heterogeneous polyimmunity loci across a wide range of bacteria
Imm23	α + β; several hydrophobic residues including a WxW motif	Tox-AHH^a^, Tox-REase-7^b^	bacteroidetes^a^ cyanobacteria ^b^, firmicutes γ-proteobacteria verrucomicrobia	Some versions fused to Imm11; found in heterogeneous polyimmunity loci
Imm24	Mostly α-helical with C-terminal β-hairpin; several hydrophobics including a PxG motif (where x is mostly C)	Tox-AHH^a^, Tox-SHH^b^	Bacteroidetes^c^, β^a^,γ^a^,ϵ-proteobacteria, firmicutes^a,b^, verrucomicrobia	found in heterogeneous polyimmunity loci
Imm25	α + β; highly conserved in limited sequences	-	Bacteroidetes	Potential immunity protein found in heterogeneous polyimmunity loci, and a limited phyletic presence
Imm26	Mostly α; R and D and several hydrophobic residues	Tox-URI1^a^	Actinobacteria, bacteroidetes^a^, β,γ^a^,δ-proteobacteria, firmicutes, fusobacteria, planctomycetes, spirochaetes, ***Eukaryotes:*Ascomycota**	Note presence in ascomycetes, present in heterogeneous polyimmunity loci
Imm27	α + β; D, GGxP	Ntox10^a^, Tox-ParB^b^	Actinobacteria, bacteroidetes ^a^, β,δ^b^-proteobacteria, verrucomicrobia ^a^	Wide distribution but sporadic numbers
Imm28	Mostly α; acidic, P,G, R	Tox-WHH^a^, Tox-EndoU^b^, Ntox20^c^	Actinobacteria, α^a^,β^b,c^,γ^a^-proteobacteria	Note presence in *Odyssella*, present in heterogeneous polyimmunity loci
Imm29	Mostly α; R and acidic and several hydrophobic residues	Ntox18 ^a^	Actinobacteria, α ^a^,β ^a^,γ ^a^ -proteobacteria, bacteroidetes, firmicutes, fusobacteria	Note presence in *Odyssella*, present in heterogeneous polyimmunity loci
Imm30	Mostly α; Several conserved hydrophobics and DxG motif	Tox-SHH^a^	α ^a^,β^,^γ ^a^ –proteobacteria	Note presence in *Odyssella*. Limited number of hits, present in heterogeneous polyimmunity loci
Imm31	All-β; GxS, [R]	Ntox17^a^, Ntox7^b^	α ^a^,β^b^,γ ^a^,δ-proteobacteria, cyanobacteria	Note presence in *Odyssella*. Limited distribution
Imm32	α + β; H, and several conserved residues	Ntox12^a^, Ntox37 ^b^, Ntox7 ^c^	α ^a^,β,γ ^a,c^,δ-proteobacteria, chlamydiae, bacteroidetes ^b^, firmicutes ^a^, verrucomicrobia	Note presence in *Odyssella*, chlamydiae. Limited distribution
Imm33	Mostly β; W	Tox-HNH ^a^, Tox-DHNNK ^b^,^,^ NGO1392-like- HNH^c^	Acidobacteria, actinobacteria, αβ ^a,c^ γδ^c^-proteobacteria, bacteroidetes, chloroflexi, firmicutes, ^b^, fusobacteria, planctomycetes, ***Eukaryotes:*dictyosteliida**	Also known as DUF2185 in the Pfam database, fused to Imm- SUKH, Imm13, Imm34 and Imm-SuFu, Note presence in dictyosteliida where it is fused to Imm34, present in homo and heterogeneous polyimmunity loci	
Imm34	Mostly β; ExxW, C-terminal D	-	Actinobacteria, α,β,γ,δ,ϵ-proteobacteria, bacteroidetes, firmicutes, fusobacteria, planctomycetes, spirochaetes, verrucomicrobia, ***Eukaryotes:*dictyosteliida, heterolobosea, cnidaria**	Also known as DUF2314. Fused to Imm-SuFu family 2, Imm33, ankyrin repeats, TM helices, fusion to Imm33 appears to have occurred on multiple occasions independently, present in heterogeneous polyimmunity loci. Note presence in *Naegleria*, dictyosteliida and cnidarians. In dictyostellids, it is fused to Imm33	
Imm35	α + β; W, [ST]	Tox-PL1^a^, Ntox40^b^	Actinobacteria^a, b^, bacteroidetes^a^, β,γ^a^-proteobacteria, planctomycetes	Fused to Papain-like toxin and ADP-ribosyl glycohydrolase and Peptidase S8, in some instances. Possible protease inhibitor	
Imm36	BH3703-like fold (α + β); W, W	Tox-NucA^a^, DYW-Deaminase^b^, Ntox40^c^, Tox-CdiAC^d^, Tox-Caspase^e^	Actinobacteria^a, c, e^, α^a^,β^a^,γ^a,d^,δ-proteobacteria, bacteroidetes^a,b^, firmicutes^a^, fusobacteria, spirochaetes^a^	Also known as DUF600, fused to Tox-NucA, Imm-SuFu, Imm5, on occasions. Tox-NucA appears to be the primary toxin association. One of the large families. Found in homo and heterogeneous poly-immunity loci. Profile-profile analysis predicts a BH3703-like fold	
Imm37	α + β; ExG	Tox-WHH^a^	Acidobacteria, actinobacteria, αβγ^a^ϵ-proteobacteria, bacteroidetes, chloroflexi, cyanobacteria, deinococci, firmicutes^a^, fusobacteria^a^, planctomycetes, verrucomicrobia	Previously known as SNCF2, fused to SUKH in some instances. Found in heterogeneous polyimmunity loci	
Imm38	Mostly α; W at N and aromatic residue at C	Ntox19^a^, NGO1392-like- HNH^b^	Actinobacteria, bacteroidetes ^a^, β ^a,b^,γ ^a^,δ ^a^ -proteobacteria, firmicutes ^a^, fusobacteria ^a^, nitrospirae	Also known as DUF2247. Found in heterogeneous polyimmunity loci	
Imm39	α + β; GR, GxK and several polar and hydrophobic residues	Tox-URI2 ^a^	α ^a^ γ ^a^-proteobacteria	Limited distribution	
Imm40	α + β; GGD, F, W	Ntox19^a^	bacteroidetes^a^, chloroflexi firmicutes, β^a^,ϵ,γ^a^- proteobacteria		
Imm41	α + β; SF, W and several hydrophobic residues	Ntox21^a^, Ntox29 ^b^, Tox-ART-RSE ^c^	Actinobacteria, β ^a,b^,γ^c^,ϵ-proteobacteria, firmicutes, planctomycetes	Found in homo- and heterogeneous polyimmunity loci	
Imm42	α + β; Several conserved hydrophobic residues	Ntox18^a^	α,β ^a^,γ ^a^ -proteobacteria, firmicutes ^a^		
Imm43	α/β; W, P, D, S, R	Tox-AHH^a^	Bacteroidetes^a^, β-proteobacteria^a^, firmicutes	Found in heterogeneous polyimmunity loci	
Imm44	α + β; Multiple polar and hydrophobic residiues	Tox-URI1^a^, Tox-URI2^b^, Tox-ParBL1^c^	Bacteroidetes, β-proteobacteria^a,b^, firmicutes^c^	Limited phyletic distribution; Found in heterogeneous polyimmunity loci that show variations in structure even between closely related strains	
Imm45	α + β; C-terminal W	Tox-ColE3^a^	bacteroidetes,β ^a^,γ ^a^,ϵ-proteobacteria, firmicutes		
Imm46	α + β; E, W, E	-	Bacteroidetes, β-proteobacteria	Limited phyletic distribution. Found next to a predicted toxin	
Imm47	α + β; KxGDxxK	-	β-proteobacteria, firmicutes	Found in heterogeneous polyimmunity loci	
Imm48	All-α; HRG	-	Firmicutes,verrucomicrobia	Found in heterogeneous polyimmunity loci	
Imm49	All α; Hydrophobic residues, P	Tox-REase-1^a^, Tox-REase-6^b^	Actinobacteria ^b^, Bacteroidetes ^a,b^, cyanobacteria ^b^, firmicutes ^a^, fusobacteria ^a^, planctomycetes, β ^a,b^,δ,γ ^a,b^ -proteobacteria	Also known as DUF556	
Imm50	Mostly β; Several hydrophobic residues	Tox-HHH^a^, Ntox24^b^	actinobacteria, bacteroidetes^a^, firmicutes^a^, planctomycetes, α,β^a,b^,γ^a^-proteobacteria, verrucomicrobia		
Imm51	α + β; W, Dx[DE] and several hydrophobic residues	Tox-RES^a^, Tox-URI1^b^	Actinobacteria, bacteroidetes^a^, β,γ-proteobacteria, cyanobacteria,firmicutes ^b^, fusobacteria, spirochaetes	Fused to Imm14 on one occasion, Found in polyimmunity loci	
Imm52	α + β; W,GT,F	Tox-REase-5^a^	**Caudoviruses**^**a**^, α,β ^a^,γ ^a^,δ ^a^ –proteobacteria		
Imm53	α + β (Central β-sheet with flanking α-helices); W, WE, PGW, W	Ntox24^a^, Ntox10^b^	Acidobacteria, actinobacteria, α,β,γ,δ,ϵ-proteobacteria, bacteroidetes, chlamydiae ^b^, cyanobacteria, firmicutes ^a^, spirochaetes, verrucomicrobia		
Imm54	α + β; GF, Q	Tox-REase-9^a^, Tox-RelE^b^, Tox-URI^c^, Tox-REase-4^d^, Tox-REase-7^e^, Tox-REase-10^f^	actinobacteria, bacteroidetes ^a, c, d^, chlamydiae ^a^, firmicutes ^a, c, d,e^, fusobacteria^b,f^, planctomycetes, α,β ^c^,γ ^a^,δ,ϵ-proteobacteria, spirochaetes, verrucomicrobia	Found in heterogeneous polyimmunity loci	
Imm55	α + β; G and several hydrophobic residues	Tox-SHH^a^	actinobacteria, bacteroidetes^a^, cyanobacteria^a^, firmicutes^a^, lentisphaerae, planctomycetes, α,β,γ^a^-proteobacteria, synergistetes, verrucomicrobia		
Imm56	α + β; D, GR	Ntox38^a^, Tox-HNH^b^	Actinobacteria ^a,b^,		
			chloroflexi ^a^		
Imm57	Mostly α; D, SE, C	Tox-LD-peptidase^a^, Tox-Caspase^b^	β^a^,γ^a, b^-proteobacteria		
Imm58	α + β; YxxxD, WxG, KxxxE	Unknown toxins with RHS repeats	β,δ -proteobacteria	Limited distribution	
Imm59	α + β (Central β-sheet with flanking α-helices); [DE]R motif	Ntox13^a^, Ntox40^b^	firmicutes ^a,b^	Fused to Imm63 on some instances	
Imm60	Mostly β; N, W	Ntox40 ^a^, Ntox48^b^	bacteroidetes	Found in heterogeneous polyimmunity loci	
			firmicutes ^a^, fusobacteria, α ^b^,γ^b^ –proteobacteria,		
			euryarchaea		
Imm61	α + β; R	Ntox40^a^	actinobacteria ^a^		
Imm62	α + β; -(mostly E), W	Ntox31^a^, Ntox48^b^	Firmicutes^a, b^,	Found in heterogeneous polyimmunity loci	
			γ-proteobacteria		
Imm63	α + β; E + G, -(mostly E)xxY	Ntox40^a^, Tox-CdiAC^b^, Tox-ARC	actinobacteria ^a,c^	Found in polyimmunity loci	
			bacteroidetes		
			firmicutes^a^, β,γ^a,b^ -proteobaceria		
Imm64	α + β; DxEA, R motifs	Tox-ColD^a^	Euryarchaea^a^, firmicutes^a^, ϵ-proteobacteria		
Imm65	α + β; YxC, and several charged residues	Tox-JAB-1	Bacteroidetes	Contains a signal peptide and a lipbox	
Imm66	Mostly α; D, W, F, Y,W	Tox-ABhydrolase3^a^, Ntox48^b^	Actinobacteria, bacteroidetes, cyanobacteria, firmicutes	Fused to one or more immunity domains such as Imm68, SUKH, Imm-SuFu- family 2, Imm33, Imm69, Imm67, Imm-SuFu, Imm66, and TPR repeats. Some proteins in firmicutes have up to 10 immunity domains	
			Fusobacteria, α,β ^a^,γ^b^,ϵ-proteobacteria, spirochaetes, verrucomicrobia, ***Eukaryotes:*Ascomycota, viridiplantae**		
Imm67	α + β; W, E, W	-	actinobacteria, bacteroidetes, chloroflexi, cyanobacteria, firmicutes, fusobacteria, planctomycetes, α,β,γ,δ, ϵ-proteobacteria, spirochaetes, verrucomicrobia	Fused to one or more immunity domains such as Imm68, Imm33, Imm-SUKH, Imm-SuFu-family 2, Imm69, Imm-SuFu, Imm66, Imm67, TPR and ankyrin repeats. Some proteins in firmicutes have up to 10 immunity domains	
Imm68	α + β; E	-	actinobacteria, bacteroidetes, firmicutes, spirochaetes	Fused to one or more immunity domains such as Imm-SUKH, Imm-SuFu, Imm67, Imm66, Imm-SuFu-family 2, Imm69, Imm33, Imm68 andTPR repeats. Some proteins in firmicutes have up to 10 immunity domains	
Imm69	α + β; W,hGE(h: hydrophobic)	Tox-ABhydrolase3^a^	Actinobacteria, bacteroidetes, firmicutes ^a^, fusobacteria, planctomycetes, α,β.γ,ϵ-proteobacteria,, spirochaetes, verrucomicrobia	Fused to one or more immunity domains such as Imm68, Imm-SUKH, Imm33, Imm-SuFu-family 2, Imm-SuFu, Imm67, Imm66, SP, Imm69 and TPR repeats. Some proteins in firmicutes have up to 10 immunity domains	
Imm70	α + β; Y,W	Tox-REase-10^a^	Acidobacteria, actinobacteria, bacteroidetes, firmicutes^a^, β^a^,γ^a^,ϵ^a^-proteobacteria, spirochaetes^a^, verrucomicrobia		
Imm71	Mostly α; R,F, R	Ntox48^a^	acidobacteria ^a^, β ^a^,γ ^a^ -proteobacteria	Often fused to Imm72	
			***Eukaryotes:*viridiplantae**		
Imm72	All-β; GxxE, WxDxRY, E	Ntox48^a^	acidobacteria ^a^, β ^a^,γ ^a^ -proteobacteria	Often fused to Imm71	
Imm73	All-α; Several hydrophobic residues	Tox-PL-2^a^, Tox-HNH^b^	acidobacteria, actinobacteria^b^, bacteroidetes, cyanobacteria ^a^, firmicutes ^a^, fusobacteria, β,γ,δ ^a^ -proteobacteria, verrucomicrobia	Sometimes found in 2–3 tandem copies in a polypeptide	
Imm74	α + β; G[DE], [DE]	Tox-Arc^a^	bacteroidetes^a^, firmicutes^a^, planctomycetes, α,β,γ^a^,δ -proteobacteria,	Found in heterogeneous polyimmunity loci	

1. Where possible, known or predicted folds are described. The folds are further classified as All-α (composed entirely of α-helices), All-β (composed entirely of β-strands), α + β (Containing α-helices and β-strands) or α/β (comprising repeated α-helix-β-strand units) depending on the arrangement of their structural elements. Individual conserved residues and motifs are separated by commas. Alternative residues are enclosed in square brackets; ‘x’ denotes any residue.

2. Each toxin in column3 that is present in a gene neighborhood along with the corresponding immunity protein in column 1 in the toxin-immunity gene order is marked by a superscript letter, so as to identify the phyletic pattern of this association in column 4.

 In the initial section we present the results of the above analysis from a domain-centric viewpoint by laying out the main conserved domains we identified in toxins (Table 2), immunity proteins (Table 3) and some novel features associated with trafficking (Table 1). In course of discussing the conserved domain families, we describe key features relating to their domain architectures and gene-neighborhoods, and present the relevant functional inferences derived from them. In the following sections we explore the general features of the domain architecture and gene-neighborhood networks, phyletic distribution, relationships between various proteinaceous toxin systems, ecological implications and the evolutionary connections between components of these toxin systems and eukaryotic and viral functional systems.

### **Peptidase domains in polymorphic toxins and related proteins**

Peptidase domains from these systems can be functionally categorized into 1) those that are involved primarily in processing toxin proteins; 2) those that function both in processing and as toxins; 3) those that function mainly as toxins. Autoproteolytic processing by diverse peptidases has been long recognized in classical secreted toxins deployed by pathogenic bacteria against their hosts [49,51,54]. For example, the *Vibrio cholera* RTXA peptide ligase toxin, clostridial glucosyltransferase toxins and certain *Yersinia* toxins are autoproteolytically processed by intrinsic caspase-like thiol peptidase domains, which are induced by small molecules such as GTP and inositol hexakisphosphate in the host cytoplasm [49,52,57]. Similarly, we presented evidence that the HINT autopeptidase domains are likely to be an important player in the autoproteolytic release of several polymorphic toxins (Figure 2A) [17]. In toxins of several pathogens, peptidase domains have also been characterized as bearing the actual toxin activity. Examples include the *Yersinia pestis* YopT papain-like peptidase domain that triggers actin depolymerization in host cells by cleaving the C-termini of Rho GTPases [50] and the *Bacillus anthracis* lethal factor that disrupts signaling cascades by cleaving the N-termini of several MAPK kinase [48]. However, to date peptidase domains have not been systematically characterized in classical polymorphic toxin systems. In polymorphic toxins, peptidases acting in either of the above three functional categories can be distinguished mainly based on their location within the polypeptide. Those involved in autoproteolytic processing are mostly located either at the N-terminus or prior to the C-terminal toxin domain in the multi-domain toxin proteins (Figure 1). The toxin versions invariably occur at the C-termini. Those which might occur at both of these locations can be inferred as functioning as either toxins or processing proteins depending on their position in the polypeptide. In addition to these categories, there are inactive peptidase domains that might serve as peptide-binding modules involved in anchorage and interactions of toxins. We discuss below the previously unrecognized peptidase domains that we identified in polymorphic toxin systems and also discuss their connections to related peptidase domains in other toxin systems (Table 2).

**Figure 3:**
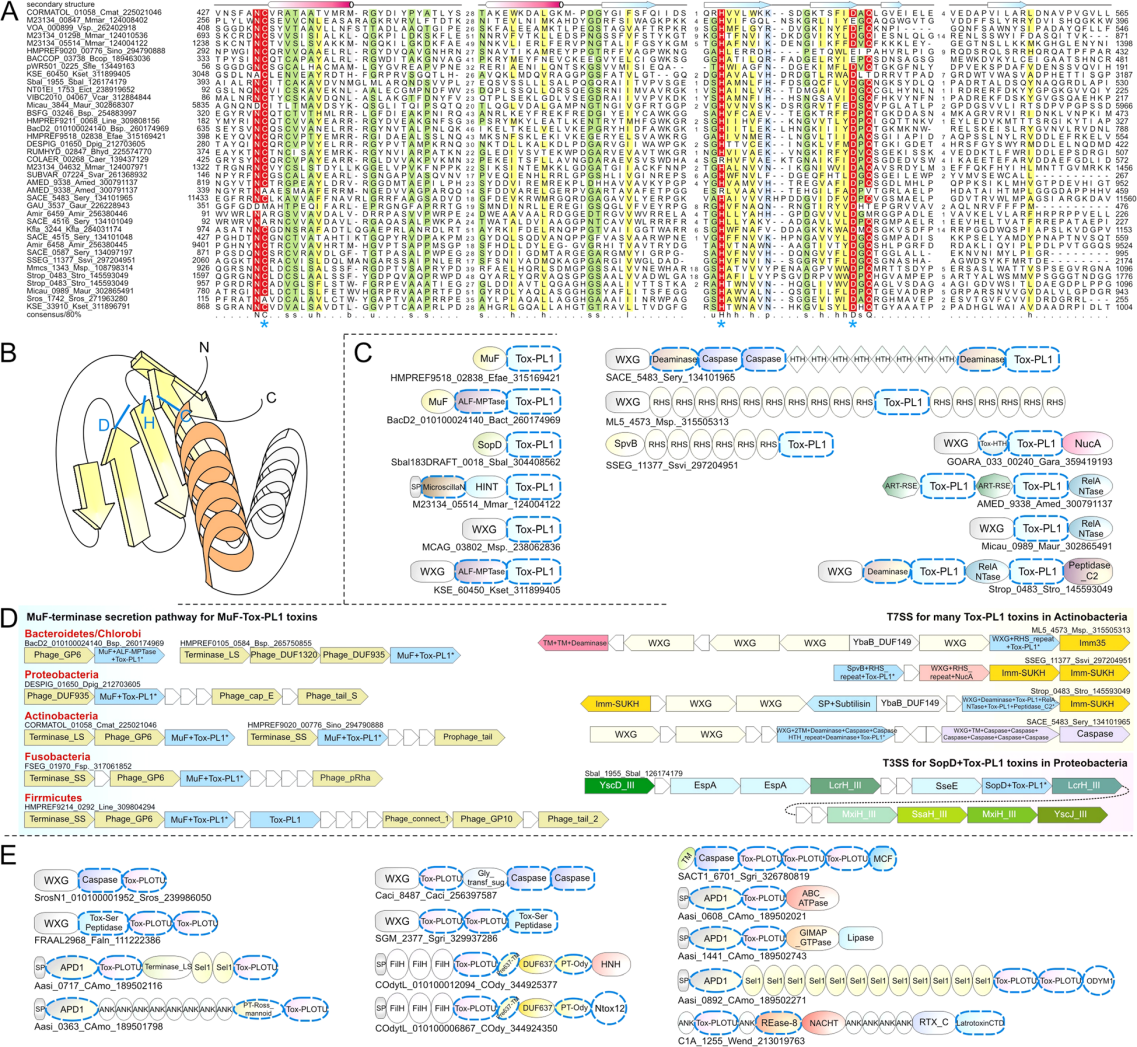
**(A) Multiple sequence alignment, (B) predicted topology diagram, (C) domain architectures, and (D) gene neighborhoods for papain-like peptidase 1 (Tox-PL1) toxins.** (**E**) Domain architectures of OTU papain-like peptidase toxins. The labeling scheme for domain architectures and alignments, and the coloring scheme and consensus abbreviations are as in Figure 3.

### **Domains identified as being primarily auto-processing peptidases**

#### ***ZU5 superfamily domains functions as processing autopeptidase in toxins***

The ZU5 (Zona pellucida 5) domain was first identified as an autoproteolytic domain in the PIDD protein which forms the core of the PIDDosome, a protein complex in animals providing a platform for recognizing molecular patterns that are associated with loss of genomic integrity and genotoxic stress [58]. It is a major player in p53-induced apoptosis and activation of NF-κB pathway in response to DNA damage and its assembly involves multiple autoproteolytic cleavages mediated by its two ZU5 domains [59]. Our structural comparisons with the DALIlite program and sequence profile searches revealed that the ZU5 domain is homologous to the GPS domain involved in autoproteolytic cleavage of the polycystin-1 and certain G-protein-couple receptors [60], and the autoproteolytic domain of the nuclear pore Nup96/98 proteins [61]. All these domains are characterized by the presence of a C-terminal CxH motif which forms their thiol autopeptidase active site (Additional File 1). Accordingly, we include all these domains in the ZU5 superfamily. Our iterative sequence searches identified ZU5 domains in several potential polymorphic toxins: They are typically located at the N-terminus of large proteins with central RHS repeats (Figure 2B). In polymorphic toxins, the ZU5 domain is most frequently associated with the SpvB and β-propeller domains suggesting that it might be functionally coupled to the TcdB/TcaC-like export pathway [42,62]. Its N-terminal location is notably different from the previously observed HINT autopeptidase domains of polymorphic toxins which are instead found at the C-terminus close to the toxin domain [17] (Figure 2B). This suggests that the autoproteolytic activity of the two peptidases have distinct functions – the ZU5 autopeptidase most likely cleaves the toxin at the base of the filamentous structure in order to release it at the cell surface during its extrusion by the TcdB/TcaC system. In contrast, the C-terminally located HINT autopeptidase is likely to be critical for the release of just the toxin domain, probably upon contact with the target cell. In the classical polymorphic toxins ZU5 autopeptidases are found in association with a diverse array of nuclease and peptidase toxin domains (Figure 2B). Related ZU5 domains are also found in several other large bacterial cell surface proteins, which additionally contain diverse adhesion modules and other enzymatic domains, such as glycohydrolases, lipases and phosphodiesterases (Additional File 1). Thus, ZU5 autoproteolytic processing might be a more general feature among bacterial surface proteins that are deployed for the degradation or remodeling of extracellular biopolymers and matrices.

#### ***PrsW peptidase family defines a novel secretion pathway to release C-terminal toxin domains***

The PrsW family of membrane-embedded peptidases is prototyped by the enzyme catalyzing site-1 cleavage of anti-σ^W^ factor RsiW in *Bacillus subtilis*[43]. Most representatives bear eight transmembrane helices and four conserved motifs (Figure 2C), which show distant relationship to several other peptidase families like CPBP and APH-1 [63]. Given that the active site of the PrsW is located within the membrane-spanning helices (Figure 2C), it is likely that they also form a transmembrane conduit for the simultaneous extrusion and processing of the toxin. We first recognized the PrsW domain as being a potential processing peptidase in polymorphic toxins on account of its N-terminal fusion with a novel deaminase toxin domain of the DYW clade (gi: 320532150) [18]. Further analysis revealed that N-terminal PrsW domains are associated with a diverse array of toxin domains, including several distinct versions of the restriction endonuclease superfamily (Figure 2C), mainly in Gram-positive bacteria. These toxin domains are typically connected by a short linker to the core membrane-spanning PrsW domain. However, in certain cases the toxin domain might be connected via a long filamentous structure formed by RHS repeats to the N-terminal PrsW domain (e.g. in a *Streptomyces violaceus* protein with a novel toxin domain (Ntox9; gi: 307326465). Thus, the PrsW domain might be used to autoproteolytically process polymorphic toxins both of the soluble secreted type (one with short linkers) and of the filamentous contact dependent type (with RHS repeats). In archaea (e.g. *Pyrococcus horikoshi* PH0065) and fungi (e.g. *Aspergillus fumigatus*; gi: 146324562), the PrsW peptidase domains are respectively fused at their N-termini to another PrsW-like peptidase (DUF2324 in PFAM), or a ceratoplatanin domain that is found in secreted phytotoxic virulence factors of fungal pathogens [64]. It is conceivable that in these examples the PrsW domain has been recruited for the processing of potential N-terminal toxins that are used against more distantly related organisms or plant hosts. In several bacteria the PrsW domain is fused to intracellular signaling domains such as the PilZ domain which recognizes cyclic diguanylate, cyclic nucleotide binding domains, phosphopeptide-binding FHA domains and Zn-ribbon domains [65] (Additional file 1). These versions can be clearly distinguished both in terms of their sequence relationships and domain architectures from those associated with toxin domains. These are more likely to function as signaling peptidases that cleave proteins in conjunction with signals sensed by the associated domains.

### **Peptidase domains that function both in auto-processing and as toxins**

#### ***Caspase-like peptidases***

As noted above, peptidases of the caspase-like superfamily [66] (also known as “clan CD” [67]) were originally identified as processing peptidases of diverse host-directed toxins (e.g. RTX toxins) of pathogenic bacteria [49,57]. Likewise, some of these domains were identified in certain large bacterial surface proteins where they might function as autoproteolytic processing domains [52]. Other secreted bacterial members of this fold, such as the clostripains have been implicated in proteolytic processing of surface proteins, whereas the gingipains act as virulence factors that cleave host proteins [47]. In this study we obtained evidence based on domain architectures and gene neighborhoods that the caspase-like peptidase domains occur both as potential processing peptidases (typically internal domains) and as toxin domains (the C-terminal-most domain) in polymorphic toxins from bacterial lineages such as bacteroidetes, gammaproteobacteria and actinobacteria (Figure 2D). Architectural analysis clearly shows that the caspase domain toxins might be delivered via the T7SS, PVC-SS, TcdB/TcaC-like export pathway, in addition to the T2SS (Figure 2D). Versions of the caspase-like domain that are likely to function as processing peptidases of polymorphic toxins usually occur just upstream of a distinct C-terminal toxin domain, in a position similar to the HINT autopeptidase domains in other polymorphic toxins (Figure 2A), suggesting that they might similarly aid in the autoproteolytic release of the toxin domain. Architectural analysis suggests that the caspase-like peptidase might be nearly as prevalent as the HINT peptidase in proteolytic processing of polymorphic toxins (Additional File 1). Certain other toxin proteins have an array of repeats of the caspase-like domain upstream of the C-terminal toxin domain (e.g. a protein from *Streptomyces flavogriseus* with ADP-ribosyltransferase and MCF peptidase toxin domains; gi: 357410654; see below) (Figure 2D), suggesting that their processing might involve multiple autoproteolytic events to release multiple cleavage products. Some of the caspase domain repeats in these proteins lack the catalytic residues and might merely play a structural or peptide-binding role.

#### ***Papain-like peptidases***

Papain-like peptidase domains, which constitute the most diverse and widespread superfamily of thiol peptidases, have been previously recorded as the toxin domains of both exotoxins and those delivered into the host cells by various pathogenic bacteria. Examples of the former include the *Streptococcus pyogenes* exotoxin SpeB, while those of the latter include the *Pseudomonas syringae* AvrPphB toxin, which cleaves the plant serine/threonine kinase PBS1, and the *Pasturella multocida* toxin PMT [68-70]. We found evidence for domains belonging to multiple distinct clades of the papain-like superfamily in polymorphic toxin polypeptides.

The first of these, the Tox-PL1 (Tox-papain-like-1) family was recovered as a previously unknown conserved domain in several predicted polymorphic toxins, usually secreted by way of the T7SS (i.e. with N-terminal WxG domains) and TcdB/TcaC-like system (N-terminal SpvB domain) in actinobacteria, and bacteroidetes. Examination of its multiple alignment revealed a conserved NC-H-DxQ signature (Figure 3A), which is reminiscent of the conservation pattern seen in papain-like peptidases [53,71,72]. This relationship was confirmed via profile-profile comparisons with the HHpred program that significantly recovered papain-like peptidases (p = 10^-5^; 95% probability). In a subset of the predicted polymorphic toxins Tox-PL1 is the only catalytic domain, and occurs at the extreme C-terminus of the toxin polypeptide, suggesting that it is the toxin domain (Figure 3C). In other cases it occurs in internal positions in polypeptides bearing a diverse set of toxin domains [18], or in the middle of an array of filament-forming RHS repeats (Figure 3C). In these cases it is likely to function as an auto-processing peptidase that releases associated toxin domains comparable to the HINT and caspase-like peptidases [17]. In *Shewanella* we observed a protein combining a SopD domain [73] with a C-terminal Tox-PL1 domain, which is encoded by a gene embedded within a T3SS operon. Given that *Shewanella* is known to suppress the growth of competing distantly related bacteria and infect eukaryotic hosts [74], it is possible that this protein might be used as a toxin delivered by the T3SS in such conflicts. In diverse bacteria we observed a distinctive architecture of Tox-PL1, wherein it is fused to the MuF domain (Figure 3C), which we had previously characterized as a DNA-packaging protein of bacteriophages utilizing the portal-terminal system [75]. Gene-neighborhood analysis indicated that these are encoded by prophage remnants that also include the terminase, portal protein and capsid protein genes (Figure 3D). Additionally, several of these neighborhoods might encode proteins with previously noted *bona fide* toxin domains that operate on nucleic acids (e.g. the HNH nuclease; Figure 3)[17,18]. Hence, we propose that these gene neighborhoods represent a novel phage-derived secretory mechanism, distinct from the previously identified T6SS and PVC-SS that utilizes a capsid packaging-like mechanism. It is conceivable that in these systems the toxins encoded by associated genes are loaded into a capsid-like structure that is then delivered to target cells. Here, the Tox-PL1 domain might be involved in processing proteins either during the assembly of the secretory structure or the release of toxins into target cells.

**Figure 4:**
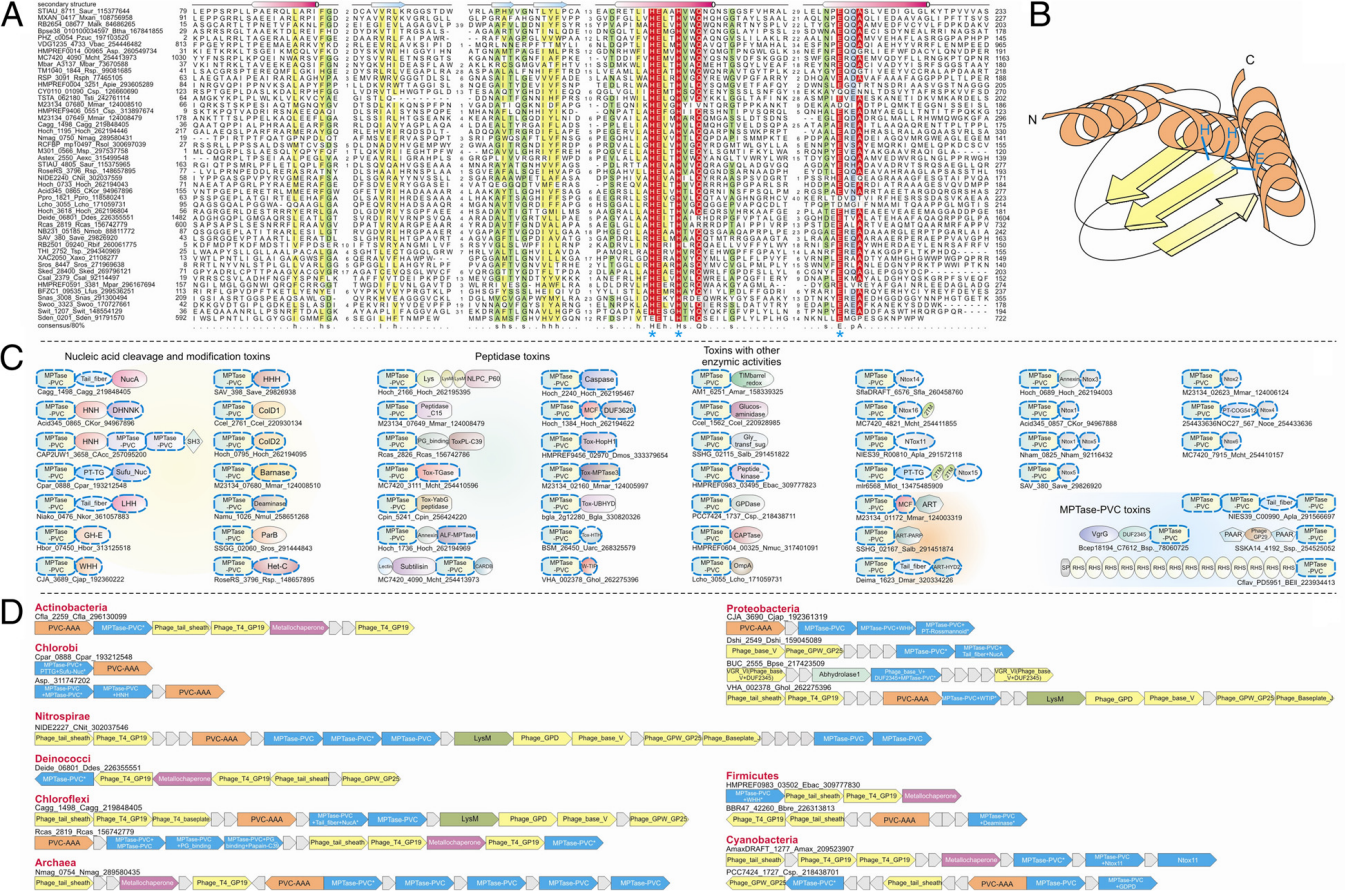
**Features of PVC metallopeptidase toxins: (A) multiple sequence alignment, (B) predicted topology diagram, (C) representative domain architectures, and (D) conserved gene neighborhoods for PVC containing genes across different bacterial lineages and archaea.** In (D), PVC toxins are shown in blue, the AAA + ATPase associated with the PVC system (PVC-AAA) in orange and phage-derived proteins in yellow. Gene neighborhoods are labeled with the corresponding information for the PVC-metallopeptidase containing genes marked with an asterisk. The labeling scheme for domain architectures and alignments, and the coloring scheme and consensus abbreviations are as in Figure 3.

The second major family of papain-like peptidases with potential processing as well as toxin functions are those belonging to the OTU family [53,76] (Figure 3E). These enzymes have been studied mainly in eukaryotes, where they function as deubiquitinating enzymes (DUBs) [77]. We found evidence for a diverse set of OTU peptidase domains in potential polymorphic toxins delivered by the T7SS (with N-terminal WXG domains) in actinobacteria and via T2SS in the *Acanthamoeba* endosymbiont *Odyssella thessalonicensis*[78]. In these bacterial lineages they occupy positions suggestive of both processing and toxin functions (Figure 3E). Additionally, we found related OTU-like peptidases in large proteins resembling polymorphic toxins in several endo- symbiotic/parasitic bacteria of animals and amoebozoans, such as *Amoebophilus, Waddlia* and *Wolbachia.* However, in these organisms their gene-neighborhoods suggest that they are unlikely to be polymorphic toxins used in intra-specific conflicts; rather, they are likely to be used against their host. In several cases, the OTU-like domains of these intracellular bacteria occur at the extreme C-terminus of large proteins with several domains, including repeats forming extended structures such as the Sel1, ankyrin and TPR repeats (Figure 3E). This suggests that they might be deployed similar to the classical polymorphic toxin, but within the host cell. In other proteins from the same group of bacteria they might occur as internal domains accompanied by several other potential toxin domains (Figure 3E), such as GIMAP GTPase, lipase, latroxin-C and Tox-MCF1-SHE (see below). The preponderance of these OTU-like peptidase domains in intracellular bacteria suggests that they might function as toxins that suppress the Ub-dependent anti-pathogen mechanisms of their eukaryotic hosts due to DUB activity [79,80]. Indeed, a comparable role was originally proposed for the OTU-like peptidases in chlamydiae [53,76]. However, their presence in free-living bacteria (e.g. diverse actinobacteria) indicates that a subset of these OTU-like peptidase proteins might function as either as processing-peptidases that autoproteolytically process polypeptides or as conventional toxin domains that cleave proteins in rival cells.

#### ***PVC secretory system-type metallopeptidase domains***

The “*Photorhabdus* virulence cassette” or PVC-SS was originally identified as a prophage-derived secretory system in *Serratia entomophila*, where it delivers toxins that confer a strong anti-feeding activity against the infected grass grub beetle larvae [41] and in *Photorhabdus*, where it extrudes toxins that destroy insect hemocytes by inducing actin condensation [40]. This system is typified by several caudate phage-derived gene products, such as the tail sheath protein and gp19 (these two form the tail tubule), gp25 (forms the baseplate), and a distinct clade of AAA + ATPases that are related to CDC48 [81]. Thus, the PVC-SS parallels the T6SS in being derived from the tails of prophages, but differs from it in terms of the associated AAA + ATPase, which in the case of T6SS is a member of the ClpB clade of AAA + ATPases (ClpV) [39,81,82]. Hence, these two systems represent independent prophage-based innovations that have recruited distinct sets of AAA + ATPases to facilitate recycling of the injection apparatus after it has been deployed. We observed in our recent studies that several toxin domains closely related to those found in polymorphic toxins are secreted via the PVC-SS across most major bacterial lineages and certain euryarchaea (Figure 4). Our preliminary analysis of these toxin proteins secreted via the PVC-SS revealed that they contained a conserved metallopeptidase domain that occurred N-terminal to the toxin domain [17,18]. A more detailed analysis in course of this study indicated that this metallopeptidase domain is a pervasive feature of the PVC-SS and provides an excellent marker to identify novel toxins secreted via this system. Accordingly, we term it the PVC-metallopeptidase (Figure 4). This domain is characterized by a highly conserved HExxHxxQ-E signature and profile-profile comparisons using HHpred recovered several zincin-like metallopeptidases as the best hits (e.g. PDB: 2vqx, 1u4g, 3cqb; p < 10^-5^; >90% probability). A multiple alignment based on these hits suggests that the PVC-metallopeptidase adopts a similar structure with three beta-strands and three alpha helices, with the conserved histidines on the second helix and glutamate on the third helix forming the Zn-dependent active site [83](Figure 4A, B).

**Figure 5:**
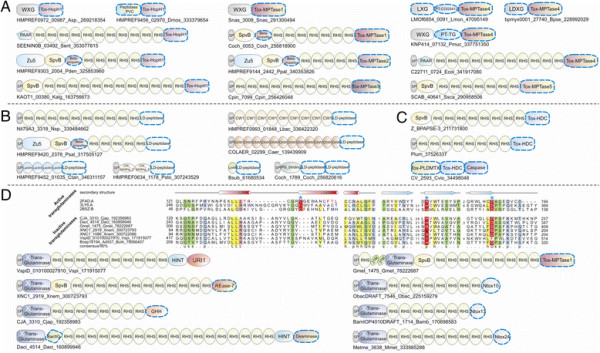
**Representative domain architectures for toxin proteins containing: (A) several distinct metallopeptidase toxin domains such as HopH1 peptidase and Tox-MPTases 1 – 5, (B) LD-peptidase, (C) Tox-HDC domain.** (D) Sequence alignment and domain architectures of inactive transglutaminase-containing toxins. The labeling scheme for domain architectures and alignments, and the coloring scheme and consensus abbreviations are as in Figure 3.

Our analysis of the domain architectures of PVC-metallopeptidase proteins affirmed their general resemblance to the classical polymorphic toxins: the strongly conserved metallopeptidase domain occupied the N-terminal region, followed in each protein by highly variable C-termini, each of which usually corresponded to a different family of toxin domains. Thus, they appear to have evolved through a recombination process comparable to that of the polymorphic toxins, which combined a “constant” N-terminal peptidase with variable C-terminal toxin domains (Figure 4C). This positional polarity of the PVC-metallopeptidase domains with respect to the associated toxin domains resembles that of the HINT, PrsW, caspase-like and papain-like peptidases, indicating that they are likely to act as autoproteolytic domains that release the toxin after or during its export by the PVC-SS [17,18]. The C-terminal toxin domains associated with the PVC metallopeptidases span an extraordinary diversity and include numerous, structurally unrelated nucleases, nucleic acid deaminases, peptidases, pore-forming domains and several other enzymatic domains (Figure 4C). There are multiple toxins with the PVC architecture in several bacteria and archaea (e.g. *Halogeometricum borinquense;* Additional File 1), with a high diversity of C-terminal toxin domains similar to those found in conventional polymorphic toxins. Our analysis also showed that the PVC toxins are not limited to pathogenic or symbiotic bacteria but are abundant in several free-living bacteria (e.g. the cyanobacterium *Microcoleus chthonoplastes* and *Nitrosococcus oceani*) and archaea (e.g. *Halogeometricum borinquense*). This suggests that the PVC-SS toxins are not exclusively used against host but might also be used in inter-bacterial conflicts, just like the T6SS [15,30,39]. However, a notable proportion of the PVC-SS dependent systems, unlike conventional polymorphic toxin systems, lack adjacent genes encoding immunity proteins (Figure 4D). This might imply the activity of PVC toxins is primarily directed against distantly related organisms.

In addition to the above cases, we observed instances where a second PVC-metallopeptidase domain occurred at the extreme C-termini of proteins in a position comparable to the toxin domain (Figure 4C). Consistent with this, domain architecture and gene-neighborhood analysis showed that the PVC-metallopeptidase indeed also occurs as a toxin domain of certain polymorphic toxins, preceded by an array of RHS repeats (e.g. a protein from the verrucomicrobium *Pedosphaera parvula*; gi 223934413; Figure 4C). Similarly, the PVC-metallopeptidase domain might occur as a C-terminal domain fused to a T6SS phage base-plate/tail polypeptide (e.g. *Burkholderia* sp.; gi: 78060725) (Figure 4C). These examples suggest that in addition to its predominant role in autoproteolytically processing PVC toxins, this metallopeptidase might take on the role of a peptidase toxin in several cases.

#### ***The MCF1-SHE domain: A possible novel serine peptidase shared by polymorphic toxins and secreted effectors?***

We initially identified this domain as a conserved region shared by certain predicted polymorphic toxins (e.g. Caci_8529 from the actinobacterium *Catenulispora acidiphila*) and PVC-SS toxins (e.g. Hoch_1384 *Haliangium ochraceum*). Iterative sequence profile searches with the PSI-BLAST program recovered homologous regions in proteins from a diverse group of bacteria and the mimivirus (L389, gi: 311977774) prior to convergence. These proteins include the MCF1 (makes caterpillars floppy) [84] and FitD entomotoxins, respectively from *Photorhabdus luminescens* and *Pseudomonas fluorescens*[85-87]*,* and the phytotoxin of *Pseudomonas syringae* HopT1-1 which is secreted via the T3SS [88,89]. A multiple alignment of this domain revealed that its core comprises of two kinked helices, predicted to form a hairpin (Figure 2E). The predicted kinks in the two helices are respectively associated with a conserved serine and a HxxxE motif and are likely to face each other. Accordingly, we named this domain the MCF1-SHE domain for the first characterized protein that bears it and the conserved triad of residues. While this domain does not resemble any previously known domain, the above catalytic triad suggests that it could potentially function as a novel serine peptidase. In several cases its occurrence at the extreme C-termini of polymorphic toxin proteins points to a potential toxin function for the MCF1-SHE domain (Figure 2E). Consistent with this, it is also found in several secreted proteins of both extracellular pathogens such as *Edwardsiella* and *Xenorhabdus*, and intracellular bacterial and viral pathogens such as *LegionellaCoxiella burnetii* and *Yersinia pseudotuberculosis* and the mimivirus (Figure 2E). In particular it appears to have expanded in legionellae, where up to four distinct MCF1-SHE toxin paralogs might be present per organism. This phyletic pattern suggests that MCF1-SHE proteins might be both toxins in intra-specific conflict and also important effectors that have dispersed through lateral transfer across phylogenetically diverse pathogens. Certain domain architectures of the MCF1-SHE domain are consistent with the predicted peptidase role, although in a different capacity. It often occurs just upstream of several toxin domains, such as the ADP ribosyltransferase domains related to those found in the *Pseudomonas syringae* HopU1 phytotoxin (Figure 2E). In these cases, it could function as a potential processing peptidase that releases the C-terminal toxin. Similarly, in actinobacteria, it is embedded in gigantic proteins (>10,000 amino acids in length) with other peptidase domains such as the anthrax-lethal factor metallopeptidase, caspase-like and OTU domains (e.g. gis: 345002682, 326780819).

### **Other peptidases that function predominantly as toxin domains of polymorphic toxin proteins**

Besides the above discussed domains, we uncovered several other peptidase domains that are clearly predicted to function as toxin domains rather than as processing peptidases on the basis of their domain architectures (Table 2). In addition to classical polymorphic toxin systems and PVC-SS delivered toxins, these peptidase toxin domains are also found in several host-directed effectors of pathogenic bacteria. However, it should be noted that outside of these toxin systems, related peptidase domains might perform other unrelated functions.

#### ***Papain-like peptidases***

Several of the peptidases predicted to function as the toxin domains of classical polymorphic and PVC-SS delivered toxins belong to a number of distinct clades from the papain-like superfamily (Figure 2,4): 1) The NlpC/P60 clade – peptidases of this clade were first recognized as enzymes that cleaved peptide bonds in peptidoglycan and are nearly universally distributed across bacteria and also found in several bacteriophages [71]. We recovered such peptidase toxins in proteins such as Hoch_2166 from the myxobacterium *Haliangium* (gi: 262195395, Figure 4C); by analogy to other members of the NlpC/P60 clade they are predicted to function by degrading cell-walls of target cells. 2) The Tox-transglutaminase domain (Tox-TGase) – In addition to toxins from free-living bacteria, this transglutaminase domain is also found in toxins delivered by different secretory systems of parasitic bacteria, where they appear to be directed against the host cells. In particular, it is the toxin domain of T3SS effectors directed against plants, such as AvrPphE *Pseudomonas syringae* (gi: 30231092) and related effectors of *RalstoniaXanthomonas* and *Acidovorax*, in RTX toxins directed against animal hosts (e.g. *Vibrio caribbenthicus* RtxA; gi: 312885249) and in a novel secreted effector of *Legionella pneumophila* (lpg2408; gi: 52842617). These enzymes might either catalyze a conventional thiol peptidase reaction or act as transglutaminases that mediate crosslinking of proteins via a transglutaminase reaction [53]. Alternatively, they could catalyze polyamination of target glutamine, as has been observed in the case of the *Bordatella pertussis* transglutaminase that modifies the mammalian RhoA GTPase [90]. 3) The Tox-PL-C39 domain – these peptidase domains are related to the C39/ComA-like peptidase domains that cleave the leader-peptides of certain proteins secreted by ABC transporters such as the bacteriocins (Figure 4C) [91,92]. 4) Papain-like peptidases Tox-PL2 and Tox-PL3 – these are novel peptidase domains that we identified in this study and the former is prototyped by the toxin domain of a polymorphic toxin from *Sorangium cellulosum* (gi: 162456110, Figure 2A) and the latter by a polymorphic toxin from *Prevotella sp.* (gi: 260911294, Figure 2B). Thus far, such peptidase domains are not found outside of polymorphic toxin systems and are typified by a C-H-D catalytic triad. 5) We also detected a toxin domain with a papain-like peptidase belonging to the classical ubiquitin C-terminal hydrolase (UBCH/UBHYD) clade associated with the PVC-SS in the plant pathogenic bacterium *Burkholderia gladioli* (gi: 330820326, Figure 4C). Similar UBCH domains are also found in potential toxins secreted by a variety of other bacterial endosymbionts of amoebae such as *Simkania negevensis, Waddlia chondrophila, Amoebophilus asiaticus* and *Protochlamydia amoebophila* and giant nucleocytoplasmic DNA viruses that infect them (Additional File 1). These predicted toxins display no associated immunity proteins suggesting that like the OTU domains of pathogens and endosymbionts, they are likely to function as DUBs that deubiquitinate eukaryotic target proteins [79].

#### ***Metallopeptidases***

Beyond the *toxin versions* (as opposed to autoproteolytic processing versions) of the PVC-metallopeptidase domain described above, we recovered several other distinct clades of the Zincin-like metallopeptidase superfamily that are predicted to function solely as toxin domains in classical polymorphic and PVC-SS toxin proteins. These include: 1) The anthrax lethal factor-like metallopeptidase (ALF-MPTase) domains [48] that are found primarily among PVC-SS delivered toxins (e.g. Hoch_1736 from *Haliangium*; gi: 262194969, Figure4C). 2) The HopH1-like metallopeptidase domain (Figure 5A)—this domain is also found in several plant-directed T3SS-delivered effectors, such as *Pseudomonas syringae* HopH1 (gi: 28867816), and the animal-directed T3SS effectors such as *Citrobacter rodentium* and enteropathogenic and enterohemorrhagic *Escherichia coli* NleD that blocks apoptosis of mammalian cells [93,94]. 3) We also identified five smaller families of previously unknown zincin-like metallopeptidases (Tox-MPTase1-5) that are exclusively found in polymorphic toxins from phylogenetically diverse of bacteria (Figure 5A). In general terms they are similar in size and distantly related to the Wss1-like desumoylating metallopeptidase of eukaryotes [95]. All of these are typically associated with N-terminal RHS repeats and at least in the case of a polymorphic toxin with a Tox-MPTase4 domain from *E.coli*, it might be delivered via the T6SS.

**Figure 6:**
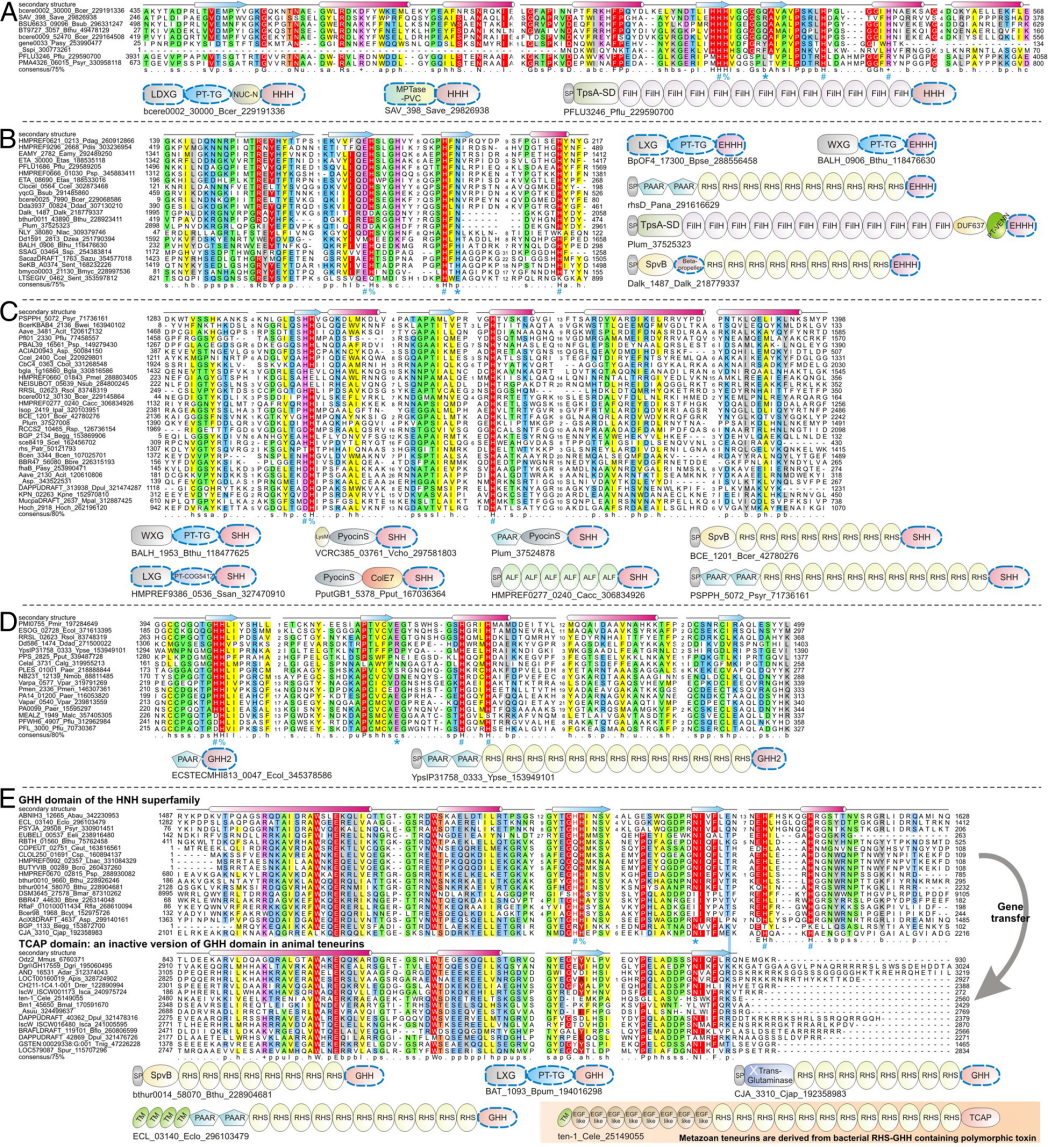
**Sequence alignment and representative domain architectures of novel HNH nuclease families: (A) Tox-HHH, (B) Tox-EHHH, (C) Tox-SHH, (D) Tox-GHH2, and (E) Tox-GHH.** ‘#’ indicates residues involved in metal ion-binding, ‘%’ indicates the conserved histidine which is required for activation of the water molecule for hydrolysis, and ‘*’ indicates polar residues (often asparagine) that are conserved in the HNH fold. The labeling scheme for domain architectures and alignments, and the coloring scheme and consensus abbreviations are as in  3.

#### ***Other miscellaneous peptidases***

Beyond these, we also recovered domains in PVC-SS and polymorphic toxins belonging to the L,d-peptidase, pyroglutamyl-peptidase [96] and YabG peptidase families [97]. Of these, the L,D peptidase domain is a distinct thiol peptidase domain with a β-barrel catalytic domain that is unrelated to the papain-like peptidases (Figure 5B)[98,99]. It has been shown that the classical cell-wall associated LD-peptidase domain catalyzes a transpeptidase reaction that cleaves the peptide bond between L-Lys3-d-Ala4 in peptidoglycan while concomitantly forming a crosslinking peptide bond between the COOH group of L-Lys3 and the NH2 group of the d-isoasparagine linked to the ϵ-NH2 group of Lys3 from an adjacent chain [98]. Cell-wall associated L,d-peptidases are found in most major lineages of bacteria and are likely to play a role in the remodeling of peptidoglycan especially in face of antibiotics that inhibit cross-linking. Polymorphic toxins with L,d-peptidase domain are distinguished from the typical cell-wall associated L,D peptidases by their distinct architecture with RHS repeats and genomic organization with linked immunity proteins. It is likely that the toxin L,D-peptidases act by hydrolyzing L-Lys3 crosslinks with D-amino acids, thereby compromising the integrity of the cell-wall.

The bacteriophage APSE of the endosymbiont *Hamiltonella defensa,* which protects aphids and other sap-feeding insects against parasitoid wasps, encodes several distinct toxins [100,101]. We noted that one of these (APSE305; gi: 211731800) displays an architecture similar to the conventional polymorphic toxins with a potential novel C-terminal toxin domain (Figure 5C). Analysis of this domain revealed that it is widely distributed in several other proteobacteria and is characterized by three motifs respectively bearing a [SGxH] signature, a conserved D or N and an absolutely conserved C (Additional File 1). Secondary structure prediction revealed that this domain is characterized by an α/β fold that is likely to be similar to the Rossmannoid three-layered sandwich adopted by the caspases and the flavodoxin-like fold. The absolutely conserved H, D/N and C are predicted to lie at the ends of the three successive strands of this structure and are likely to comprise the catalytic triad of the peptidase active site. Accordingly we named this domain Tox-HDC and predict that it might function as a thiol peptidase or a transglutaminase. Proteins bearing this predicted toxin domain are particularly common in both intracellular (e.g. *Coxiella burnetii*) and extracellular (e.g. *Xenorhabdus nematophila* and *Photorhabdus luminescens*) pathogens and typically lack associated genes coding for immunity proteins. Thus, these toxins appear to be primarily directed against distantly related targets such as eukaryotes.

In conclusion, at least 23 distinct clades of peptidases belonging to several structurally unrelated superfamilies have been recruited as toxins, and are often shared between polymorphic toxins and host-directed effectors from diverse plant and animal pathogens. This suggests that several of these peptidase domains have evolved considerable substrate flexibility in targeting both eukaryotic and bacterial proteins.

### **Inactive transglutaminase domains in polymorphic toxins**

In course of the current study we observed that several polymorphic toxin proteins with several distinct types of C-terminal toxin domains displayed a N-terminal transglutaminase domain (Figure 5D). However, closer examination of the multiple alignment of these transglutaminase domains revealed that one or more of the conserved residues (a C, H, and D), which constitute the catalytic triad of their papain-like peptidase active site, were lost [53] (Figure 5D). This suggests that they lack peptidase activity. Domain architectural analysis showed that these inactive transglutaminase domains are always located immediately after a N-terminal signal peptide or TM helix and are followed by an array of RHS repeats that constitute the filamentous part of the toxin. Occasionally, they might be adjacent to domains of the immunoglobulin superfamily (the so called “bacterial Ig” type domains; Figure 5D). This position suggests that, unlike the above-described active peptidase domains, these inactive transglutaminases have no role in toxin or processing activity. Instead, they might simply serve in anchoring the toxin on the cell surface by binding peptides.

### **Identification of further toxin domains in polymorphic toxins and related proteins that operate on nucleic acids**

**Figure 7:**
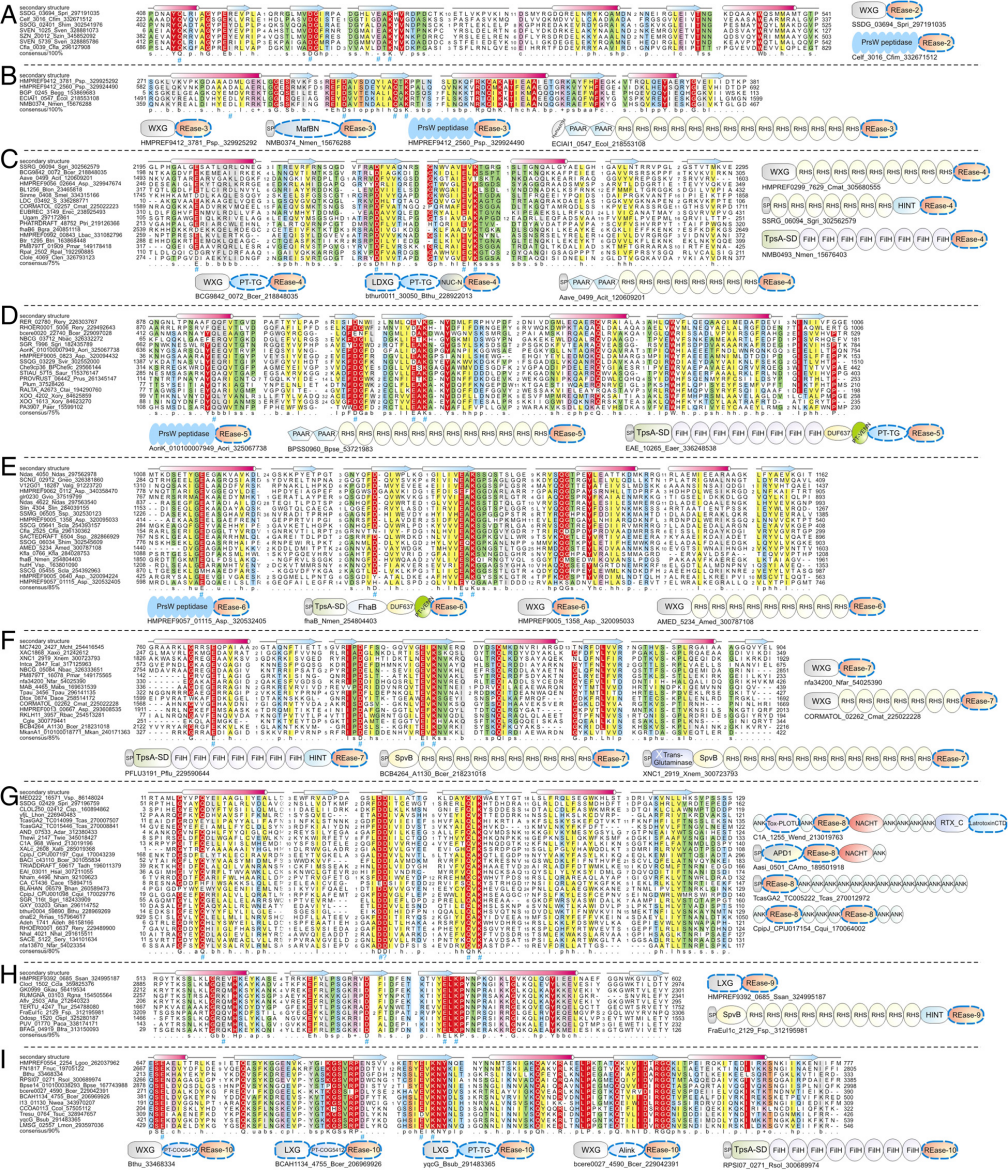
**Sequence alignment and representative domain architectures of novel restriction endonuclease families described in this study: (A) Tox-REase-2, (B) Tox-REase-3, (C) Tox-REase-4, (D) Tox-REase-5, (E) Tox-REase-6, (F) Tox-REase-7, (G) Tox-REase-8, (H) Tox-REase-9, (I) Tox-REase-10.** The labeling scheme for domain architectures and alignments, and the coloring scheme and consensus abbreviations are as in Figure 3.

**Figure 8:**
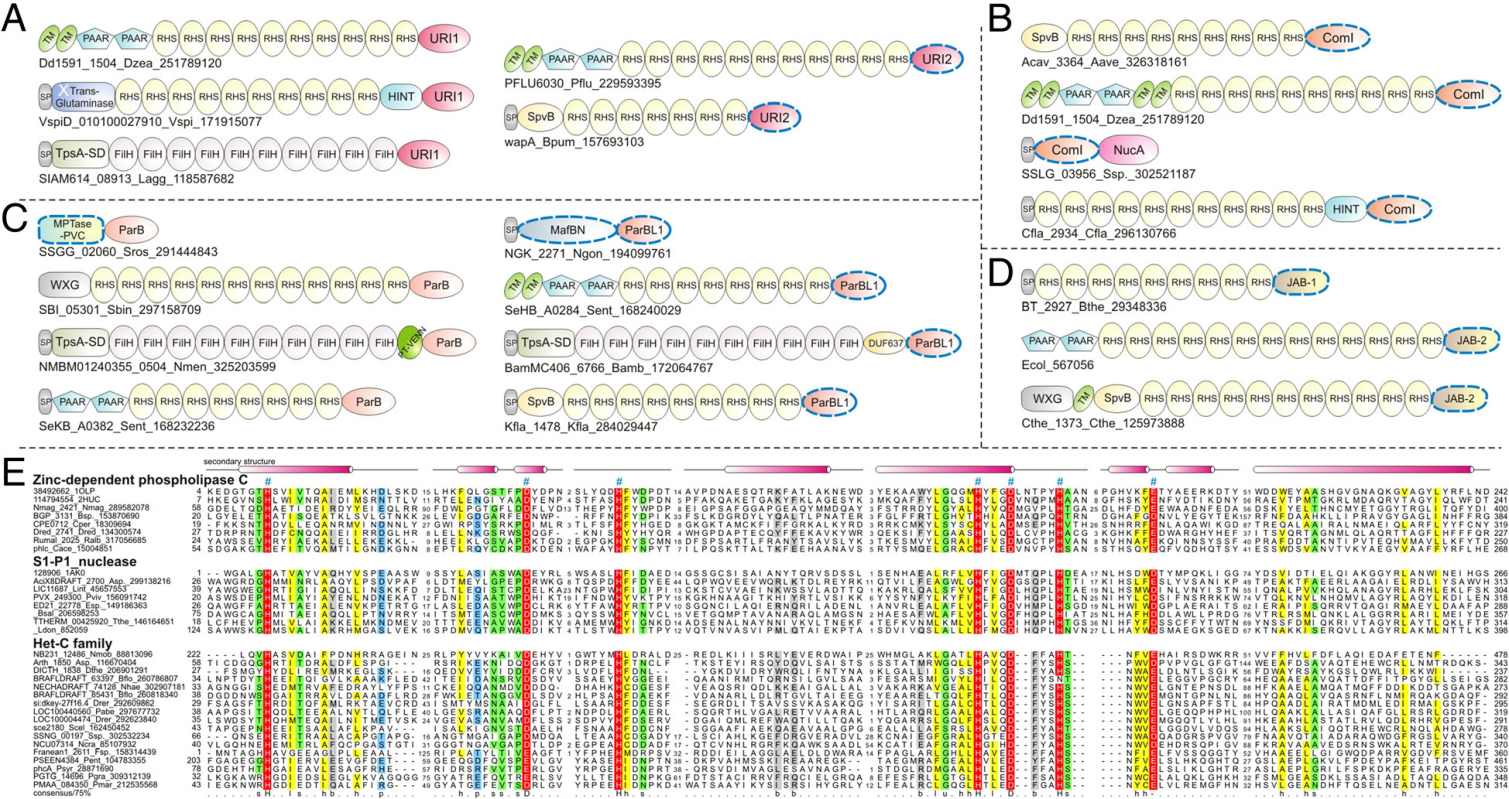
**Representative domain architectures of several nucleic acid-targeting toxin domains: (A) two distinct families of URI nucleases (Tox-URI1 and Tox-URI2), (B) Tox-ComI nuclease, (C) two distinct ParB fold families (Tox-ParB, Tox-ParBL1), (D) two novel JAB families (Tox-JAB-1, Tox-JAB-2).** (**E**) Multiple sequence alignment of the Het-C domain with Zinc-dependent phospholipase C and S1-P1 nuclease, showing their homologous relationship. Conserved catalytic residues are labeled with blue ‘#’. The labeling scheme for domain architectures and alignments, and the coloring scheme and consensus abbreviations are as in Figure 3.

**Figure 9:**
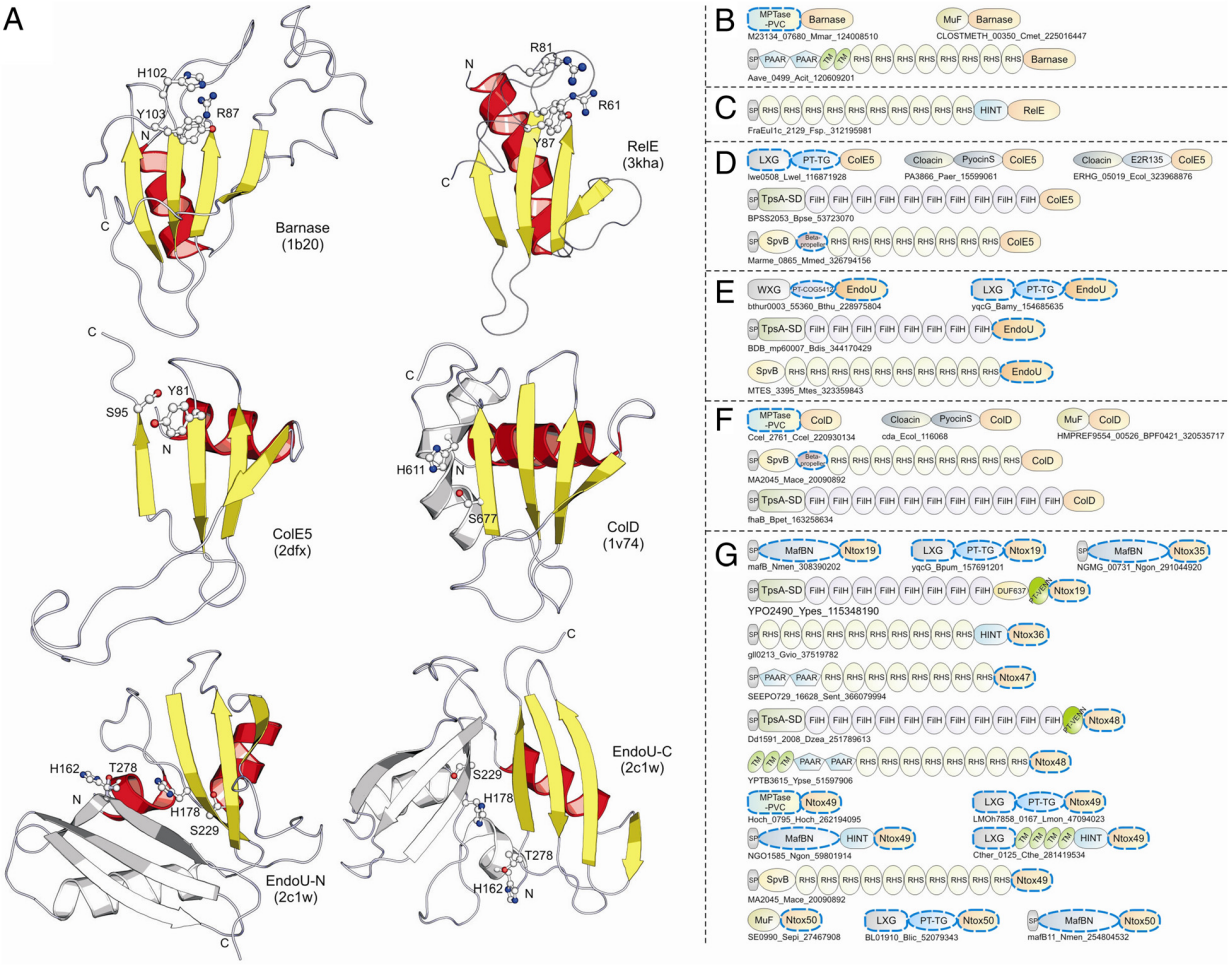
**(A) Shared common core of the BECR fold illustrated with representative structures from Barnase, RelE, ColE5, ColD, and EndoU families.** PDB ids are shown in brackets. All structural cartoons are shown in an approximately similar orientation. The α-helices are colored red, β-sheets yellow and loops gray. The predicted and known active site residues are labeled. Representative domain architectures of polymorphic toxins containing (**B**) Tox-Barnase, (**C**) Tox-RelE, (**D**)Colicin E5 (Tox-ColE5), (**E**) Tox-EndoU, (**F**) Colicin D (Tox-ColD), and (**G**) several other novel toxin domains predicted to contain the BECR fold. The labeling scheme for domain architectures and alignments, and the coloring scheme and consensus abbreviations are as in Figure 3.

In our earlier study we had shown that majority of toxin domains in polymorphic toxin systems operate on nucleic acids – nucleases and base deaminases [17,18]. In this study we were able to further extend the diversity of toxin domains that act on nucleic acids via the discovery of additional nucleases and deaminases that were not previously recognized (Figures 6,7,8,9). We observed that the divalent cation-dependent nucleases among polymorphic toxins are frequently drawn from ancient nuclease folds, namely the HNH/EndoVII, REase and URI endonuclease folds [102-107]. Additionally, we present evidence below that representatives of few other potential cation-dependent enzymatic domains might function as nuclease domains in polymorphic toxins. Interestingly, the PIN domains, which are major divalent cation-dependent nucleases in the toxin-antitoxin systems [22,108], do not appear to be utilized in the polymorphic toxins and related systems. Toxin nucleases that utilize divalent cations can catalyze the direct hydrolysis of the phosphodiester bond and as a result attack both DNA and RNA. However, the metal-independent nucleases can only act as RNases as their endonucleolytic action involves the formation of a cyclic 2’-3’ phosphate that does not require metal-dependent direction of a hydrolytic attack [107]. Such RNases belong to many distinct folds, several of which appear to have emerged only in course of the diversification of toxin domains of polymorphic toxins, bacteriocins and classical toxin-antitoxin systems [17,22,28,107,109,110]. While we were able to unify several of the metal-independent RNases, which were previously considered to be unrelated, into a single monophyletic assemblage, there are still several distinct toxin domains that likely to represent novel metal-independent RNases (see below; novel toxins). This structural diversity of metal-independent RNases and the repeated emergence of several such nuclease domains among different toxin systems suggest that there are some fundamental constraints in the evolutionary innovation of nuclease domains. It appears that the independent emergence of multiple residues for metal-chelation and acid–base catalysis to constitute an active site that can support hydrolytic cleavage of nucleic acids is a far less likely event than the emergence of a metal-independent active site that utilizes the innate reactivity of RNA to facilitate an internal attack with the formation of 2’-3’ cyclic phosphates. We briefly describe below the newly recovered toxin domains that act on nucleic acids.

#### ***Novel toxins with the HNH/EndoVII nuclease domain***

In our earlier studies we found nuclease toxin domains belonging to eight distinct clades of the HNH/EndoVII fold among the polymorphic toxin systems [17,18]. Of these, nucleases belonging to the classical HNH and NucA clades widely occur beyond the polymorphic toxins across diverse sub-cellular systems, such as, DNA repair/recombination, restriction-modification (R-M) and environmental nucleic acid degradation systems [103,106,111]. In contrast, the GH-E, DHNNK, WHH, LHH and AHH domains appear to have arisen in and remained largely restricted to polymorphic toxin systems. The NGO1392 clade appears to have arisen in the bacterial polymorphic toxin systems, but was transferred to eukaryotes where it might have assumed a role in DNA repair [17]. In this study we recovered six more clades of HNH domain nucleases that appear to have primarily diversified among bacterial polymorphic and related PVC-SS-associated toxins. Keeping with the earlier nomenclatural system, we named five of these novel clades on the basis of the conserved motifs that characterized them as the SHH, HHH, GHH, GHH-2 and EHHH clades of HNH domains (Figure 6). The sixth of these is related to the version of the HNH domains found in the restriction enzyme SphI [112] and the animal CIDE (CAD/DFF40) protein involved in nucleolytic DNA fragmentation during apoptosis [113], and is termed HNH-CIDE (Table 2). Architectural analysis indicated that the novel HNH clades occur both as potential diffusible toxins (mainly in Gram-positive bacteria) and as contact-dependent toxins borne at the tip of long filamentous structures (proteobacteria, bacteroidetes, planctomycetes and certain Gram-positive bacteria; Figure 6). Representatives of the SHH clade have been transferred to crustacean (e.g. *Daphnia*; gi: 321474287) and tailed bacteriophages (e.g. *Bacillus* phage SPbeta; gi: 9630134). The former transfer is consistent with occurrence of an effector with a SHH nuclease domain in the eukaryotic endosymbiont, *Simkania* (gi: 338732338).

The CIDE protein was previously known only from metazoans with no known representatives from other eukaryotes; hence, its origin remained mysterious [114]. The identification of the HNH-CIDE toxin domains suggests that this nuclease domain first arose in context of bacterial conflicts and was laterally transferred to animals early in their evolution. In animals, its innate cytotoxic action appears to have been channelized as an effector of apoptosis. Our searches also showed that the C-terminal domain of teneurin and Odd Oz proteins from the animal lineage (metazoans + choanoflagellates) contain an inactive version of a HNH domain belonging to the GHH clade (Figure 6E). While presence of RHS repeats in these proteins related to those in bacterial RHS proteins has been previously recognized [115], the relationship of their C-terminal domain to a specific bacterial toxin domain has not been hitherto reported. Teneurin/Odd Oz proteins function as developmental regulators with a potential role in cell-surface adhesion in diverse processes such as cell migration, neuronal path finding and fasciculation, gonad development, and basement membrane integrity [115-117]. The region of these proteins spanning the inactive GHH nuclease domain has been described as being cleaved off and amidated at the C-terminus in vertebrates to give rise to a peptide with possible neuromodulatory activity [118]. This region in tenurin-2 is also the ligand for latrophilin-1, which is also the receptor for another molecule, latrotoxin, whose origins also lie among the bacterial toxins (see below) [116]. Hence, it is conceivable that the RHS portion of these proteins participates in cellular adhesion, while the cleaved off inactive GHH domain act as a diffusible signal. It would be of interest to investigate if this inactive GHH domain might bind nucleic acids upon being taken up by target cells. Our detection of the GHH domain in the Teneurin/Odd Oz proteins establishes that they have emerged from the single transfer of a specific type of a complete bacterial polymorphic toxin gene followed by its fusion to EGF repeats of animal provenance (Figure 6E).

#### ***Novel restriction endonuclease fold domains in polymorphic toxins***

In our earlier study we had identified toxin domains in polymorphic toxins belonging to a previously uncharacterized clade of the REase fold (REase-1) [17]. Further analysis revealed that there are nine additional, previously unknown clades of the REase fold that are present exclusively as toxin domains of a diverse group of polymorphic toxins (Figure 7; numbered serially REase-2-REase-10). Their domain architectures and gene-neighborhoods indicate that they are secreted by means of the T2SS, T5SS, T7SS, TcdB/TcaC and the PrsW-type peptidase-dependent system in different bacterial lineages. Of these, at least four distinct versions, namely REase-2, REase-3, REase-5 and REase-6 are coupled with a PrsW peptidase, suggesting that a notable diversification of these nucleases appears to have happened in the context of these systems (Figure 7). Many of the REase toxins secreted via the other systems have central RHS repeats (e.g. REase-9; Figure 7). These architectures suggest that REases might function both as diffusible and contact-dependent toxins. Tox-REase-8 is primarily found in the arthropod endosymbiont *Wolbachia* and the *Acanthamoeba* endosymbiont *Amoebophilus* and is usually associated with arrays of ankyrin repeats (Figure 7G). These lack associated genes for immunity proteins and are likely to be deployed against targets in the host cells – this represents the first instance of a REase domain effector being used by endosymbionts of eukaryotes. Representatives of Tox-REase-8 are found in the genomes of arthropods, such as the crustacean *Daphnia*, several mosquitoes, ants and beetles, and the placozoan *Trichoplax*. This suggests that Tox-REase-8 has been repeatedly transferred to diverse animals from their *Wolbachia*-like endosymbionts. Beyond conventional polymorphic toxin systems, REase-9 is also found in a *Parachlamydia* effector (PUV_01770, gi: 338174171) that might target nucleic acids in its host *Acanthamoeba*. All ten clades of REase toxins have an active site that closely conforms to the classical REase active site with a D-[EQ]XK signature in the core strands that constitute the metal-chelating site [103]. The majority of characterized members of this fold act on DNA targets; hence, it is conceivable that these toxins also attack the genome of the target cells through endonucleolytic cleavage.

#### ***URI domain nuclease toxins***

The URI domain was first identified as a conserved metal-dependent endonuclease domain catalyzing the cleavage of the 3′ side of a damaged DNA base during nucleotide excision repair by UvrC, and mediating site-specific insertion of certain introns [102,119]. Similar nuclease domains have also been found in certain REases, such as R. Eco29kI, and the transposase module of Penelope-like non-LTR retroelements [104]. In this work we identified, for the first time, URI domain nucleases in polymorphic toxins that are present in bacteria from most major bacterial lineages (Figure 8A, Table 2) that are usually secreted via T2SS, T5SS, TcdB/TcaC and T6SS. The Tox-URI domains can be divided into two major clades, with the second clade being particularly divergent (Additional File 1). A version of the Tox-URI domain belonging to the first clade has also been transferred to fungi, where it occurs as an intracellular domain fused to an ABC ATPase transporter (e.g. *Neurospora crassa* NCU06946; gi: 164424641; Additional File 1). Given this architecture, it is conceivable that they function in degradation of nucleic acids taken up by these fungi. Interestingly, certain URI domain toxins belonging to the second clade are present in distantly related intracellular symbionts/pathogens of *Acanthamoeba*, such as the *Simkania negevensis* (gi: 338731950), *Odyssella* (gi: 344925485) and *Rickettsia belli* (gi: 91206213). Analysis of the gene-neighborhoods of these toxins suggests that they have adjacent genes encoding immunity proteins (Additional File 1), suggesting that these toxins are likely to be used in intra-conflict rather than being directed against the host. Along with the above-described Otu peptidase toxins from *Odyssella,* these URI domain toxins represent relatively rare examples of polymorphic toxins deployed in intraspecific conflict by endo-symbiotic/parasitic bacteria. Other than the versions from intracellular bacteria, the URI domain toxins are typically associated with filamentous RHS repeats.

All the above metal-dependent nuclease domains are shared by polymorphic toxin systems with R-M systems, but are apparently absent among classical toxin-antitoxin systems [22,28]. However, the versions found in the polymorphic toxins differ from those in classical R-M systems in lacking a complex array of associated DNA-binding domains [120]. Hence, we suspect that the versions of these nuclease domains deployed by the polymorphic toxin systems might have lower target sequence specificity than those deployed in R-M systems. Further, those from the former systems are under selection imposed by the physical interactions with cognate immunity proteins. It appears that these factors might eminently disallow exchange of nuclease domains between polymorphic toxin and R-M systems.

#### ***The competence nuclease (ComI) domain***

This nuclease domain is prototyped by the secreted 17 kDa competence nuclease ComI of *Bacillus subtilis*, which is a major determinant of DNA uptake when the bacterium becomes capable of transformation prior to stationary phase [121]. We recovered related nucleases as toxin domains of polymorphic toxins from actinobacteria (e.g. gi: 296130766 from *Cellulomonas flavigena*) and proteobacteria (e.g. gi: 326318161 from *Acidovorax avenae;* Figure 8B). This domain could not be unified with any previously known fold observed among nucleases. A multiple alignment of this domain showed that it contained a central dyad of two acidic residues (usually a DE motif) followed by a third conserved acidic residue a few positions downstream (Additional File 1). These residues could potentially form a divalent cation-chelating site, suggesting that the ComI nuclease is likely to be the fourth metal-dependent nuclease superfamily among the toxin domains. Interestingly, the *B.subtilis* competence nuclease is physically associated with the 18 kDa product of the adjacent ComJ gene, which acts as its inhibitor – the interplay between the ComI nuclease and its inhibitor ComJ has been suggested to be important for optimal digestion of incoming DNA, so as to facilitate transformation [121]. The structure of this operon with a nuclease followed by its inhibitor is reminiscent of the polymorphic toxin systems with the toxin gene followed by the immunity protein. Consistent with this, ComJ homologs occurs as an immunity protein for polymorphic toxins with the ComI nuclease domain in several proteobacteria. Hence, it is possible that these key components of the *Bacillus* DNA uptake system have evolved from a toxin-immunity gene pair.

#### ***ParB domain toxins***

We recovered several polymorphic toxins with N-terminal filamentous regions formed by RHS or filamentous haemagglutinin repeats and C-terminal ParB toxin domains (Figure 8C). The ParB domain is the subject of much confusion: based on a study, which claimed to demonstrate both endo- and exo- DNase activity in the ParB protein [122], required for maintenance of the plasmid RK2, the domain was labeled as a nuclease domain. However, it should be noted that this study was based on entirely erroneous assumptions that the RK2 ParB domain was related to nucleases such as the staphylococcal nuclease and RuvC [122]. In contrast, other members of the ParB superfamily, such as sulfiredoxin, have been convincingly demonstrated to possess metal-dependent phosphotransferase activity that utilizes ATP to form a phosphoryl ester of sulfinate generated from the active site cysteine of the peroxiredoxins [123]. Through sequence profile searches we were able to demonstrate that DndB is a member of the ParB superfamily. DndB negatively regulates the formation of the unusual DNA phosphorothioate modification, in which the non-bridging oxygen in the phosphodiester linkage of DNA is replaced by a sulfur atom in a sequence-specific manner [124]. Hence, it appears that even this member of the ParB superfamily, comparable to sulfiredoxin might hydrolyze a phoshoryl ester linked to a sulfur center. The convincingly inferred metal-dependent phosphotransfer activity of the ParB superfamily implies that in principle certain representatives might also be able to catalyze nuclease activity through a comparable hydrolysis of a phosphodiester bond. Hence, it is conceivable that, even though the ParB domain was considered a nuclease for the wrong reasons, this activity might be still valid for some representatives of the superfamily. This is also consonant with the earlier recovery of ParB domains in nucleases encoded by certain R-M like systems [103,125]. The predominance of nuclease domains among the toxin domains of polymorphic toxin systems also supports a potential nuclease function for the ParB toxin domains. Examination of the multiple alignment of the ParB domains from polymorphic toxins suggests that they possess a strongly conserved DGHHR motif that is predicted to form part of their highly conserved metal-binding active site (Additional File 1). In addition to the classical ParB toxin domains, we recovered a second large group of toxin domains typified by that found in *Neisseria gonorrhoeae* NGK_2271 (gi: 194099761), which could be united using profile-profile comparisons with the ParB domain (HHpred probability 93%; p = 2x10^-6^ match to 1vz0 *Thermus* ParB). While being rather divergent from the classical ParB domains, they display a motif with a conserved arginine that is equivalent to the DGHHR motif in the former. Additionally, they display a conserved N-terminal serine that is absent in the classical ParB domains. Hence, we termed this distinct family of ParB-related domains as Tox-ParBL1 (Figure 8C). In addition to the bacterial polymorphic toxins, Tox-ParBL1 domains are also found in several eukaryotes such as kinetoplastids, and several metazoans, fungi, plants, stramenopiles and ciliates (Table 2 and Additional File 1). Thus, this example represents an independent acquisition by eukaryotes of a ParB-related domain from the polymorphic toxin systems, distinct from the sulfiredoxins.

#### ***The JAB domain***

We detected two distinct clades of the JAB domain superfamily as the potential toxin domain of several classical polymorphic toxins (Figure 8D). The JAB domain has been previously shown to be a peptidase that specifically targets the C-termini of ubiquitin-like proteins (UBLs) either as a DUB or as a processing enzyme [126-128]. All previously identified prokaryotic JAB domains are intracellular proteins. Most representatives of them are components of systems utilizing UBLs in biosynthetic pathways or protein modification. As these toxin genes are accompanied by immunity proteins they are likely to be used in intraspecific conflict rather than against eukaryotic targets. Hence, the presence of the JAB domain among the toxin modules of classical polymorphic toxins was unexpected, because most of the bacteria in which they are present lack systems with conjugated or processed ubiquitin-like proteins [126]. However, based on contextual information from domain architectural analysis it was recently proposed that a subset of the JAB domains (i.e. those belonging to the RadC clade) are more likely to function as nucleases that cleave DNA, rather than as peptidases [18]. The two clades of JAB domains found among the polymorphic toxins, like RadC, are rather divergent with respect to those that act on UBLs, and do not conserve the residues lining the tunnel that accommodates the UBL tail in the peptidase versions (Additional File 1). This suggests that, as previously proposed for RadC, the toxin JAB domains might function as nucleases rather than as peptidases. Of the two clades Tox-JAB-1 is found in only in the bacteroidetes lineage associated with N-terminal RHS repeats (Figure 8D). Tox-JAB-2 is more widely distributed across proteobacteria, bacteroidetes and few firmicutes which partly overlaps with the “domain of unknown function”, DUF4329 from the PFAM database (Figure 8D). Versions of Tox-JAB-2 are also present in several NCLDVs, such as iridoviruses, mimiviruses and algal viruses, and *Xanthomonas* phages (e.g. phage OP1). These latter versions are secreted proteins and could potentially function as phage-encoded virulence factors.

#### ***The Het-C hydrolase domain***

The Het-C domain was first identified as a major player in the phenomenon of fungal vegetative incompatibility [129], wherein it mediates programmed cell death upon interaction with incompatible hyphae. Subsequently, a version of the Het-C domain encoded by *Pseudomonas syringae* was shown to be required for the infection of fungal hyphae by this bacterium, by exploiting the mechanism of hetero-incompatibility [130]. In our analysis we recovered Het-C domains in systems related to the polymorphic toxins that utilize PVC-SS (e.g. gi: 148657895 from *Roseiflexus;* Figure 4C). Profile-profile comparisons using an alignment of the Het-C domain (Figure 8E) revealed hits with borderline significance (p = .001; 50% probability) to a group of α-helical hydrolases sharing a common a fold, including zinc-dependent phospholipase C [131] and the S1-P1 nucleases [132]. The predicted secondary structure for the Het-C domain was also compatible with the α-helical fold seen in those hydrolases and examination of the multiple alignments revealed that the two possessed a comparable set of conserved active site residues (Figure 8E). This includes four conserved histidines and 3 acidic residues (D/E) suggesting that the Het-C domain possess a metal-dependent active site similar to that seen in the phospholipases and S1-P1-like nucleases. Indeed, secreted versions of this domain with both phospholipase and nuclease activity are known from different bacteria [132]. This suggests that the Het-C domain might also possess either metal-dependent nuclease or phospholipase activity, and that this activity is likely to be critical for the apoptotic and toxin action of this domain in fungi and bacteria.

#### ***Barnase-EndoU-colicin E5/colicin D-RelE like nuclease fold: A large assemblage of metal-independent RNases***

In our earlier study we had recovered the EndoU domain as a metal-independent RNase frequently found in polymorphic toxin systems. We had further shown that the EndoU fold is marked by a potential duplication of a core helix-β-sheet element that constitutes its active site [17]. In another earlier study we had unified the colicin E5 and colicin D RNase domains with the RNase domain of the RelE toxin that is found in classical toxin-antitoxin systems [133]. A comparison showed that the core structural element in EndoU, Colicin E5, colicin D and RelE is a similar strand-β-sheet unit (Figure 9A). Transitive structure-comparison searches using the DALIlite program confirmed that these RNase domains are indeed related as they preferentially recovered each other (with Z > 3.5). Further, these DALIlite searches showed that they could be united with several other metal-independent RNase domains, namely the RNase toxins and other secreted RNases from fungi, such as sarcin, RNaseT and RNase U2, and the bacterial RNases prototyped by barnase (Z > 3.5; Figure 9A; this latter group is described as the microbial RNase fold in the SCOP database [134]). We term the common structural unit shared by all the representatives of the above-unified assemblage the BECR (Barnase-EndoU-Colicin E5/D-RelE) fold. The common structural unit, which constitutes the catalytic domain of the BECR fold RNases contains a N-terminal helical segment that is followed by a sheet formed by 4-stranded meander (Figure 9A). In several cases the 4^th^ strand is followed by an additional short 5^th^ strand that is differentially positioned in various versions of this fold. Furthermore, the location of the active site residues is often comparable across these enzymes and our sequence analysis revealed that many of these RNases (including EndoU, colicin E5/D and some clades of RelE) share a conserved alcoholic residue (S/T) in the 4^th^ strand that contributes to the active site (Figure 9A).

 In addition to the EndoU clade, our sequence comparisons indicated that several of the newly recovered BECR fold toxin domains from polymorphic toxin systems belong to other previously defined clades in this fold, such as barnase, colicin E5, and colicin D clades (Figure 9B-F). While the classical RelE endoRNase domain is common in type-II toxin-antitoxin systems, we observed only a single instance of it being used as a toxin domain in the polymorphic toxins (gi: 357015358 from *Paenibacillus elgii*). However, using secondary structure prediction combined with profile-profile comparisons we also discovered distinct, previously unrecognized clades of RNases displaying the BECR fold (Figure 9G): these include the clades 1) Ntox7 (e.g. y1701, gi: 22125595 from *Yersinia pestis*); 2) Ntox19 (NMW_1482, gi: 254673263 in *Neisseria meningitidis*); 3) Ntox35 (typified by NGMG_00731; gi: 291044920 from *Neisseria gonorrhoeae*); 4) Ntox36 (typified by the toxin domain of gll0213; gi: 37519782 from *Gloeobacter violaceus*); 5) Ntox47 (typified by the toxin of rhs2; gi 366079994 from *Salmonella enterica*); 5) Ntox48 (e.g. gi:251789613 from *Dickeya zeae*); 6) Ntox49 (gi:59801914 in *Neisseria gonorrhoeae*; 7) Ntox50 (gi: 254804532 in *Neisseria meningitidis*). Together with previously characterized clades, these seven novel clades are extensively represented among the toxin domains of classical polymorphic toxins and in some cases related toxins delivered by the PVC-SS (Figures 4 and 9). This observation suggests that the BECR fold has supplied one of the most extensive radiations of RNase toxins, which cuts across mechanistically distinct systems – the polymorphic and related secreted toxins and the classical toxin-antitoxin systems. Examination of the predicted active site residues among the newly characterized clades pointed to each clade acquiring their own unique features. For example, Ntox35 has acquired two conserved N-terminal histidines in addition to the conserved S/T from the C-terminal strand. Ntox50 and Ntox19 instead have a single N-terminal histidine, similar to one observed in several members of the colicin E5/D clade [110], accompanied by a second C-terminal histidine found at the position usually occupied by the conserved S/T of the BECR fold (Additional File 1). The presence of two histidines in the above three clades is reminiscent, though not equivalent in terms of secondary structure context, to those seen in the EndoU clade, suggesting a comparable reaction mechanism in all these versions of the fold. In contrast, Ntox36 lacks any conserved histidine; instead it displays other clade-specific conserved residues; e.g. an asparagine in the N-terminal region. Most of these enzymes, especially those with two conserved histidines are likely to utilize a metal-independent mechanism similar to that observed in RNaseA (see below) [107]. This is supported by the generation of cleavage products with 2’-3’ cyclic phosphate termini in several biochemically characterized members of these RNases (e.g. XendoU). Some members of the EndoU clade have been shown to require Mn^2+^ for effective catalysis of RNA cleavage [135]; however, given that they still produce 2’-3’ cyclic phosphates, it is likely that this metal is required for stabilization of the hypercharged transition state rather than the actual phosphoesterase activity.

Interestingly, we observed that one RNase of the BECR fold related to the colicin E5/D clade is also found consistently associated with the flagellar operon across firmicutes (e.g. gi: 28211324 from *Clostridium tetani*; Additional file 1). It would be of interest to investigate if this RNase is delivered by the flagellar system or alternatively functions to regulate flagellar gene expression as a RNA-processing enzyme. RNases of the Ntox50 clade have also been acquired by bacteriophages such as *Clostridium* phage phiC2 (gi: 134287339) and might be used in conflicts with the host or other phages. Likewise Ntox19 has been acquired by the giant *Acanthamoeba*-infecting mimivirus and is also found in potential effectors secreted by the *Acanthamoeba* endosymbionts *Parachlamydia* and *Odyssella*.

#### ***Novel toxin domains which are likely to function as nucleases***

Our systematic analysis of the polymorphic toxin systems recovered a total 50 distinct novel toxin domains that could not be unified with any previously known domain (Table 2; Additional file 1). Only a small minority of these domains contain at least one experimentally characterized member. Their sequence conservation patterns, together with the preponderance of nucleases among polymorphic toxins, suggest that most of these novel toxin domains are likely to be nucleases. Indeed, their conservation patterns suggest that these novel toxin domains include both potential metal-dependent and independent enzymes (Table 2; Additional file 1). The C-terminal toxin domain of the originally characterized contact-dependent inhibitor protein CdiA from *Escherichia coli* was demonstrated to possess RNase activity [44]. We observed that the *E.coli* CdiA-C domain is widely distributed across polymorphic toxins from diverse bacteria. We also uncovered this domain in the *Photorhabdus* PalA protein, which lacks an associated immunity protein but is encoded in a pathogenicity island adjacent to the Mcf gene whose product is a toxin directed against the caterpillar host [87]. In light of this, it is possible that *E.coli*-CdiA-C domain in PalA might be directed against the host as an accessory toxin. Examination of the *E.coli*-CdiA-C domain shows that it possesses an all β fold that lacks any conserved residues typical of metal-dependent nucleases. Hence, it is likely to be a metal-independent RNase and probably defines a novel structural theme among them.

We uncovered an uncharacterized toxin domain that is found in polymorphic toxin systems from a wide range of bacteria and several potential effectors delivered by endo-symbiotic/parasitic bacteria (e.g. *WolbachiaEhrlichiaOdyssellaRickettsia* and *Legionella*). It is also found at the C-terminus of a group of eukaryotic proteins typified by the plant protein EDA39 and we accordingly call it the Tox-EDA39C domain (Additional File 1). This domain is characterized by two highly conserved histidines respectively in the N- and C-terminal halves of the proteins that are likely to comprise its active site. This conservation pattern is reminiscent of the catalytic residues seen in the RNase A domain [136], and might represent a novel metal-independent RNase that catalyzes a reaction similar to that of RNase A. The presence of this domain in several eukaryotic lineages, such as plants, fungi, oomycetes and *Dictyostelium*, suggests that it might have been acquired by eukaryotes from bacterial endosymbionts and could have been recruited as a potential RNase used in anti-pathogen defense. Ntox43 is typified by the toxin domain of the recently described RhsT from *Pseudomonas aeruginosa*, which has been shown to translocate to the host cytoplasm and mediate an inflammatory response [46]. This toxin, like Tox-EDA39C, has two conserved histidines suggesting that it might also function as a RNase A-like metal-independent nuclease (Additional File 1). Hence, we predict that RhsT is likely to activate the inflammosome via cleavage of specific RNAs. Although proteins with Ntox43 display architectures are similar to classical polymorphic toxins, none of them are associated with adjacent genes for immunity proteins. This suggests that they are likely to be used primarily against eukaryotic hosts. At least four other toxin domains identified by us (Ntox18, Ntox19, Ntox22, Ntox26, Ntox30) are likely to be novel metal-independent endo-RNases that utilize a two histidine-dependent mechanism to catalyze transestrification and formation of a 2’-3’ cyclic phosphate like RNase A (Table 2).

We observed that the RES domain (PFAM: PF08808), whose function was previously unknown, is another toxin domain that is found in polymorphic toxin systems. Interestingly, it is also found in classical toxin-antioxin systems, where it is typically paired with a distinctive antitoxin (previously labeled as a domain of unknown function, DUF2384 in the PFAM database). Hence, we predict that the RES domain is likely to be a novel RNase domain shared by different toxin systems. Examination of the alignment of the RES domain revealed two conserved arginines, a glutamate and a serine – this configuration does not appear likely to support a metal-binding active site; however, these residues are suitable for catalyzing a distinct metal-independent RNase reaction. Ntox24 is characterized by a single conserved histidine, and, like the RES domain, versions of this toxin domain are additionally found in what appear to be novel type-II toxin-antitoxin systems associated with a previously uncharacterized family of antitoxins (e.g. gi: 139439131). The toxin domain from the CdiA protein from *Enterobacter cloacae* (Ntox21) shows universally conserved residues, including a single histidine and two aspartates, but could not be unified with any other known domain. It is conceivable that Ntox24 and Ntox21 act as metal-independent endoRNases comparable to the Colicin E3 nuclease domain [137], which is also found in polymorphic toxin systems (Tox-ColE3)[17]. Our detection of Tox-ColE3 in these systems also helped in emending the proposed active site of these RNases. Based on structural analysis it was previously proposed that the active site of these enzymes corresponds to D55, H58 and E62 in the structure of colicin E3 (PDB:2xfz) [137]. However, our analysis indicated that H58 is not conserved across all members; instead we found that a second histidine, corresponding to H72 in Colicin E3, is conserved throughout the fold. Thus, it is possible that the above types of RNases use a single histidine in conjunction with an acidic residue that initiates cleavage by inducing the 2’OH to attack the phosphodiester backbone of RNA [137]. In contrast, examination of the multiple alignments of the novel toxins revealed potential metal-chelating sites in Ntox29 (conserved histidines and aspartates); hence, it could potentially function as a novel metal-dependent nuclease. For the remaining Ntox domains, while the active site residues could be identified based on conservation, the nature of catalysis remains unclear.

#### ***Deaminases***

**Figure 10:**
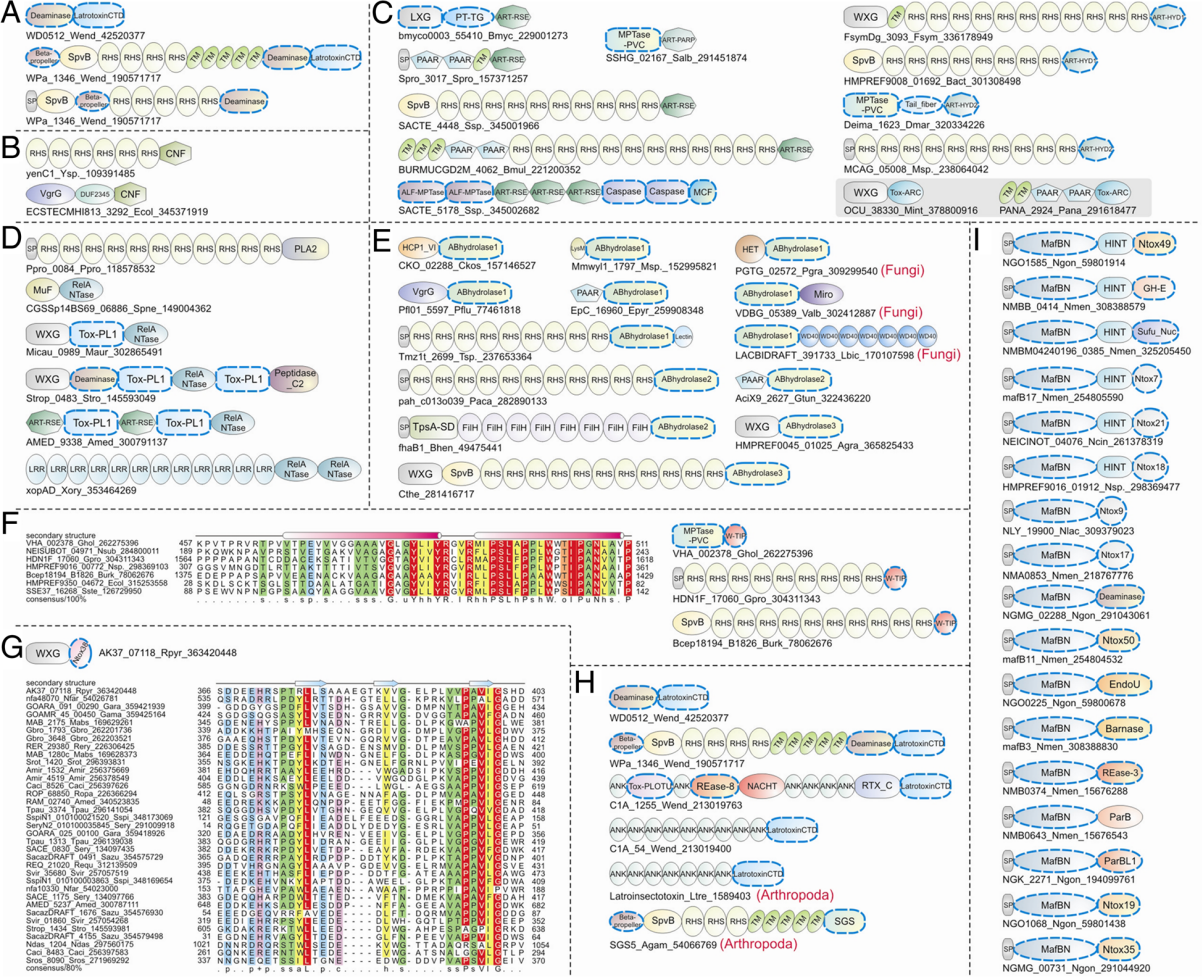
**Domain architectures of polymorphic toxins containing (A) Two novel deaminase families reported in this study, (B) Cytotoxic necrotizing factor (Tox-CNF), (C) several families of ADP-ribosyltransferases (Tox-ART), (D) Phospholipase A2 toxin (Tox-PLA2) and toxin RelE (Tox-RelE), (E) three novel α/β hydrolase families, (F) Tox-W-TIP, (G) Ntox38, (H) novel Latrotoxin C-terminal domain (LatrotoxinCTD), and (I) MafBN secretion related domain.** Also shown in (**F**) and (**G**) are the multiple sequence alignments of the Tox-W-TIP and Ntox38 domains respectively. The labeling scheme for domain architectures and alignments, and the coloring scheme and consensus abbreviations are as in Figure 3.

Other than the nuclease domains, deaminases are the most common toxin domains that operate on nucleic acids in polymorphic toxin systems. As we had extensively characterized the toxin deaminases form these systems in our earlier study [18], we do not consider them in detail here. However, in this study we recovered two additional clades of deaminases that were not previously detected (Figure 10A). The first of these was found in giant proteins with a toxin-like architecture from the alphaproteobacterial endosymbionts of the genus *Wolbachia*, which reside in the cells of two dipterans, namely *Culex* (gi: 190571717; WPa_1346) and *Drosophila* (gi: 42520377, WD0512). These proteins contain two toxins at their C-termini, of which the Latrotoxin-CTD (see below) is the terminal toxin and the deaminase N-terminal to it (Figure 10). An examination of their gene neighborhoods revealed that they lacked accompanying genes encoding immunity proteins. Hence, it appears that these proteins, while resembling the classical polymorphic toxins, are primarily directed against host nucleic acids. The deaminase domains from these proteins are extremely divergent, but structure prediction based on a multiple alignment with a comprehensive set of deaminase domains showed that they belong to the “Helix-4 division” of the deaminase superfamily in which the 5intervening 4^th^ helix of the core domain causes strands 4 and 5 to be parallel to each other [18]. Thus, they are united with other deaminases of this division such as TadA/Tad2, ADAR/TAD1 and the AID/APOBEC-like deaminases. However, unlike most members of this division the newly characterized deaminase domains have a CXE signature in their first active site motif, as opposed to usual HXE seen in this division (Additional File 1). These newly detected versions add to the earlier identified deaminases belonging to the Helix-4 division among host-directed toxins of alphaproteobacterial endo-symbionts/parasites, such as those from the *Wolbachia* endosymbiont of the lepidopteran *Cadre cautella* and from the *Orientia* and *Rickettsia* species infecting diverse eukaryotes[18]. This suggests that modification of nucleic acids by these fast-evolving deaminase toxins related to the eukaryotic AID/APOBEC-like proteins might be a widely used strategy by endosymbionts to alter host physiology. In particular, the presence of such highly divergent versions of deaminases in *Wolbachia* infecting diverse arthropods hints that they could be attractive candidates for mediating failure of paternal chromosome condensation via its mutagenic action [138]. The second novel clade of deaminases are toxin domains of classical polymorphic toxins from proteobacteria and actinobacteria, which might be delivered via diverse secretory mechanisms such the T2SS, T5SS, T6SS, T7SS and the TcdB/TcaC system (prototyped by gi: 162451789, sce3516 from *Sorangium cellulosum*; Figure 10A and Additional File 1). These deaminases usually have a HAE signature in their first active site motif but belong to the “C-terminal hairpin” division of the deaminase superfamily, which is characterized by a C-terminal β-hairpin following the 3^rd^-helix of the conserved core. Given their predominance in free-living bacteria, unlike the former deaminases, they are likely to be deployed in intraspecific conflict rather than against eukaryotic hosts.

### **Other catalytic toxin domains in polymorphic toxin systems**

Other than the peptidase and nucleic acid cleaving or modifying toxins we uncovered several other less frequent catalytic domains that function as toxins in polymorphic and related secreted toxin systems (Table 2). These display a wide range of activities and are likely to elicit their cytotoxic activity by attacking several independent aspects of cellular function. We briefly outline these toxin domains and their possible modes of action.

#### ***Domains catalyzing modifications of proteins***

The previously characterized DOC domain, which has been observed in several host-directed effectors (e.g. *Xanthomonas* AvrAC), is found in several polymorphic toxins [22,139,140] (Figure 2D). This is a protein-modifying toxin domain, which transfers AMP or UMP from nucleotide triphosphates to serines or threonines on target proteins [139,140]. Another toxin domain that we recovered in polymorphic-toxin- related systems utilizing the PVC-SS showed a specific relationship to the serine/threonine kinase domain found in lantibiotic synthetases [141] (Figure 4C). The “eukaryote-type” kinase domain in the lantibiotic synthetases phosphorylates serine/threonine residues in the lantibiotic precursors to prime them for the generation of the thioether linkages. Lantibiotic synthetase-type kinase domains have been shown to possess generic S/T kinase activity [142], suggesting that the toxin versions might carry out their action by phosphorylation of proteins on S/T residues in target cells. A comparable protein-modifying toxin domain (gi: 291451822, from *Streptomyces albus,* Figure 4C) is a glycosyltransferase, related to the *Clostridium difficile* toxin B, which has been shown to glycosylate the hydroxyl group of threonine 37 in the switch I region of the small GTPase RhoA [143]. Given the conservation of the Mg2 + −binding DXD signature, which is critical for catalyzing the transfer of UDP-linked sugars, in versions of this domain found in toxin polypeptides detected in our study, it is likely that it functions in a similar fashion by glycosylating serines or threonines in specific proteins in target cells. In addition to its presence in classical polymorphic toxins with N-terminal RHS repeats and PVC-SS delivered toxins, we observed that related glycosyltransferase domains are also found in effector proteins delivered by various intracellular bacteria. In the endoparasite *Legionella pneumophila* it is present in a toxin delivered via the T4SS (gi: 307610704) and in the aphid endosymbiont *Hamiltonella defensa* (gi: 238899322) it might be deployed as a toxin against the parasitoid wasps that attack the host aphids [144]. A distinct protein-modifying toxin domain is typified by the CNF domain of the uropathogenic *E. coli* cytotoxic necrotizing factors 1 and 2 and the dermonecrotic toxins of *Bordetella*. These domains display a 4-layered sandwich fold, with an active site histidine and cysteine, and catalyze the deamidation or transglutamination of a specific active site glutamine in the small GTPases, like RhoA, Rac and CDC42, in the cells of their eukaryotic host [140]. We recovered CNF domains in potential proteobacterial polymorphic toxins (Figure 10B) with N-terminal filamentous regions (*Yersinia* sp. yenC1, gi: 109391485) as well as those fused to phage-tail VgrG domains of the T6SS (e.g. 345371919 from *E.coli*).

We also encountered several distinct clades of ADP ribosyltransferases (ARTs) among the toxin domains of polymorphic and related toxin systems (Figure 10C) [145]. The ART superfamily can be divided into two major clades depending on the conservation pattern of the three key active site residues associated with the three conserved motifs, respectively from the N-terminus, central region and C-terminus of the domain. These are the R-S-E clade and the H-Y-E clade, named after their respective conserved active site residues [146-148]. Protein-modifying ART domains have been extensively studied in the context of the host-directed toxins of diverse bacteria. Members from the R-S-E clade include the cholera toxin, which modifies a specific arginine in a mammalian Gα subunit, the *Bordetella pertussis* toxin which modifies cysteine, the *Clostridium botulinum* C3 toxin that modifies asparagine, and the *Photobacterium luminescence* toxin which modifies glutamine in target proteins [145,148]. The H-Y-E clade includes the *Corynebacterium* diphtheria, *Vibrio* cholix and *Pseudomonas aeruginosa* exotoxin A toxins, which modify diphthamide in the translation GTPase eEF-2, and the polyADP ribsosyl transferases (PARP/PARTs) [146,149,150]. We found multiple R-S-E clade ART domains in classical polymorphic toxin systems. One type of R-S-E clade ART toxin domains, observed in certain polymorphic toxins (e.g. gi: 221200352 from *Burkholderia multivorans*), is also seen in the T3SS effectors of *Pseudomonas syringae,* namely hopO1-1/2/3, a *Legionella pneumophila* T4SS effector (gi: 307611385), a novel *Protochlamydia amoebophila* effector (pc1346; gi: 46446980), and *Pseudomonas aeruginosa* exoT (gi: 347302423). Such ART toxin domains are also found in a remarkable group of giant proteins from actinobacteria (e.g. 345002682; *Streptomyces* sp.; Figure 10), which combine several toxin domains such as two anthrax lethal factor-like metallopeptidase, two caspase, three ART and one MCF1-SHE domains (Figure 10). A second distinct type of R-S-E clade ART domains, which is found in similar actinobacterial toxins (e.g., gi: 320008023 from *Streptomyces flavogriseus*), is closely related to the lepidopteran ARTs, such as pierisin, which ADP-ribosylates the N2 atom of guanine in DNA to induce apoptosis and the insecticidal toxin of *Bacillus sphaericus*[151]. Interestingly, the close relationship of the lepidopteran pierisin-like ARTs to the bacterial insecticidal toxins suggests that they were probably a late lateral transfer into these insects from a bacterial symbiont or parasite, followed by their reuse as an apoptotic effector. In this study we found novel toxins of the H-Y-E clade from actinobacteria, which are closely related to the eukaryotic PARPs (Tox-ART-PARP), and are associated with the PVC-SS from (e.g. gi: 291451874 from *Streptomyces albus*). We also identified related toxin domain among the toxins secreted by the intracellular pathogen *Legionella drancourtii* (e.g. LDG_5757; gi: 374260808). Additionally, we also found three distinct families of toxin ARTs belonging to the H-Y-E clade. The first of these is an extremely divergent version, which is typified by a protein with an architecture similar to a classical polymorphic toxin from *Shewanella baltica* (gi: 152999126), but without associated immunity proteins and might be directed against eukaryotic hosts. The two other families (Tox-ART-HYD1 and 2 prototyped by gi: 336178949 and gi: 238064042 respectively) are widely distributed in free-living bacteria and are associated with distinct immunity proteins suggesting that they might be mainly deployed in intraspecific conflict like the classical polymorphic toxins. Nevertheless, versions of Tox-ART-HYD2 appear to have been transferred to several eukaryotes such as fungi and choanoflagellates (e.g. gi: 331216471 from *Puccinia graminis*). The above observations suggest that the use of ARTs to modify proteins, and in some cases DNA, appears to be yet another strategy that is common to effectors deployed in both intra-bacterial and bacterio-eukaryotic conflicts.

#### ***Lipid-modifying toxin domains***

Three distinct lipid-modifying enzymes are represented among the toxin domains of classical polymorphic toxins and related PVC-SS-delivered toxins. Two of these namely the glycerophosphoryldiester phosphodiesterase (GPDase, gi: 218438711 from *Cyanothece*) and the CDP-alcohol phosphatidyltransferase (CAPTase, gi: 317401091 from *Neisseria mucosa*) domains are found exclusively in PVC-SS toxins (Figure 4C). In contrast, phospholipase A2 (PLA2) is found in classical polymorphic toxins with filamentous N-terminal regions (e.g. gi: 118578532 from *Pelobacter propionicus*), which might be secreted via different mechanisms, including the T6SS (Figure 10D). Of these the GPDase can catalyze the hydrolysis of glycerophospholipid head groups by releasing alcohols linked to glycerol 3-phosphate via a phosphodiester linkage [152]. On the other hand, phospholipase A2 can hydrolyze lipids by releasing of one of the fatty acid tails from glycerol 3-phosphate [153]. Closely related homologs of the Tox-phospholipase A2 domains (Tox-PLA2) are also found in secreted proteins from fungi and oomycetes (Table 2, Additional File 1). More generally, phospholipase A2 domains are also found in animal toxins from reptilian venom and from mammalian immune systems [152], suggesting that the use of this domain as a toxin is a prevalent strategy throughout evolution. Intriguingly, members of the CAPTase superfamily are membrane-embedded enzymes catalyzing the reverse reaction (lipid synthesis) using cytidine-diphosphate-linked alcohols as substrates, e.g. phosphatidylserine, phosphatidylcholine, phosphatidylglycerolphosphate, phosphatidylinositol and cardiolipin synthetases [154]. It is conceivable that a novel lipid synthesized by this toxin domain creates discontinuities in lipid bilayers, as has been observed with cardiolipin [155]. Thus, all three of these enzymes could potentially mediate their cytotoxicity by damaging the cell membrane of target cells, either through hydrolysis of lipids or disruption of the bilayer.

A toxin domain was uncovered in several classical polymorphic toxins (e.g. Tmz1t_2699 from *Thauera* sp.; gi: 237653364) that partly overlapped with a “domain of unknown function” (DUF2235 in the PFAM database). Sequence profile searches with the PSI-BLAST program recovered significant hits to α/β hydrolases (e = 10^-5^-10^-7^; iteration 3 in a search initiated with the domain from the above *Thauera* protein). While α/β hydrolase superfamily encompasses hydrolases with several distinct activities, such as lipases, peptidases and thioesterases, profile-profile comparisons with the HHpred program suggested that these α/β hydrolases (Tox-ABhydrolase-1) are closest to lipases (e.g. the recovery of triacylglycerol lipases; PDB: 1tgl). In most cases this α/β hydrolase domain is either found fused to N-terminal phage base-plate modules (e.g. gi: 77461818 from *Pseudomonas fluorescens*) or encoded by a gene adjacent to a gene coding for such modules (Figure 10E). This suggests that Tox-ABhydrolase-1 might be a toxin that is mainly delivered via T6SS. These α/β hydrolase domains also appear to have been transferred to fungi prior to the divergence of the ascomycetes and the basidiomycetes and are present in most fungal lineages. We recovered two more distinct, previously uncharacterized α/β hydrolase families that are potential toxin domains that are associated with numerous classical polymorphic toxins (Tox-ABhydrolase-2 and 3, Figure 10E). Profile-profile searches with ABhydrolase-3 recovers the lipases (e.g. pdb: 1lgy; p = 10^-12^; probability 95%) as the best hit to the exclusion of other ABhydrolases. Hence, it is conceivable that Tox-ABhydrolase-1 and Tox-ABhydrolase-3 are further toxins that might disrupt cell-membranes of target cells via their action on lipids. ABhydrolase-2 is primarily present in proteobacteria and has also been transferred to ascomycete fungi. It is also found in the endosymbiont *Parachlamydia amoebophilus* independently of an immunity protein and might be deployed against host molecules. However, Tox-ABhydrolase-2 did not show any specific relationship to previously characterized lipases. Given, that the ABhydrolase superfamily includes hydrolases with a very diverse array of activities, it is not clear if Tox-ABhydrolase-2 might also act on lipids or target some other cellular component.

#### ***Carbohydrate-related toxin domains***

We detected two enzymatic domains, which are predicted to act on carbohydrate substrates, as toxin domains of polymorphic and PVC-SS-delivered toxins. The first of these belongs to a superfamily of glycohydrolases, typified by bacterial proteins, such as FlgJ and the N-acetylmuramoyl-L-alanine amidase (gi: 220928985 from *Clostridium cellulolyticum*), which cleave the glycopeptide linkages in peptidoglycan or endo-glycosidic linkages in oligosaccharides [156,157]. Hence, it is likely that these toxin domains act by hydrolyzing linkages in the peptidoglycan of the target cells. These might be compared to the recently described amidase toxins from *Pseudomonas aeruginosa* that are believed to act on peptidoglycan [15]. The second toxin domain in this group is an oxidoreductase with a TIM barrel fold catalytic domain (gi: 158339325 from *Acaryochloris marina*) [158]. Within this superfamily, the toxin domains are most closely related to the aldo-keto reductases, such as 2,5-didehydrogluconate reductase, suggesting that they are likely to act on sugar substrates. However, the exact mode of action of this toxin remains unclear – it could either act on carbohydrates in the peptidoglycan or within target cells.

#### ***Toxin domains related to nucleotide signaling***

The RelA/SpoT-like toxin domain is found in classical polymorphic toxins from Gram-positive bacteria delivered by the ESX/T7SS (e.g. 302865491; Micau_0989 from *Micromonospora aurantiaca*; Figure 10D). A related toxin domain is also found in the T3SS-delivered effectors directed against plant hosts by several plant pathogens, such as *Xanthomonas* (e.g. gi: 353464269; the XopAD effector), *Ralstonia solanacearum* and *Pseudomonas syringae*. These proteins typically contain two copies of the RelA/SpoT domain. Further, in several bacteria (e.g. gi: 149004362 from *Streptococcus pneumoniae* and gi: 254362874 from *Mannheimia haemolytica*) the RelA/SpoT toxin domain is found fused to the MuF domain of prophages and is thereby predicted to be delivered via this distinct phage-derived system. The RelA/SpoT is a nucleotide-binding domain related to the DNA polymerase β-type nucleotidyltransferase fold [159] that synthesizes the alarmone (p)ppGpp [160]. It has been observed that high levels of (p)ppGpp in non-starvation conditions rapidly inhibits growth and protein synthesis [160]. Hence, it is conceivable that this toxin acts as an unregulated alarmone synthetase in target cells to shut down their protein synthesis. Its widespread presence in several phylogenetically distant plant pathogens is consistent with the presence of a (p)ppGpp-dependent signaling pathway in plants, similar to that seen in bacteria [160]. In light of this, it appears likely that the MuF-fused versions found in the animal pathogens such as *Streptococcus pneumoniae* and *Mannheimia haemolytica* might be deployed in intra-bacterial conflict similar to the classical polymorphic toxins, rather than against the animal hosts.

Another distinct nucleotide generating enzymatic domain, which we found in several polymorphic toxins from several major bacterial lineages (Figure 10C), is the ADP-ribosyl cyclase (Tox-ARC) domain. These toxins are coupled to various delivery systems including T5SS, T6SS and T7SS. This domain has previously only been characterized in animals and generates two distinct metabolites, namely cyclic ADP ribose (cADPr) and nicotinic acid adenine dinucleotide phosphate (NAADP), respectively from NAD and NADP [161]. The former two nucleotides have been shown to function as potent inducers of calcium influx via the ryanodine receptors [162]. At the same time by channeling NAD it can also affect protein deacylation by Sirtuins and other processes requiring NAD [163]. Given that polymorphic toxins with Tox-ARC domains occur in free-living bacteria, and are typically coupled with the genes for the immunity protein Imm74, it is likely that they are used in intra-specific conflict rather than against eukaryotes. Their mode of action in the bacterial context is not entirely clear – it is possible that they deplete NAD or NADP and interfere with various metabolic processes dependent on them. Alternatively, the cADPr or NAADP generated by them could have toxin consequences for the target cell, for example by interfering with NAD-utilizing process such as RNA metabolism or DNA ligation. The bacterial Tox-ARC domains show considerably more sequence diversity than the eukaryotic counterparts and appear to have been the progenitors of two independent sets of eukaryotic representatives in animals and fungi respectively.

### **Non-catalytic toxins: Pore-forming and peptidoglycan-binding domains**

Several classical polymorphic and PVC-SS delivered toxin proteins display unusual C-terminal predicted toxin domains that do not show any indications of being enzymes. Further analysis of these predicted toxin domains suggested that they are likely to operate via non-catalytic mechanisms. One of these, which is thus far restricted to proteobacteria is the W-TIP domain that was named after a conserved tryptophan and TIP tripeptide motif (Figure 10F). This small toxin domain is highly hydrophobic in composition and is predicted to form two membrane spanning-helices. The first of these helices bears two absolutely conserved positively charged residues (RxxR signature), while the second bears the W-TIP motif. These features suggest that the W-TIP toxin domain might effect its cytoxicity by forming a transmembrane pore similar to pore-forming toxins from diverse organisms [164,165]. Several PVC-SS delivered toxins also display a single annexin domain (Figure 4C); however, this domain is unlikely to be a stand-alone toxin domain as it is always followed by a further C-terminal *bona fide* enzymatic toxin domain (e.g. the anthrax lethal factor-like metallopeptidase and Ntox3 domains; Figure 4C). The eukaryotic annexins typically contain four tandem annexin domains and bind both phospholipids, such as phosphatidylinositol (4,5)-bisphosphate (Annexin A2) and phosphatidylserine (Annexin A5), or components of lipid rafts such as cholesterol (Annexin A2) [166]. The eukaryotic annexins also have the unusual capability of apparently traversing cell membranes despite lacking signal peptides. Hence, it is conceivable that the annexin domains in bacterial toxins act as accessory domains that aid in the breaching of target cell membranes to facilitate the delivery of the C-terminal toxin domain.

One of the most enigmatic toxins is Ntox38 (Figure 10G), which is currently restricted to actinobacteria, and might be found in several paralogous copies per genome (e.g. 7 copies in *Actinosynnema mirum* and 9 copies in *Saccharopolyspora spinosa*). This toxin domain is usually linked to a N-terminal WXG domain by a low-complexity glycine-rich linker, suggesting that it is secreted via the T7SS. This is further supported by the frequent presence in their gene neighborhoods of a gene encoding a subtlisin-like serine peptidase associated with processing of proteins secreted via the T7SS [126]. The Ntox38 domain is just 33–43 residues in length and is predicted to adopt a simple three-stranded fold (Figure 10G). Its size and lack of potential conserved catalytic residues suggest that it is unlikely to be an enzymatic domain. It shows several, conserved hydrophobic residues and an invariant C-terminal PXhhG signature (where h is a hydrophobic residue). It is one of the few toxin domains whose mode of action remains rather elusive, but is likely to involve a physical interaction with a key cellular component rather than catalytic modification. It shows a strong association with a single immunity protein, Imm56.

We uncovered an unusual toxin domain at the C-termini of giant toxin proteins from arthropod alphaproteobacterial and gammaproteobacterial endosymbionts such as *Wolbachia* and *Rickettsiella grylli* (Figure 10H). Homologous domains are also found at the C-termini of the latrotoxins (latrotoxin-CTD) of the black widow spider (*Latrodectus* species) [167]. The latrotoxins also display other architectural similarities with the above bacterial toxins in sharing N-terminal ankyrin repeats. Interestingly, the latrotoxins are not secreted in a conventional fashion, but released upon disintegration of the producing cell [167]. Upon release the latrotoxin-CTD is proteolytically cleaved off to form the mature latrotoxin [168]. Given that the latrotoxin-CTD is shared by distantly related bacterial endosymbionts, which colonize a wide range of arthropods, it appears likely that the spider latrotoxins were acquired via lateral transfer from a bacterial endosymbiont. The latrotoxin-CTD is characterized by a conserved, hydrophobic helix; hence, it is possible that it associates with the membrane and might facilitate disintegration of the producing cells in spiders. Bacterial toxins with latrotoxin-CTDs do not display any neighboring immunity protein genes; hence, it is likely that they are primarily used against the eukaryotic hosts. In this regard, it is interesting to note that the salivary gland proteins of mosquitoes have been suggested as being laterally transferred from *Wolbachia*[169,170]. We found that such proteins are more widely distributed across arthropods (e.g. the crustacean *Daphnia pulex*), and that they are related to endosymbiont toxin proteins, such as those reported above. However, in place of a C-terminal toxin domain they contain a conserved domain termed the SGS domain (for salivary gland secreted protein), which is not found in any bacterial toxin, but only in arthropods (Figure 10H, Additional File 1). Thus, it appears that following lateral transfer of a bacterial toxin protein, the toxin domain was displaced by an arthropod-specific domain. Hence, the latrotoxin and SGS proteins could represent different examples of toxins of endosymbiotic bacteria being coopted for arthropod-specific functions.

Several toxins delivered via the PVC-SS displayed a putative toxin domain belonging to the OmpA superfamily of peptidoglycan-binding domains [171-173] (e.g. gi: 171059731 from *Leptothrix cholodnii*; Figure 4C). While several toxin polypeptides contain domains that might facilitate extracellular adhesion, including peptidoglycan-binding domains such a PGB1 and the LysM domains, the OmpA domain, unlike those, always occurred at the extreme C-terminus. This supports the inference that in these cases the OmpA domain might have a toxin function. The OmpA domains have been shown to anchor porins and the T6SS to the peptidoglycan [172-174]. Given that OmpA domains can bind peptide precursors for peptidoglycan biosynthesis [172], it is possible that such toxin domains might act by interfering with peptidoglycan synthesis through binding of such peptides.

### **Lineage-specific expansion of N-terminal domains in toxin proteins: Novel secretion/anchoring mechanisms?**

The N-terminal domains of the full length polymorphic toxins are usually good predictors of their trafficking pathways because they contain domains that are specific to a given secretory pathway (Table 1). We found another interesting feature in the N-terminal regions of certain polymorphic toxins and related proteins from endo-symbionts/parasites secreted via the T2SS, which is thus far restricted to a few bacteria. This feature is characterized by the presence of lineage-specific domains that occurs downstream of a N-terminal signal peptide in full-length toxins from certain organisms. The best example of this is provided by the MAFB group of polymorphic toxins found in *Neisseria* species (Figure 10I). Here all the full-length toxin proteins display a globular domain, the MAFB-N domain (Additional file 1; overlapping but not identical to the model defined as the domain of unknown function DUF1020 in the PFAM database), just after their signal peptide. Across different full length toxins the MAFB-N domain is highly conserved, which is in sharp contrast to the C-terminal polymorphism in their toxin domains (Figure 10I). Furthermore, though the MAFB-N domain is strongly conserved in the genus *Neisseria*, the MAFB-N domain is not found outside of it. In terms of operonic organization, all full-length genes encoding MAFB-N type polymorphic toxins are accompanied by an upstream gene which encodes MAFA, a secreted protein with a lipobox, indicating that it is a lipid anchored surface protein [175]. Like the MAFB domain, the MAFA domain is restricted to *Neisseria* and shows no polymorphism. This suggests that the conserved MAFB domain of these polymorphic toxins is likely to interact with the surface-anchored MAFA protein, thereby anchoring them to the cell surface. This hinted that certain lineage-specific N-terminal domains might serve as a surface anchor for toxins. A comparable situation was observed in a group of seven polymorphic toxins in *Microscilla marina*, which are typified by a conserved N-terminal domain upstream of their signal peptides (Microscilla-N). This conserved globular domain is currently not observed outside of this species and might again play a specific anchoring function for these polymorphic toxins. It is also conceivable that homotypic interaction between these “constant” N-terminal domains help spatial clustering of different toxins on the cell surface.

Like *Microscilla*, yet another member of the bacteroidetes clade, i.e. the *Acanthamoeba* endosymbiont *Amoebophilus asiaticus* displays a variety of effectors, which are predicted to be directed against its eukaryotic host, that are united by shared conserved N-terminal domains. We were able to identify two distinct types of such N-terminal domains that occur immediately downstream of a signal peptide and a lipobox, that we termed Amoebo philus-prodomain 1 (APD1) and 2 (APD2) respectively (Additional File 1). The presence of the lipobox prior to APD1 and APD2 suggests that these effectors do not diffuse into the host cytoplasm, but are likely to be anchored on the surface of endosymbiont. The proteins bearing the APD1 and APD2 domains show highly conserved N-termini but extremely polymorphic C-termini, with several distinct effector domains – thus, they appear to represent a mechanistic principle similar to the MAFB-N and *Microscilla* toxin N-terminal domains. However, unlike the classical polymorphic toxins, where the C-terminal domains are serially variable due to displacement by alternative toxin domain cassettes, the *Amoebophilus* effectors with diverse C-termini are likely to be deployed in parallel at the same time [79]. Among the variable C-terminal domains of these effectors are several domains shared with the toxin domains of polymorphic toxin systems, such as: 1) papain-like peptidases of the Otu family; 2) lipase-like α/β hydrolases; 3) The EDA39C-like nucleases. Additionally, these effectors also display diverse C-terminal domains that are specifically related to the ubiquitin system, such as the F-box and U-box subunits of ubiquitin E3 ligases, SMT4/Ulp1-like desumoylating and UBCH-like deubquitinating peptidases, and other regulatory modules such as the GIMAP-type GTPase domains, STAND NTPase domains, SecA-like helicase-related domains and SbcC-like ATPase domains[79,176,177]. This suggests that over and beyond typical toxin-like effectors, the *Amoebophilus* effectors also interface with the host via a wide range of catalytic activities that are typically not encountered in the polymorphic toxin systems. Indeed, the deployment of effectors interacting with the eukaryotic Ub-system is a common strategy used by several endo-symbiotic/parasitic bacteria as well as exoparasitic bacteria that deliver effectors via different secretory systems [80]. On the other hand deployment of STAND NTPases and GIMAP-type GTPases is a strategy limited to endo-symbiotic/parasitic forms. Nevertheless, the presence of the lineage-specific APD1 and APD2 domains suggests that, as in the case of the polymorphic toxin systems, these N-terminal domains might mediate surface anchoring or homotypic interactions that allow clustering of effectors to certain locations on the cell surface. Given the lineage-specific nature of this feature, it might turn out to be more widespread upon more careful analysis.

### **Immunity proteins**

**Figure 11:**
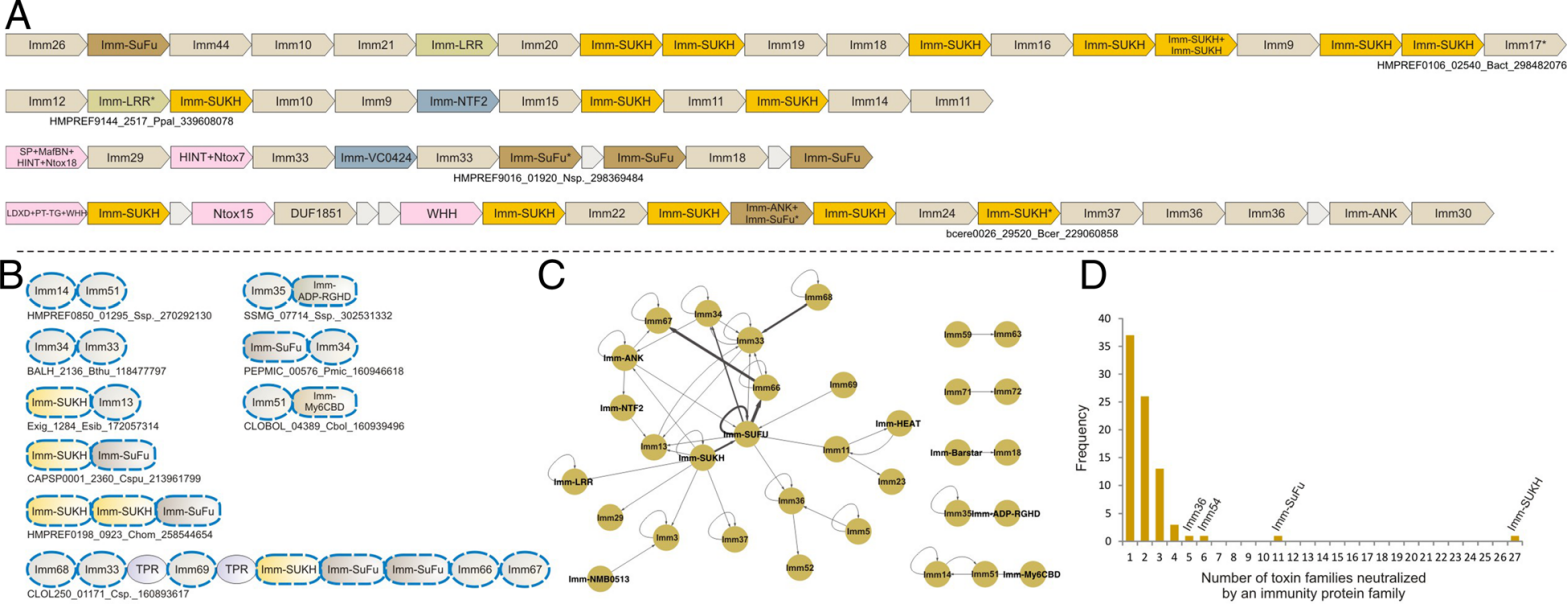
**(A) Representative examples of poly-immunity gene loci.** (**B**) Representative examples of poly-immunity proteins. (**C**) Domain architecture network of immunity domains in poly-immunity proteins. (**D**) Frequency of immunity protein families that neutralize a given number of toxin domains.

Our earlier studies had revealed that two major immunity protein superfamilies, namely SUKH and SuFu, dominate the polymorphic toxin systems [17]. The current study further corroborated this observation – systematic comparisons revealed that members of the SUKH superfamily act as immunity proteins across the greatest mechanistic and structural range of toxins. They were found as immunity proteins for toxin domains belonging to 18 distinct families of nucleases displaying eight distinct folds, three families of deaminases, DOC-like protein AMP/UMPylating enzymes, TIM-barrel aldo-keto reductase, two types of α/β hydrolases and two mechanistically distinct peptidases (Table 3). We extended the diversity of the SuFu superfamily by identifying a second, previously unknown clade of SuFu domains (Table 3, Additional File 1). These domains are extremely divergent with respect to the classical SuFu domain but could be unified with them by means of profile-profile comparisons (p = 10^-6^; probability 86% for matching the classical SuFu superfamily profile). Together, the two clades of SuFu domains are immunity proteins for toxins with six families of nuclease domains of the HNH/EndoVII fold, the ParB domain, Ntox7 nuclease domain, peptidase domains belonging to two unrelated folds and the glycerophosphodiester phosphodiesterase domain. Thus, the extended SuFu superfamily is only next to the SUKH superfamily in terms of the mechanistic and structural range of toxins that it can neutralize (Table 3). A key point to note is that these two superfamilies of immunity proteins work across toxins, which utilize entirely unrelated biochemical mechanisms and target very distinct types of macromolecules (RNA, DNA, proteins, lipids and carbohydrates; Table 3). This observation supports our earlier proposal that the SUKH and the SuFu superfamilies primarily function by being able to bind diverse target proteins by means of sequence variability in their respective versatile binding interfaces [17]. Thus, in a sense they parallel the use of certain highly variable but versatile binding interfaces found in domains from eukaryotic antigen receptors such as the leucine rich repeats and the immunoglobulin domain [178]. Beyond the SUKH and SuFu superfamilies, we recovered over 85 different superfamilies of immunity proteins associated with polymorphic toxin systems (Table 3). In contrast to the SUKH and the SuFu superfamilies, majority of these are specific to only one or a few types of toxin domains (Table3, Figure 11). For example, the Imm-barstar is specifically associated with toxins containing the barnase-like nuclease domain, and Imm39 with URI domain nucleases across practically all major bacterial lineages. Likewise, Imm35 is specifically associated only with the papain-like peptide Tox-PL1, suggesting that it functions specifically as a peptidase inhibitor. The strong association with a single family of toxin domains indicates that several of the immunity proteins have evolved to counter only a single type of toxin. Unlike the versatile immunity proteins, these tend to strongly conserve an interface that facilitates a very specific interaction with their cognate type of toxin. Thus, we observe opposing evolutionary trajectories among the immunity proteins: few versatile immunity proteins are selected for sequence diversification at binding interface to cope with a structurally diverse range of the toxin domains, whereas a large number of immunity proteins are selected to retain the ability to specifically interact with a single type of toxin domain across a wide phylogenetic range.

All but few of the currently identified immunity proteins are cytoplasmic globular proteins and typically do not show relationships to any known enzymatic domains. This implies that they primarily act in the cytoplasm by directly binding to the toxin domains. Two immunity proteins (Imm-CdiI and Imm17) show a comparable architecture in being comprised of two TM helices. Unlike the other immunity proteins these might act by preventing uptake of the toxin at the cell membrane. Likewise, a subset of the immunity proteins associated with the L,D peptidase, which is predicted to function on the cell-surface, are secreted or TM proteins, consistent with the localization of the active toxin. Imm65, which shows a strict association with Tox-JAB-1 is also exceptional in being the only immunity protein in our collection that appears to be a lipoprotein anchored via its N-terminal lipobox. Imm-ARG is also exceptional in that it is the only currently known enzymatic immunity protein – it contains a catalytically active ADP-ribosylglycohydrolase domain (ARG)[148]. Given that it strictly associates with toxin ARTs of the R-S-E clade, it is likely that Imm-ARG neutralizes these toxins by reversing the ADP-ribosylation catalyzed by them.

Secondary structure analysis indicates that on the whole the majority of immunity proteins are α + β domains (64%) followed by all-α domains (25%). Interestingly, while there are over 50 different types of immunity proteins, with α + β domains being preponderant, only a few of them belong to previously characterized superfamilies of domains mediating protein-protein interactions in other sub-cellular contexts. Among these are Imm-NTF2 and Imm-NTF2-2 (NTF2 fold domain), Imm-MyosinCBD (related to the cargo-binding domain of the type VI myosins of animals), Imm-LRR (leucine-rich repeats), Imm-Ank (Ankyrin repeats) and Imm-HEAT (HEAT repeats), which display domains that are widely used in protein-protein interactions across several cellular systems (Table 3). However, unlike the SUKH or SuFu superfamilies, none of these immunity proteins with versions of previously characterized interaction domains are widely used across different toxin types in the polymorphic toxin systems. Some otherwise common protein-protein interaction domains used in other biological systems, such as the immunoglobulin or β-propeller domains, have not yet been found among immunity proteins. This suggests that, rather than widely coopting common protein-protein interaction domains that are prominent in other sub-cellular systems, the polymorphic toxin systems have selected for their own unique set of proteins specializing in protein-protein interactions (Table 3). In the case of the SUKH and the SuFu superfamilies, evidence from gene neighborhoods and phyletic patterns suggests that they primarily function in the context of the polymorphic toxin systems and were on several occasions secondarily adapted for other protein-protein interaction functions, especially in eukaryotes and viruses [17]. Interestingly, most immunity protein superfamilies are entirely absent in archaea (Table 3). This is consistent with the general paucity of classical polymorphic toxin systems in most archaea; though haloarchaea display functionally related PVC-SS delivered toxin systems (See below for further discussion). These observations also indicate that the polymorphic toxin systems have provided a unique niche in bacteria for the innovation of a great variety of domains mediating distinctive protein-protein interactions, majority of which are not utilized elsewhere. Nevertheless, at least 13 distinct types of immunity proteins have been transferred on different occasions to eukaryotes (Table 3). While some of these transfers to eukaryotes are ancient, the majority of these transfers are to fungi and diverse amoeboid eukaryotes which share micro-environments with bacteria. It would be of interest to investigate if these have been adapted for eukaryote-specific functions as observed in the case of the SUKH and SuFu superfamilies [17]. In conclusion, we suggest that a systematic structural investigation of the toxin-immunity protein interactions might offer a unique opportunity to study the evolutionary constraints acting on protein-protein interaction interfaces.

#### ***Polyimmunity loci and polyimmunity proteins***

Our earlier analysis had indicated the presence of tandem arrays of genes encoding several distinct paralogous immunity proteins of the SUKH superfamily, many of which are often only distantly related to each other [17]. We term these “polyimmunity loci”. Such polyimmunity loci were suggested to function as potential backups that allow organisms to survive not only their own toxins but also neutralize a range of toxins that might be delivered by non-kin strains that are present in the environment [17]. Further, they might provide reservoirs of immunity proteins that allow an organism to potentially “cover” any new toxin it might evolve or acquire through lateral transfer. In this study we systematically identified several new polyimmunity loci and further extended this concept to include homogeneous and heterogeneous polyimmunity loci (Figure 11A): The homogeneous polyimmunity loci are defined as those which are dominated by a single type of immunity protein e.g. several tandem paralogs of the SUKH superfamily [18]. The most frequently found homogeneous polyimmunity loci are those containing tandem SUKH superfamily genes. In addition, Imm6, Imm11 Imm28, Imm33, Imm36 and Imm 41 also form prominent homogeneous polyimmunity loci (Additional File 1). In contrast, the heterogeneous polyimmunity loci contain a wide range of structurally unrelated immunity proteins. For example, a heterogeneous polyimmunity locus from *Bacteroides* sp. D22 encodes 19 different immunity proteins belonging to 13 distinct superfamilies, of which the SUKH superfamily alone is represented by 6 distinct versions in this locus (Figure 11A). As such these polyimmunity loci represent a unique type of prokaryotic gene cluster – they differ from other large prokaryotic gene clusters in concentrating genes that are effectively functionally equivalent in a certain sense rather than encoding multiple subunits of a protein complex (e.g. ribosomal or CRISPR operons) or enzymes catalyzing successive steps of a complex pathway (e.g. the antibiotic and siderphore biosynthetic operons) [179,180].

Examination of both polyimmunity loci reveals several interesting features (Figure 11A and Additional File 1): 1) The immunity genes in a polyimmunity locus are never interrupted by intervening toxin genes or toxin cassettes. Thus, they are distinct from regular polymorphic toxin loci, which typically display arrays of toxins or toxin cassettes, often with an adjacent immunity protein. 2) The intergenic distance between two immunity genes in a polyimmunity locus is typically small and they are arranged in the same orientation. This implies that they might be transcribed into a single polycistronic message, from which multiple immunity proteins are synthesized at once. This appears to distinguish them from the immunity proteins located within a regular polymorphic toxin locus in which only the complete toxin gene and its adjacent immunity protein are expressed [181]. 3) The polyimmunity loci show considerable differences in terms of the number and type of included immunity genes, even between strains of the same species (Figure 11A). 4) In several cases the polyimmunity loci are adjacent to genes encoding recombinases, such as the XerC/D recombinase (Additional File 1). It is conceivable that the recombination mediated by these adjacent elements might play a role in accumulation of immunity genes at polyimmunity loci. 5) Usually organisms possess only a single polyimmunity locus. A minority of the organisms possess more than one polyimmunity locus (~13% of the organisms with polyimmunity loci). 6) Extended polyimmunity loci (i.e. those with four or more tandem immunity genes) are not found in all bacterial lineages – thus far, they are only found in certain lineages of proteobacteria, bacteroidetes, firmicutes and actinobacteria. This suggests that extended polyimmunity loci are probably selected for only in certain ecological settings (see below). Some of the above features indeed suggest that these loci are probably under selection to provide a preemptive defensive backup against a constantly changing profile of deployed toxins in context of frequent, recurrent organismal conflicts (see below for further details).

 Comparable to the polyimmunity loci, are the polyimmunity proteins, which combine multiple immunity protein domains into a single polypeptide (Figure 11B). Thus, they may be viewed as polyvalent immunity proteins that have the ability to neutralize more than one toxin simultaneously or serially. We first observed such polyimmunity proteins in the SUKH superfamily, wherein the same protein contains multiple tandem repeats of the SUKH domain [17]. Similarly, we observed that the SUKH domain might also be fused to SuFu and Imm33 (DUF2185) domains indicating that there are polyimmunity proteins, which combine structurally unrelated immunity domains in the same polypeptide. A systematic search for polyimmunity proteins revealed several additional architectures (Figure 11B). Some of the largest polyimmunity proteins combine up to 10 distinct immunity domains in a single polypeptide (e.g., gi: 160893617 from *Clostridium* sp. L2-50; Figure 11B). Given its prevalence as an immunity domain, not surprisingly, the SUKH domain is a common denominator in several of these polyimmunity proteins – it is combined with at least 8 structurally unrelated immunity domains in different polypeptides (Figure 11C). The other prominent domains in polyimmunity proteins are SuFu (combined with five other domains), Imm13, Imm33 and Imm-Ank (combined with four other domains) and, Imm11 and Imm34 (each with combinations to three other domains) (Figure 11C). The most frequently found domain combinations in polyimmunity proteins with more than one type of immunity domain involve combinations between one or more of the following immunity domains: SUKH, SuFu (including SuFu- family 2), Imm-Ank, Imm5, Imm33, Imm34, Imm36, Imm66, Imm67, Imm68 and Imm69. Like the polyimmunity loci, the polyimmunity proteins are encoded in operons, which usually do not contain associated toxin genes or cassettes. Interestingly, while polyimmunity proteins tend to be coded by small polyimmunity loci with two or three tandem immunity genes, they might not be found in the same bacteria with extended polyimmunity loci (see above) suggesting that the two are functionally related but distinct adaptations. Interestingly, some polyimmunity proteins have also been transferred to amoebozoan eukaryotes (Table 3, Additional File 1).

### **Contextual features: Functional implications of gene-neighborhoods and domain architectures**

**Figure 12:**
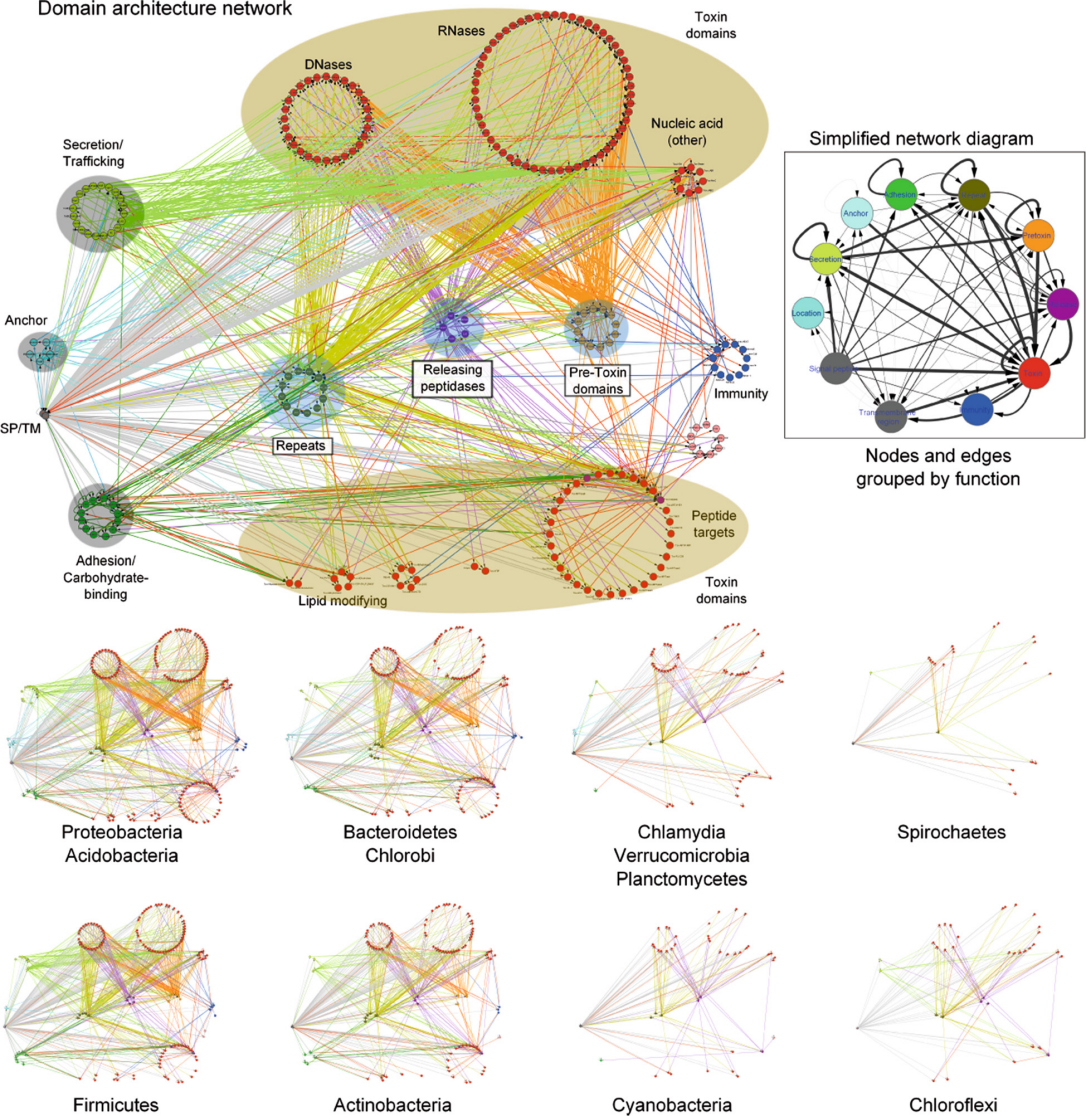
**Network derived from the domain architectures of toxins.** The central panel shows the network for all toxins in all species, whereas the lower panels show networks derived for major bacterial clades. The network is a directional graph with edges connecting neighboring domains in a polypeptide, in which the N-terminal domain is the source node, whereas the C-terminal domain is the target node. Edges are colored to match the source node color to illustrate the main direction of flow in the graph. Domains with similar properties are grouped together as shown.

To better understand the functional aspects of the genomic organization of the polymorphic toxins and related toxin systems in terms of genomic organization, recombination, secretion and interactions with immunity proteins, we resorted to a systematic analysis of their gene neighborhoods and domain architectures of toxins. For the sake of visualization, we represented the connections emerging from both these types of analysis as directed graphs: In the case of domain architectures, the nodes in the graph are the individual domains and the edges are connections between two adjacent domains in a polypeptide in the N- to C-terminal orientation. Each of the repetitive structures such as RHS and filamentous hemagglutinin repeats were treated as a single node (Figure 12). In the case of gene neighborhoods the nodes are individual genes or toxin cassettes and the edges indicate their neighborhood relationship in the 5’- > 3’ orientation (Additional File 1).

#### ***Inferences from the gene neighborhoods***

**Figure 13:**
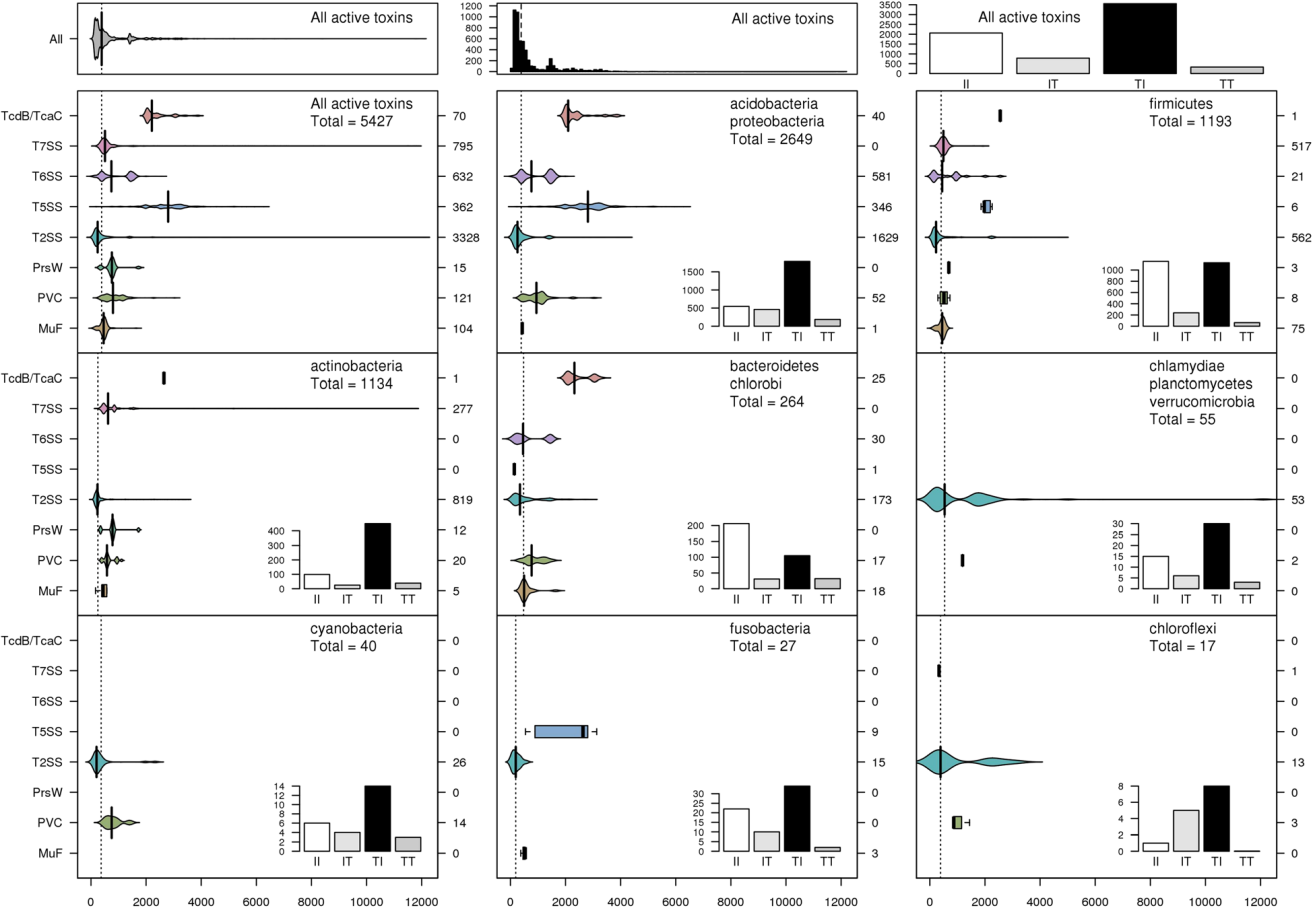
**Length distribution for predicted complete active toxins in different bacterial clades.** Complete active toxins, as against cassettes, were identified based on characteristic marker domains for each of the distinct secretory systems associated with the toxin either in the same polypeptide or in gene neighborhoods (Table 1). The topmost row shows the combined statistics for all active toxins while other panels present the breakdown of these distributions based on secretory bacterial clades. The toxin length distribution is represented as beanplot[182] (e.g. left panel in the first row) and a raw histogram (top row, central panel) and clearly indicates the multimodal nature of toxin length. The barplot on the first row (rightmost panel) shows the frequencies of consecutive toxin and/or immunity gene pairs in theses genomes. Only pairs of gene encoded by the same strand where considered. The labels indicate whether an immunity protein (I) or a toxin (T) is encoded upstream or downstream of its neighbor in putative operons, e.g. TI corresponds to a pair where an immunity gene is preceded by a toxin gene. Note that the TI (toxin - > immunity) architecture is the most frequent pair observed in all graphs except for bacteroidetes/chlorobi and firmicutes, where the presence of polyimmunity loci inflates the II category. Dashed vertical lines correspond to the median protein length for the data on each panel, and the solid vertical lines over each beanplot correspond to the median length in that secretory system alone. The axes at the right of each panel contain the number of active toxins per secretory system.

The one pervasive feature of polymorphic toxins across most gene neighborhoods was the predominance of the toxin-immunity gene (TI) order, wherein the toxin gene is to the 5’end, while the immunity gene is to the 3’ end of the operon (Figure 13). This tendency holds good for both complete toxin genes encoding all the N-terminal domains, as well as individual toxin cassettes which only encode toxin domains. There are several implications of this gene organization: 1) The toxin is synthesized prior to the immunity protein during translation. As the toxin protein is targeted to one of the many secretion systems for delivery to the cell surface, it is unlikely to cause immediate “self-intoxication”, thereby obviating the need for a premade immunity protein. This is supported in experiments with toxins exported by the T5SS, where the toxin is only activated in the target cell [183]. 2) Because polymorphism is achieved by recombining different toxin cassettes to a constant 5’ gene body coding for trafficking and presentation domains, there is the need for the recombination event to not only replace the 3’ toxin cassette [17,45], but also bring in its cognate immunity gene. This feature explains why cassettes also occur as TI pairs: On account of the TI organization of cassettes, a single recombination event at the 3’ tip of the complete toxin gene can replace the existing toxin coding region with a new toxin cassette and simultaneously bring in the new immunity gene. Evidence for multiple such recombination events is presented by the genomic organization of the full toxin genes. They often have a string of multiple immunity genes at the 3’ end [17]: each of these immunity genes is likely to represent a remnant of a former recombination even that replaced the tip toxin region while inserting a new immunity gene ahead of it. Thus, the lack of the need for a premade immunity protein due to outward trafficking of the toxin appears to have allowed the emergence of the TI gene order. The TI gene order in turn seems to have facilitated the emergence of polymorphism in these systems. Indeed the widely distributed simple barnase-barstar gene pairs might represent an incipient TI gene order without notable polymorphism, whereas the barnase cassette within larger polymorphic systems represents its incorporation into the fully developed versions of these systems.

The gene-neighborhood graph also contains the imprint of some of the secretory systems utilized for the outward trafficking of toxins by the producing cells (Additional File 1, Table 1)[18]. The complete toxin genes trafficked via the T5SS, T6SS, T7SS and PVC-SS often contain neighboring genes whose products mediate their trafficking. In the case of the T5SS the adjacent gene typically codes for CdiB-like proteins belonging to the TpsB class of outer-membrane trafficking proteins [37]. Such gene neighborhoods are only found in proteobacteria, bacteroidetes, fusobacteria and the negativicute clade of firmicutes (e.g. *Veillonella* and *Selenomonas*) and are strong markers indicative of the use of the two-partner system (T5SS) for the extrusion of toxins. The phyletic pattern of this system suggests that it might have emerged in the proteobacteria-bacteroidetes assemblage (members of the group I bacterial division [184]) followed by transfer to a subset of group II lineages such as negativicutes and fusobacteria. This supports the hypothesis that the negativicutes have secondarily acquired a “proteobacterial”-type cell wall through lateral transfer of specific components, and not as a by-product of the sporulation system as recently proposed [185]. The T6SS, PVC-SS, and MuF-SS utilizing toxins are typically marked by the presence of genes for the injection or capsid packaging apparatus, and a recycling AAA + ATP in the case of the former two systems [38,39,75,82]. Several T6SS operons additionally encode a PsbP/MOG1-like protein. The gene coding for the latter protein is often adjacent to the toxin gene and is related to the photosynthetic oxygen-evolving complex protein PsbP (p = 10^-17^; probability 98% in profile-profile searches) and might represent a novel subunit of the T6SS that acts as an adaptor between the secreted toxin and the injection apparatus. The genes of toxins secreted via the T7SS are occasionally characterized by gene neighborhoods that encode additional T7SS components such as the YueA-like FtsK/HerA ATPase (the motor driving T7SS), and EsaC, which contains a bacterial version of the PH-like fold [33,186]. Toxins associated with T7SS neighborhoods are found only in firmicutes, actinobacteria and chloroflexi, suggesting that toxins with this secretory mode possibly emerged early in the diversification of the group II bacteria (Table 1).

#### ***Inferences from domain architectures***

Comprehensive analysis of domain architectures of complete toxins reaffirms the results from the more restricted studies regarding the generally “tripartite organization” of the polymorphic toxins (Figure 1B): The N-terminal-most domains are related to trafficking of the toxin to the cell surface in the producing cell. The central domains, typically forming filamentous structures, are related to presentation of the toxin on the cell surface, and processing and release for delivery into the host cell. The C-terminal-most domains are the toxin domains. This architectural blue print might be violated in certain toxins that lack the central filamentous elements – these are usually shorter secreted proteins. N-terminal modules are usually associated with the secretory pathway taken by the toxin, with specific domains uniquely characterizing different secretory pathways (Table 1; Figures 12,13): 1) The TpsA-like secretion domain (TPSASD) defines the T5SS [37]; 2) the PVC metallopeptidase is determinant of the PVC-SS; 3) The WXG-like helical bundle (including LXG and LDXD) domains are strictly associated with the T7SS [187]; 4) the SpvB domain with integrin-like β-propeller domains are the determinants of the TcdB/TcaC export pathway [42]; 5) the PrsW peptidase domain defines the eponymous export system. In the case of the T6SS, the VgrG module, which form the tip of the injection apparatus [39], might be fused in certain cases to the N-terminus of the toxin protein. Although the VgrG module might be also found in the PVC-SS gene neighborhoods it is never fused to toxins secreted via this pathway. Additionally, our current analysis indicated that the conserved PAAR motifs (named after the eponymous signature found in a subset of these domains; PFAM: PF05488) with an associated TM helix is found in toxins strictly associated with T6SS gene contexts. This suggests that the PAAR motif is a determinant for T6SS-driven export. The PAAR motifs typically occur as pairs and each motif is predicted to form a 3-stranded element, with the second copy usually displaying conserved cysteines, histidines and an aspartate that might constitute a stabilizing metal-binding site (See Additional file 1 for alignment). Given their fixed N-terminal location in the complete toxins and their specific gene-context association with components of the T6SS, it is likely that the PAAR motif represents a signal recognized by this secretory pathway. The T2SS (general secretory pathway) is the most prevalent secretory system for polymorphic toxins (Figure 12,13). Of the dedicated secretory systems (i.e. those other than T2SS) we found that T7SS, T6SS and T5SS are the dominant ones, accounting for 12, 11 and 10 percent respectively of the complete toxins in our collection (Figure 13). The remaining dedicated secretory systems accounted for lower numbers of the total number of complete toxins. With respect to the ~150 distinct types of toxin domains we identified among polymorphic toxins and related systems, other than the general secretory pathway, the T7SS, T6SS and T5SS again dominate in terms of diversity of the C-terminal toxin domains with which they are associated (Figure 12). They are respectively being combined with 45, 43 and 43 percent of the total number of different types of toxins. Though the total number of toxin proteins delivered via the PVC-SS is much lower than that delivered by the three previously named systems, it is combined with a considerable diversity of distinct types of C-terminal toxin domains (31.5% of the total number of toxin types).

As discussed above, the two distinct positions of the processing peptidases, i.e., just prior to the toxin domain (e.g. HINT, papain-like peptidase, caspase) or at the N-terminus of the toxin protein (e.g. ZU5 and PrsW) appear to reflect two distinct functional themes in terms of autoproteolytic cleavage of the toxin protein. The HINT peptidase is found in association with T2SS, T5SS, T7SS and the TcdB/TcaC export pathway but never with the T6SS and PVC-SS (Table 1, Figure 12). This suggests that proteolytic processing by HINT and the PVC-metallopeptidase are mutually exclusive. This supports our above-stated inference that the PVC-metallopeptidase and the HINT peptidase are functionally equivalent. It also suggests that the injection process of the T6SS probably obviates the need for autoproteolytic action in toxin release. Of the repeats constituting the central filamentous regions, the filamentous hemagglutinin repeats are found only in toxins delivered via the T5SS. In contrast, the RHS repeats are found in toxins delivered by all the different secretory systems, except the T5SS. The less-common, central filamentous modules, which are also promiscuous in terms of secretion systems, include the phage tail-fiber and the alpha-helical ALF repeats. The HINT peptidase domain is found in association with representatives of all these different repeat types in classical polymorphic toxins suggesting that autoproteolytic processing to release the C-terminal toxin is a phenomenon that is independent of the type of the N-terminal stalk on which it is borne. A subset of toxin proteins from firmicutes, actinobacteria, proteobacteria and bacteroidetes are characterized by the presence of additional adhesion-related domains in their architectures (Figure 12). Most are carbohydrate or peptidoglycan binding and include the LysM, discoidin, Laminin-G, RicinB, bulb-lectin, PGB (peptidoglycan binding), CWB (cell wall binding) and SH3 domains [188-190]. The SH3 and laminin-G domains are usually found at the N-termini of the complete toxin proteins delivered by the T2SS and are likely to help in anchoring the toxin to the cell wall of the producing cell by binding components of the peptidoglycan or cell-surface carbohydrates. In contrast, RicinB, discoidin and bulb lectin domains might be found either at the N-termini or embedded among the RHS repeats or close to the C-terminal toxin module. This suggests that certain versions of these domains might also be used to enhance contact with target cells. Indeed, previously the RHS repeats have also been proposed to possess carbohydrate binding ability – hence, the RHS repeats might also directly participate in the adhesive action of the long toxins with such stalks [115,191]. The architecture graph also makes it clear that the nucleic acid-targeting toxins are the most prevalent type of toxin, far exceeding the peptide- and lipid- targeting toxins by a large margin (Figure 12). This is likely to be a reflection of the fact that a cell can be killed most effectively by disrupting the two key junctions in the flow of biological information, namely by disrupting the genome and by blocking translation.

 Examination of the length distribution of the complete toxins reveals a multimodal distribution with peaks of decreasing magnitude (Figure 13). The first peak is around 400, the second is between 1400–1600, the third is between 2200–2400 and the fourth is between 3000–3400 residues in length. The longest toxin recorded in our set is SACTE_5178 (gi: 345002682), with multiple toxin domains, from *Streptomyces* sp. SirexAA-E, and 13652 amino acids in length. This suggests that while the complete toxins cover a wide length range there are certain preferred lengths. In general terms it suggests that the polymorphic toxins are of two types: 1) stalked – those with long N-termini with multiple repetitive elements, which are likely to be used primarily in the contact dependent mode as described for the original CDI systems [17,36]. 2) Unstalked – these toxins lack a substantial N-terminal extension and are like to be secreted toxins that possibly act through diffusion into the environment or through directed delivery into the target cell [17]. The peaks of the distributions of the toxins delivered via the PVC-SS, T7SS and phage MuF-terminase system, are in the short range and these contribute in a major way to the first peak in the overall length distribution curve (Figure 13). In the case of the T7SS, while the majority of toxins are short and likely to be unstalked, there is a smaller set of longer stalked toxins which are also delivered by this system (Figure 13). The T6SS delivered toxins show a clear bimodal length distribution, with a shorter variety lacking stalks or fused to N-terminal HCP1 domains (Figure 13). This type contributes to the first peak seen in the overall length distribution curve. The second peak is around 1400–1500 amino acids in length (matching the second peak in the overall length distribution curve) and consists of stalked toxins with RHS repeats. This suggests that the T6SS delivers both unstalked and stalked toxins. The former are probably directly delivered into the target cell, whereas the latter are merely placed on the cell surface and might act through the contact-dependent mode. TcdB/TcaC-delivered toxins show a peak at around 2200 amino acids and contribute to the third peak observed in the overall distribution. The T5SS-delivered toxins show a peak a little after 3000 residues and contribute to the 4^th^ peak in the overall distribution (Figure 13). The toxins with RHS repeats show a peak in their length distribution around 1400–1600 amino acids (second peak in the overall distribution), while for the filamentous hemagglutinin repeats the peak length distribution is 3000–3400 amino acids (the fourth peak in the overall distribution) (Figure 13). This indicates that the major types of stalked toxins with different kinds of repeats, each have their own preferred lengths. This suggests that contact via such stalked toxins happens at a relatively constant distance from the cell surface. This in turn probably points to an optimal approach distance between neighboring cells in colonial aggregates, such as biofilms, where intra-specific competition would be expected.

### **Comparisons with other toxin systems**

The polymorphic toxin systems show several similarities and differences with other well-studied toxin systems of bacteria involved in different levels of intra-genomic, intra-species and inter-species conflicts. We compare below the polymorphic toxin systems with several of these systems and discuss the potential importance of significance of the similarities and differences:

1) *Effectors directed at hosts and distantly related competitors*: Mechanistically the polymorphic toxins and the effectors directed against hosts and distantly related competitors are closely related. These effectors are usually chromosomally encoded like classic polymorphic toxins. As seen from the above discussion (Tables 1,2), both these systems share a large number of toxin domains, processing peptidases, and also common secretory pathways including T2SS, T5SS, T6SS, T7SS, PVC-SS and TcdB/TcaC-like export. However, the T3SS and T4SS do not appear to be used by classical polymorphic toxins, even though they are common export pathways for effectors in specific bacterial lineages [34,192]. Some of them also have a structure closely resembling conventional polymorphic toxins and are only distinguished by the lack of associated genes for immunity proteins. Neighboring cassettes for standalone toxin domains are rare in these systems. However, the organization of other effector proteins sharing toxin domains with conventional polymorphic toxins might be different – the toxin domain is not necessarily located at the C-terminus and might occur internally or as a standalone protein. Additionally, these effectors also display certain toxin domains, such as those pertaining to the eukaryotic Ub-systems that are not deployed in classical polymorphic toxin systems used in intraspecific conflict. This reflects the relative rarity or the relatively limited functional penetration of sub-cellular systems by the prokaryotic cognates of the Ub-system [126], making them less effective targets for interference.

2) *Plasmid-encoded bacteriocins*: The plasmid-encoded bacteriocins, such as colicins, pyocins and cloacins conceptually resemble the classical polymorphic toxins in being deployed against closely related target cells. They also share the general architectural organization with classical polymorphic toxins – the N-terminal and central domains being deployed in trafficking with a toxin domain at the extreme C-terminus. Likewise, these systems are also characterized by immunity proteins that help protect the producing cells [20]. Not only do their toxin domains share several mechanistic themes, such as cleaving of DNA, RNA and perforating of membranes, with the toxin domains of polymorphic toxins, but they also share certain homologous toxin domains such as the HNH, ColE3 and BECR-fold nucleases such as the colicinD and ColicinE5 domains (Table 2). However, being on plasmids their primary function is to enhance the fitness of the carrying plasmid. Hence, they usually do not have dedicated systems for their export and depend on inducing lysis of a subset of the producing cells [20].

3) *Toxin-Antitoxin systems (Type I, II and III TA-systems)*: These systems might be encoded either on the chromosome or on a plasmid, and resemble the polymorphic toxin systems in comprising of a pair of elements with opposing activities. In the type II systems both the toxin and antitoxin are proteinaceous and interact physically with each other, thus being analogs of the polymorphic systems [22,24,28,193]. In contrast to the above described TI order of the polymorphic toxin systems with a 3’ immunity gene, in TA systems the antitoxin is typically the 5’ gene [22]. These elements are primarily intra-genomic selfish elements that are selected for maintaining themselves, and on occasions providing incidental advantage to the host cell [24,28]. Thus, they do not have a need for any kind of export trafficking and delivery apparatus that are encountered in the other systems. As a consequence both the toxin and antitoxin from these systems are small proteins, typically comprised of a single domain [22]. Nevertheless, certain toxin domains from the TA systems are homologous to toxin domains of polymorphic toxins. The chief examples of these are the RNases belonging to the BECR fold (see above), the RES domain, Ntox24 and DOC-like protein AMP/UMPylating enzymes. However, we currently do not have evidence for sharing of any of the metal-dependent nucleases between these two systems – the PIN domain nucleases are thus far only known from TA systems [108], whereas the REase, HNH and URI fold nucleases of the polymorphic toxin systems are not seen in the TA systems. On the whole, toxins of TA systems tend to predominantly target the genome and the RNAs of the translation apparatus [193], but those from the polymorphic toxin systems appear to have a much wider range, though even among them there is preponderance of nucleic acid-targeting activities that target the above functions (Figure 12). Peptidases are relatively rare in classical TA systems in comparison to the polymorphic toxins and their PVC-dependent relatives. However, in course of this study we uncovered a previously unknown TA system, which combines a toxin peptidase of the YabG family with a distinctive antitoxin which was previously annotated as a “domain of unknown function” (DUF1021). This adds to the pool of toxin domains that are shared by these systems. Another enzymatic domain shared by the toxins of type II TA systems and polymorphic toxins is the ART domain [148]. Interestingly, in this case the immunity protein or the antitoxin in both these systems might be an enzyme that removes the ADP-ribose modification, such as the ADP-ribosyl glycohydrolase. The immunity proteins from the type II TA systems, in addition to physically binding their cognate toxins, also usually act as transcription factors that regulate the expression of the TA gene-pair via their common promoter [22]. There is currently no evidence for any immunity proteins with a transcription factor function in the polymorphic toxin systems. In the case of the type I and type III TA systems the antitoxin is a small RNA that respectively interacts with the toxin transcript or the toxin protein [24,133]. Currently, there are no known polymorphic toxin systems with RNA regulators. It appears that the need for specific physical interactions between the toxin and antitoxin in most type II and III TA systems places certain restrictions on the types of toxin domains that can be incorporated into them – they typically are small domains that are not vastly different in size from the antitoxins.

4) *Restriction-Modification systems*: Like the TA systems, the R-M systems are mobile, intra-genomic selfish elements that operate in prokaryotic genomes [21]. Comparable to the cell-killing mediated by TA systems they have means of enforcing addiction by launching restriction attacks on cell if they are disrupted [194]. They resemble both classical polymorphic toxins and TA systems in combining a toxin (the restriction enzyme) with an antidote (the modification enzyme, typically a cytosine or adenine DNA methylase). However, unlike those systems the physical interaction between the modification enzyme and the restriction enzyme is not central to the counteraction of the latter’s toxic properties. Rather, since they operate on DNA, the antidote action of the modification enzyme is mediated by rendering the genome resistant to the restriction enzyme by preemptively modifying it. Being purely intra-genomic selfish elements, like TA systems, but unlike polymorphic toxin systems, they do not have any features related to trafficking or delivery. Instead, R-M systems display elaborate adaptations that enhance their target specificity and DNA-binding and manipulation capabilities in the form of specialized DNA-binding domains and accessory subunits such as helicases and MORC ATPases [120,195,196]. Nevertheless, as noticed above, R-M systems and polymorphic toxin systems appear to share several enzymatic toxin domains such as the REase, HNH, URI and ParB domains.

In conclusion, polymorphic toxin systems share certain key features with each of the other well-characterized prokaryotic toxin systems. The distinctions appear to arise from the differences in selective forces shaping each of these systems. On the whole the greatest mechanistic diversity of toxin and immunity domains are seen in the polymorphic toxin systems, which is reflective of the relatively few constraints faced by them in terms of their targets. However, certain types of catalytic domains are preponderant across several of these systems due to disruption of the genome or the translation machinery being apparently the easiest means of killing a cell.

### **Genome-wide distribution of polymorphic toxin systems and ecological implications**

#### ***Differences in distributions and structure of toxins and immunity protein: Phylogenetic and ecological tendencies***

**Figure 14:**
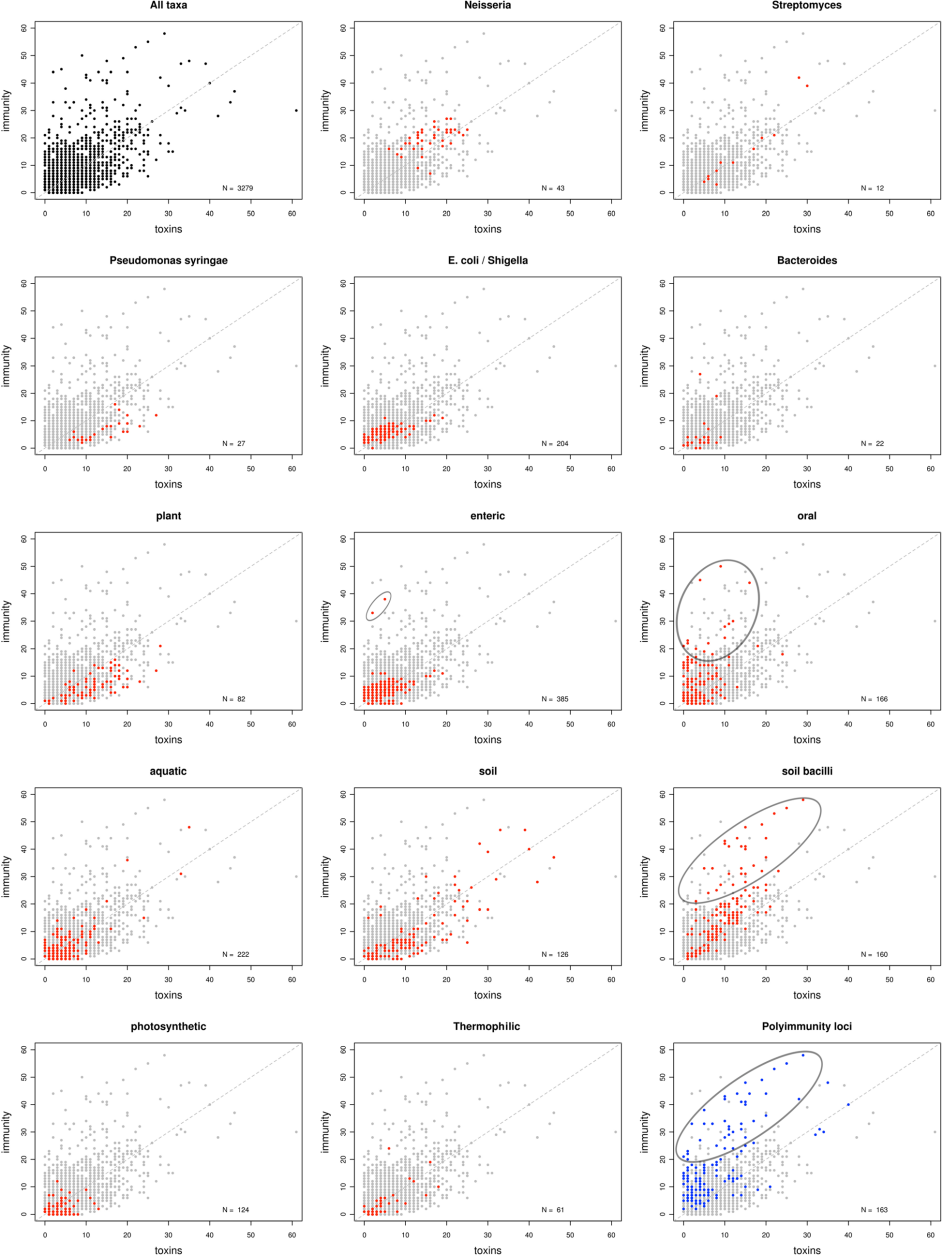
**Scatterplots of the number of toxins versus number of immunity proteins per genome.** In scatter plots, black or gray dots in the background represent all taxa, and red or blue dots correspond to taxa belonging to the clade or ecological properties described on each plot’s title. The dashed line corresponds to the diagonal (x = y) and the ellipses encircle taxa that are characterized by an excess of immunity proteins as discussed in the text.

To better understand the ecological significance of polymorphic toxins and related systems we systematically compared their genome-wide prevalence to organismal phylogeny. Our analysis revealed that all the major lineages of bacteria with sufficient genomic data had at least one representative coding for polymorphic toxin systems. However, the distribution of these systems between different bacterial lineages shows pronounced differences (Figures 13,14). Among the group-I bacteria [184], polymorphic toxin systems are abundant in the proteobacteria-like clade (including acidobacteria), bacteroidetes, and the clade unifying chlamydiae, verrucomicrobia and planctomycetes, but are relatively rare in aquificae and spirochaetes. Among the group-II bacteria [184], such systems are abundant in firmicutes, actinobacteria and chloroflexi but are relatively rare in cyanobacteria and thermotogae. They are generally absent in most archaeal lineages, with the rare exception of certain methanoarchaea and haloarchaea. Of these, *Methanosarcina acetivorans* displays classical stalked polymorphic toxins with RHS repeats and cassettes for toxin modules and immunity proteins, just as in the cognate bacterial systems. A few other methanoarchaea display simple barnase-barstar-like systems, whereas haloarchaea like *Halogeometricum borinquense* display several PVC-SS delivered toxins with variable C-terminal toxins modules (Additional File 1). This general rarity of the polymorphic toxin systems is in striking contrast to the general prevalence of the toxin-antitoxin systems across archaea [22]. This distribution, with a dominant presence in most major clades of both group-I and group-II bacteria, suggests that polymorphic toxin systems could have been present in the ancestral bacterium. However, it should be noted that these genes and cassettes are highly prone to lateral transfer as suggested by the sporadic phyletic distribution of both toxin domains and immunity proteins [17]. Hence, the distribution of these systems might also reflect in part the secondary dispersion of such systems across diverse bacteria by lateral transfer. In support of this it may be noted that in many organisms the polymorphic toxins are situated on hypervariable chromosomal islands that are prone to lateral transfer [197]. Nevertheless, distributions of the associated specialized secretory systems that deliver these toxins usually follow stricter phylogenetic boundaries, i.e. T5SS and T6SS occur primarily in group-I bacteria and T7SS in group-II bacteria. This suggests that indeed there might have been an ancestral presence of such polymorphic toxin systems in bacteria that selected for different dedicated delivery systems in each lineage and diversified further as these delivery system were fixed.

Certain patterns of distribution of polymorphic toxin systems appear to transcend phyletic boundaries (Figure 14): 1) the hyperthermophiles, which are often chemoautotrophs, from both bacteria and archaea show a strong tendency to lack such systems. 2) Likewise, the photosynthetic bacteria across different bacterial clades have a dearth of such systems (Figures 12,14; Additional File 1). The relative underrepresentation of such systems in both these groups of organisms is not related to their genome sizes because organisms with similar sized genome with other lifestyles do possess such systems. In particular, the relative rarity of such systems in cyanobacteria is striking when they are compared to other bacteria with multicellular tendencies and similar complex signaling mechanisms [65], such as deltaproteobacteria and actinobacteria, which in contrast possess abundant arrays of polymorphic toxin systems (Figures 12,14). While in the case of archaea it is possible that the rarity of these systems is related to their lack of bacterial-type protein uptake systems [20], it should be noted that bacterial hyperthermophiles show a similar pattern. The only exception is the firmicute *Geobacillus thermoglucosidasius*, which, unlike the rest, is not a classical hyperthermophile, and can survive across a wide temperature range [198]. It appears that the relative rarity of such systems might be more related to their phototrophic or chemolithotrophic tendencies. It is possible that that their relative independence with respect to energy, reducing equivalents and/or carbon dioxide results in lower levels of intra-specific competition for resources.

Finally, we also observed strong phylogenetic signals in the length distributions of complete toxins: 1) The group- I bacteria with Gram-negative cell walls with outer membranes (proteobacteria and bacteroidetes) had a multimodal distribution of complete toxins, showing both unstalked toxins and stalked toxins of various modal lengths (Figure 13). This suggested that they are likely to engage in both contact-dependent inhibition as well as inhibition via secreted toxins. 2) Firmicutes with the exception of the negativicute clade showed a largely unimodal distribution of complete toxin lengths with a median value of 492 residues. This suggests that the firmicutes deploy their toxins either mainly via secretion or through much closer contact than in the previous group. 3) The actinobacteria show a bimodal distribution of toxin lengths (Figure 13). The first peak is around 400–500 amino acids in length and the second is around 1400–1500 amino acids. This suggests that, like proteobacteria, they use both distant contact and secretion or close contact. The use of both short secreted toxins and longer contact-dependent toxins suggest that intra-specific conflict might play out both in the context of biofilms, where contact is critical, and also in motile phases and swarming growth, where contact might be less intense. The distinction in this regard between firmicutes and the two other groups raises question as to whether certain bacterial groups might resort to such forms of conflict only under specific circumstances.

#### ***Differences in the relative numbers of toxins and immunity proteins: Implications of intra- and inter-specific conflicts***

The median number of toxin domains found in organisms that possess such systems is 3, which is the same as the median number of immunity proteins found per genome (Additional File 1). The difference in the number of immunity proteins and toxin domains per organism is normally distributed with a sharp peak at 0 (Additional File 1). Furthermore, there is a positive correlation between the number of toxin domains and number of immunity proteins with an approximately linear increase in the number of immunity proteins with increasing number of toxin cassettes (Figure 14). These observations indicate that on the whole there is a balance between the number of toxin cassettes and immunity proteins, which is consistent with the genomic organization of the polymorphic toxin loci and the principle of approximately one-to-one mapping of immunity proteins with toxins. The number of active toxins is positively correlated with the total number of toxin cassettes, suggesting that with an increase in the number of individual polymorphic toxin loci the number of toxin cassettes associated with them increase more or less linearly (Additional File 1). The median number of active cassettes per organism is 1, indicating a median 1:3 ratio between active toxins and associated toxin cassettes.

We then studied the patterns of relative numbers of active toxins, cassettes and immunity proteins and their correlations, if any, with life-style and preferred ecosystems of the organisms. With exceptions discussed in the preceding subsection, bacteria across most well-sampled ecosystems display polymorphic toxin systems. However, we observed that a subset of organisms show strong anomalies in terms of the relative distribution of toxin domains to immunity proteins (Figure 14). We measured this anomaly using the difference between the number of immunity proteins and toxin domains and uncovered some striking ecological correlations. In general, in aquatic ecosystems we observed a strong proportionality in the number of toxins domains and immunity proteins, with roughly equal number of both (Figure 14). This suggests that in these niches there is a tendency for “honest” intra-specific conflict, with the polymorphic toxin systems primarily geared towards discrimination of non-kin conspecifics. Those organisms that showed significantly greater number of toxins than immunity proteins could be grouped into two general ecological niches: 1) *pathogens- Both extracellular and intracellular pathogens of animals, plants and microbial eukaryotes.* We interpret the relative abundance of toxins to immunity proteins in the former group as an adaptation for pathogenesis – the toxins are primarily used against hosts, rather than for intra-specific conflict; hence, many of their toxins do not have corresponding immunity proteins. This situation is especially prominent in intracellular bacteria such as *Waddlia chondrophilaLegionella* and *Amoebophilus asiaticus,* which have a large number of toxins but hardly any immunity proteins (Additional File 1). In general, the notable absence of immunity proteins in intracellular pathogens suggests that in most cases (baring exceptions like *Odyssella*) they do not engage in competition with conspecifics in their distinctive niche. In contrast, other pathogens of animals (e.g. *Neisseria* species), plants (e.g. *Ralstonia* and *Pseudomonas syringae*) and microbial eukaryotes (e.g. *Odyssella*), while showing a large number of toxins, also have comparable number of immunity proteins. This suggests that they are likely to compete actively with conspecific rivals in course of colonizing niches on or within their hosts. 2) *Slow growing, heterotrophic bacteria with a degree of “multicellular” organization, mainly actinobacteria and deltaproteobacteria*[65]*.* Organisms of this group are also well-known for their production of diverse non-proteinaceous antibiotics and maintain their slow-growing life-style by inhibiting competing faster-growing bacteria [5]. Thus, we see the over-representation of toxins relative to immunity proteins in this group as being part of their weaponry deployed in inter-specific competition. Importantly, both these groups are also enriched in organisms coding for the greatest number of toxin domains in their genomes. The greatest number of toxins is seen in different *Photorhabdus* species, which are nematode symbionts that aid nematodes in killing their insect prey [84]. Indeed, this bacterium is not only known to kill insects with their toxins, but also competes intra- and inter-specifically with other bacteria [199]. Thus, a large number of toxins domains might be a predictor for not just pathogen-host and inter-specific conflict but also intense intra-specific competition in certain niches.

On the other end of the spectrum we found several bacteria with an overrepresentation of immunity proteins relative to toxins. Especially striking were bacteria which showed a marked paucity of toxins but had a large number of immunity proteins, typically occurring in polyimmunity loci or as polyimmunity proteins. This group of bacteria is enriched in taxa belonging to the human oral microbiome (Figure 14; Additional File 1). Interestingly, this phenomenon was observed across bacteria belonging to phylogenetically distinct clades in the human oral microbiome: this group includes representatives of bacteroidetes (*Capnocytophaga gingivalis*), betaproteobacteria (*Eikenella corrodens*), spirochetes (*Treponema denticola*), actinobacteria (*Actinomyces* sp.) and firmicutes (*Streptococcus oralis*) (Figure 14; Additional File 1). This indicates that the oral environment has repeatedly favored proliferation of immunity proteins relative to toxins in a subset of bacteria across different clades. We interpret this imbalance in terms of the ecology of microfilms formed in the oral environment, where several bacteria are often packed in close proximity [200]. In this situation, non-kin “cheaters” which can invade microfilms to benefit from cooperative associations with proximal organisms can accrue an increase in fitness. Hence, we propose that the excess of immunity proteins in these organisms, particularly in the form of polyimmunity loci and polyimmunity proteins, is an adaptation to evade attack from a diverse array of toxins while invading non-kin bacterial assemblages. In support of this, we observed that there is a second group of taxa from the human oral microbiome that display relatively balanced ratios of toxins and immunity proteins (Figure 14; Additional File 1). It is likely that these organisms are the targets for invasion by the lineages with excess immunity proteins. Generalizing, this observation we propose that the presence of a large excess of immunity proteins over toxins might be a predictor for cheating behavior in invading non-kin bacterial assemblages.

A distinct second group of bacteria with a large excess of immunity protein differed from the above group in having a median or above median number of toxins. This group was greatly enriched in bacilli from soil such as *Bacillus cereus*, *B. mycoides*, *B. thuringiensis*, *Brevibacillus brevis* and *Paenibacillus polymyxa* and representatives of the human colonic microflora (Figure 14; Additional File 1). Even in this case, the excess of immunity proteins were typically associated with the presence of polyimmunity loci and polyimmunity proteins. Remarkably, we found that even within the same species (e.g. *B. cereus* and *B. thuringiensis*) different strains widely differed in the relative number of toxin domains to immunity proteins – some isolates had a considerable excess of immunity proteins, while other had a balanced ratio to toxin domains and immunity proteins (Figure 14; Additional File 1). This suggests that the different strains in a given species adopt two general strategies during intra-specific competition: 1) those which participate in “honest” cooperation between kin and discrimination against non-kin. These have similar numbers of immunity proteins and toxins because they possess only as many immunity proteins as required to balance their own toxins. 2) Those which adopt the strategy of cheating by invading non-kin assemblages. These varieties could potentially shift to the second strategy, by expressing their polyimmunity loci or proteins, when there is an excess of “honest players”, because in these situations cheating might become profitable. Notably, not all soil bacilli present an excess of immunity proteins over toxins, e.g. *B.subtilis* does not show the marked imbalance we observed in the above species. This predicts that there are likely to be differences in the social behavior of different soil bacilli, with species like *B.cereus* possibly engaging in greater degree of colonial or cooperative behavior throughout their life history. Further, the observation that the soil bacilli with an excess of immunity proteins have multiple toxins, unlike several of the above-described oral taxa which lack toxins, indicates that the context in which these groups might adopt a cheating strategy might differ. Among the oral taxa that lack toxins, it is conceivable that they have a phase in their life history where they do not engage in interactions with other bacteria. However, when they encounter target bacteria that can be invaded, they probably express their polyimmunity loci to interact with them while evading their toxins. In general terms, our findings might also explain how these organisms might escape collapse of the cheating strategy, which would happen when the numbers of cooperators are diminished. By facultatively expressing polyimmunity proteins or loci only when target cooperators are abundant and switching them off when they are absent, the deployment of the cheating strategy might be limited to advantageous circumstances.

### **Transfer of components of polymorphic toxins and related systems to eukaryotes and their viruses**

While eukaryotes deploy a wide-range of toxins, some of which share homologous domains with the polymorphic toxins and related systems, most of them do not seem to represent direct counterparts of the bacterial systems. The eukaryotic systems that come closest to the bacterial systems described herein are the fungal killer toxins such as the *Kluyveromyces lactis* γ-toxin and PaT secreted by *Millerozyma acacia* and *Debaryomyces robertsiae*[201-203]. Like the bacterial polymorphic toxins, these secreted fungal toxins are primarily used in conflict with closely related non-self strains and act as endo-tRNases. However, it should be noted that they are coded by linear plasmids, which makes them similar to the classical colicin-like bacteriocins, though, unlike them, release of the fungal toxins does not entail lysis of the producing cells. These endo-tRNases currently do not have any homologs outside of fungi and were not detected in any bacterial toxin system. Nevertheless, in this study we observed that at least 13 toxin domains from polymorphic toxin systems and their relatives have been laterally transferred to fungi (Table 2). This suggests at least a subset of these toxin domains of bacterial provenance might also be used by fungi in intra-specific conflict in a manner comparable to the above-mentioned, fungi-specific tRNases. Our earlier study of the deaminase toxins revealed that at least a subset of these, which were acquired by fungi, are probably used in intra-specific conflict, counter-selfish element defense or in phenomena related to heteroincompatibility [18]. Indeed, a major effector in the apoptosis-like heteroincompatibility process of several fungi, namely Het-C, appears to have originated from a bacterial toxin domain found in polymorphic toxin systems (see above).

The toxin domains from the bacterial systems also appear to have been acquired by animals and several other eukaryotes. At least 14 toxin domains observed in polymorphic toxin systems are also present in metazoans, whereas at least six are present in amoeboid eukaryotes belonging to the amoebozoan and heterolobosean lineages (Table 2). Experimental evidence in animals suggests that at least a subset of these, are deployed in antiviral defense and apoptosis. The AID/APOBEC deaminases are notable in the former context, though it appears that their role has further expanded in animals to encompass genome mutagenesis for generating antigen receptor diversity [204]. Like the fungal Het-C, on at least two occasions in metazoans, executers of apoptosis have emerged from toxin domains derived from polymorphic toxin systems – the DNA-fragmenting nuclease CIDE (a HNH fold endonuclease domain) [114] and the pierisin-like ARTs which ADP-ribosylate DNA [205,206]. The phyletic patterns indicate that the lateral transfer of these two toxin domains happened at very different points in animal evolution – the CIDE-like nuclease was transferred close to the base of the metazoa, whereas the pierisin appears to have been transferred only into the lepidopteran insects. Indeed, several of the toxin domains that have been sporadically transferred to eukaryotes could have been incorporated as lineage-specific components of apoptosis or antiviral defense systems. Of particular interest is the animal version of the Het-C domain which is currently known from chordates and the rotifer *Adineta vaga*. Like bacterial polymorphic toxins, it occurs in a cell-surface protein, which in vertebrates is encoded by the MHC class III region [207,208]. Given this architecture it is conceivable that it is deployed as a defensive toxin against fungal or bacterial pathogens. However, in certain cases, such as the GHH domain, which was acquired by animals, the toxin is no longer retained in its catalytic form; instead the catalytically inactive form is used as an extracellular signaling molecule (i.e. Od-Oz or teneurin). As noted above, the ADP-ribosyl cyclase appears to have been acquired by both metazoa and fungi from bacterial polymorphic toxin systems. In metazoa this enzyme was recruited as a signaling enzyme (prototyped by human CD38 and CD157), which generates two nucleotide messengers cADPr and NAADP that in turn regulate the influx of calcium via the ryanodine receptor [162,163]. Thus, the origin of multiple metazoan signaling messengers can be traced back to the polymorphic toxin.

Of note is the observation that several toxin domains of the polymorphic toxin systems are shared with effectors delivered by endo- parasitic or symbiotic bacteria. Given the widespread presence of such resident bacteria in cells of animals, amoeboid eukaryotes and ciliates [78,79,209], it is probable that such effectors are a major source of several of the toxin domains transferred to eukaryotes and their viruses (which might share the host cell with the intracellular bacterial residents; Tables 2). Indeed the toxin-like domains of effectors and polymorphic toxins deployed by several intracellular bacteria, such as *WolbachiaOrientiaRickettsiaRickettsiellaLegionellaOdyssellaAmoebophilusProtochlamydia* and *Hamiltonella* might affect the host evolution at various levels. In a very direct sense, their action might play a major role in the manipulation of host behavior, reproduction, sex ratio and fitness (e.g. defense against parasitoid wasps in aphids by *Hamiltonella*[100,101,144]). In certain animal lineages, such as the arthropods, the pervasive presence of endosymbiotic bacteria might facilitate the routine transfer of certain toxin genes, and appears to have contributed to the toxins of the arthropods themselves, as suggested by the latrotoxins of spiders. The acquisition of certain toxin domains by the mimiviruses (Tox-MCF1-SHE and Ntox19), iridoviruses (Tox-Otu domain), and several NCLDVs (Tox-JAB-2) suggests that they might be used by these viruses to manipulate host behavior in a manner comparable to the intracellular bacteria. Similarly, several toxin domains are also encountered in bacteriophages (Table 2), suggesting these viruses might also utilize toxin domains as a strategy to interfere with host physiology.

Certain endosymbiotic bacteria like *Odyssella* also contain full-fledged polymorphic toxin systems with both toxins and immunity proteins. Such endosymbionts could possibly explain the occasional acquisition of immunity protein domains by eukaryotes and their viruses (which might share the host cell with the resident bacteria; Tables 2,3). As previously noted, the SUKH domain proteins observed in several lineages of DNA viruses appear to have originated from immunity proteins of the polymorphic toxin systems [17]. Likewise, we had shown that the SuFu immunity protein has given rise to an intracellular component of the metazoan-specific hedgehog signaling pathway [17]. Our current analysis indicated that the C-terminal cargo-binding domain that is unique to animal type VI myosins is evolutionarily related to the immunity protein Imm-MyosinVICBD [210] (p = 10^-7^ in iteration 4 with JACKHMMER in a search initiated with an immunity protein gi: 332655030) that is predicted to counter certain ADP-ribosyltransferase toxins. Given that in eukaryotes the MyosinVICBD is only found in the animal lineage and in a single association, i.e. with myosin VI, it is likely it was acquired from bacteria through transfer of a gene encoding an immunity protein. Transport of cargo by the myosin VI is unique in that it is directed toward the minus ends of the actin filaments and is required for several key cellular differentiation events in eukaryotes [210]. Other than toxin domains and immunity proteins, processing components such as the HINT peptidase domain, have been acquired by eukaryotes and incorporated into several distinct eukaryote- or even animal-specific regulatory systems such as the hedgehog pathway [17]. Another example of a processing peptidase from polymorphic toxin-like proteins, the ZU5 autopeptidase domain, might have also contributed to the evolution of the animal apoptosis system – the two ZU5 domains are observed in PIDD, the core protein of the PIDDosome, which provides a platform for recognizing molecular patterns that are associated with loss of genomic integrity and genotoxic stress [211]. We observed that related ZU5 domains are also observed in a lineage-specifically expanded group of proteins from sponges, which might have a role in defense against pathogens (Additional File 1).

On a more general note, several endosymbiotic alphaproteobacteria such as *WolbachiaRickettsia* and *Odyssella* closely resemble the progenitor of the mitochondrion [212]. Thus, such endosymbiotic associations point back to the very origin of the eukaryotes. Similarly, other endosymbiotic associations, such as those with chlamydiae might have played an important role in the origin of the photosynthetic plant lineage [213,214]. Hence, it is conceivable that the origin of some of the eukaryotic systems might be related to acquisition of genes from the toxin systems of these early bacterial symbionts. We had earlier proposed that the PIN domain RNases of the eukaryotic nonsense-mediated mRNA decay system might have emerged from the prokaryotic toxin-antitoxin systems [22]. Similarly, the SUKH, Tad1/ADAR-like deaminase, the SuFu-associated HNH fold nuclease, ADP-ribosyltransferase and the ParBL1 domains might be early acquisitions from polymorphic or related secreted toxin systems of endosymbiotic bacteria, which were incorporated into various core function systems of eukaryotes [17,18]. In this context, it is tempting to suggest that the deubiquitinating peptidases such as those of the Otu clade, the Zu5 peptidase domain in the nuclear membrane protein Nup96/98, and the polyADP-ribose transferases (PARPs) might also be early acquisitions from polymorphic toxins or related effectors of the earliest endosymbionts in the associations leading to eukaryogenesis. Hence, it is conceivable that the very origin of certain features of the eukaryotic cell, and pan-eukaryotic regulatory systems such as ubiquitination and polyADP-ribosylation might have depended on domains derived from systems used in intra- and inter- specific conflict among prokaryotes. Thus, components derived from polymorphic toxins and related systems in symbiotic or pathogenic bacteria might have been critical for more than one major evolutionary transition in eukaryotes.

## Conclusions 

The current work is the first comprehensive analysis of the recently discovered polymorphic toxin systems. It builds upon our two earlier studies [17,18] that first uncovered these systems and revealed that their diversity was much greater than what was suspected in initial experimental studies [44]. In this work we have systematically identified the most prevalent toxin and immunity protein domains and have classified them based on sensitive sequence and structure analysis. This work thereby provides a framework for future studies on this exciting class of toxin systems. By creating an annotated inventory of toxins and immunity proteins it allows for further biochemical characterization of these proteins. In this regard, we offer a number of clear biochemical predictions in terms of the secretory mechanisms, the mode and site of action, enzymatic activities, active sites and possible catalytic mechanisms of toxins and immunity proteins. The systematic collection of toxins also aids their investigation as potential biotechnological and therapeutic reagents – a possibility underscored by the precedent presented by several other related toxins [4,7]. The pervasive relationship of toxins involved in intra-specific conflict to those used by bacteria in inter-specific conflict, such as toxins directed against hosts, is highlighted in this study. Thus, the results presented here also help in understanding the pathogenesis of numerous plant and animal pathogens, as also the interaction between unicellular eukaryotes and their abundant intracellular bacterial residents. These findings might have considerable significance for our future understanding of the virulence of key pathogens, such as *Pseudomonas aeruginosaLegionella*, and rickettsiae among other animal pathogens, and *Pseudomonas syringaeXanthomonas* and *Ralstonia* among plant pathogens. The toxins characterized here also provide insights regarding the biochemical basis for complex multi-organism interactions, such as the role for *Hamiltonella* in defense against parasitoid wasps and *Photorhabdus* in nematode predation of insects[84,100,101,144,199].

This study offers a platform for understanding certain key ecological aspects of bacterial interactions. Systems characterized here suggest, for the first time, possible molecular determinants for phenomena such as kin versus non-kin discrimination, cooperation and cheating both in the context of biofilms and motile growth. The ideas presented here allow for several testable microbiological hypotheses regarding bacterial conflicts. For example, the proposal regarding cheating in diverse taxa from the oral microbiome and certain soil bacilli can be tested via relatively straight-forward competition experiments. Indeed, such experiments can test our proposal if the polyimmunity loci and proteins facilitate a facultative cheating strategy in interactions between conspecifics. The systematic characterization of these loci also allow for further exploration of the rates of polymorphic transitions of toxins under different conditions and in different ecosystems. Some of these studies might have considerable bearing in human, non-human animal and plant health, because they might help explaining the preferential colonization of bodily niches by certain strains as opposed to others [15,199]. This might be of considerable value in facilitation of processes such as wound healing and appropriate re-colonization of bodily niches after antibiotic therapy.

The immunity proteins from these systems also offer a means for understanding the two contrasting aspects of the evolution of protein-protein interfaces. Our earlier study had shown the versatility of the SUKH and SuFu domain immunity proteins in interacting with a diverse array of structurally and mechanistically distinct toxin domains [17]. Thus, they join the previously studied scaffolds such as the immunoglobulin domain and LRRs in vertebrate antigen receptors as models to understand how a single structural scaffold can diversify to accommodate an enormous variety in protein-protein interactions [178]. On the other hand, we have also uncovered numerous immunity proteins that are specific in terms of the toxins they counter. Furthermore, a notable majority of these immunity proteins are apparently unique to these systems. This presents them as models for the converse aspect of the evolution of interactions, i.e. how a large number of distinct domains with very specific interfaces for interaction have emerged apparently *de novo* in these systems. Further investigation of immunity proteins through a combination of structure determination studies and biochemical analysis would be of greatest interest in regard to the evolution of these specific protein-protein interaction capabilities.

Finally, the analysis of the diversification of components from polymorphic toxins and related systems points to a previously underappreciated evolutionary principle. Several toxin, immunity protein, structural modules and secretory components from these systems have a distinct life beyond their locus of provenance, especially in eukaryotic regulatory and defense systems. We have documented that on numerous occasions components from these systems were incorporated into regulatory systems of eukaryotes, and in many cases might have played a major role in the very origin of some of these systems [17,18]. Thus, these systems appear to be particularly rich sources to draw from for new functional innovation. We attribute this to the consequences of natural selection in systems related to inter-organismal or intra-genomic conflicts. Not surprisingly, such toxin-immunity systems have a large effect on the fitness of organisms [15,44], thereby escalating an arms race situation. This has resulted in a strong selective pressure for constant diversification of polymorphic toxins and their immunity proteins. Thus, such systems have acted as a “nursery” for innovations in the protein world. Given that such conflicts often extend to the sphere of symbiotic and parasitic interactions with eukaryotes, the latter have access to a “readymade” set of molecular innovations from such systems, which can be recruited to spur the emergence of new interactions in eukaryotic systems. This is consistent with the similar diversification seen in other systems involved in intra-genomic or inter-organismal conflict [5,127,196,215,216]. These include antibiotic biosynthesis systems which are used in inter-specific conflict, siderophore biosynthesis systems whose diversification helps prevent siderophore-stealing by “cheaters”, R-M and TA systems involved in intra-genomic conflict[5,21,194,217]. Indeed, our earlier studies indicated that components from each of these conflict systems have played a major role in contributing components to diverse eukaryotic regulatory systems [127,196,215,216]. Thus, organismal and genomic conflicts being the basis for major molecular innovations, which in turn might facilitate major evolutionary transitions, can be considered a general evolutionary principle.

## Methods

As described in the search strategy, protein sequences corresponding to predicted toxins, trafficking, presentation, processing and immunity domains were isolated using diagnostic domain architectures and gene-neighborhood templates, that were initially identified in previous studies [17,18] (Figure 1). The sequences of representatives of each of the domains from toxins, immunity proteins and associated trafficking components were then used as seeds in iterative profile searches with the PSI-BLAST [218] and JACKHMMER [219] programs that run against the non-redundant (NR) protein database of National Center for Biotechnology Information (NCBI), to identify further homologs. A list of these search-seeds and the residue ranges for each domain is provided in Additional file 1. For most searches, which were used to report the relationships presented in this work, a cut-off e-value of .01 was used to assess significance. In each iteration the newly detected sequences that had e-values lower than the above cutoff were examined for being false positives and the search was continued with the same e-value threshold only if the profile was uncorrupted. The postulated relationships recovered using such iterative searches were further confirmed with other aids such as secondary structure prediction and superposition on known structures, if available. This resulted in the identification of over 250 toxin and immunity domains. Search results for these domains are provided in Additional file 1.

For each toxin or immunity gene, the gene neighborhood was also comprehensively analyzed using a custom Perl script of the inhouse TASS package. This script uses either the PTT file (downloadable from the NCBI ftp site) or the Genbank file in the case of whole genome shot gun sequences to extract the neighbors of a given query gene. Usually we used a cutoff of 5 genes on either side of the query. The protein sequences of all neighbors were clustered using the BLASTCLUST program (ftp://ftp.ncbi.nih.gov/blast/documents/blastclust.html) to identify related sequences in gene neighborhoods. Each cluster of homologous proteins were then assigned an annotation based on the domain architecture or conserved shared domain. This allowed an initial annotation of gene neigborhoods and their grouping based on conservation of neighborhood associations. The remaining gene neighborhoods were examined for specific template patterns typical of toxin-immunity systems. In this analysis care was taken to ensure that genes are unidirectional on the same strand of DNA and shared a putative common promoter to be counted as a single operon. If they were head to head on opposite strands they were examined for potential bidirection promoter sharing patterns.

Multiple sequence alignments of all domains were built by the Kalign [220], Muscle [221] and PCMA [222] programs, followed by manual adjustments on the basis of profile-profile and structural alignments. Secondary structures were predicted using the JPred [223] and PSIPred [224] programs. A comprehensive database of profiles was then constructed using these multiple alignments and was used extensively in the annotation and analysis of protein domain architectures and gene neighborhoods. For other known domains, the Pfam database database [189] was used as a guide, though the profiles were augmented in several cases by addition of newly detected divergent members that were not detected by the original Pfam models. Clustering with BLASTCLUST followed by multiple sequence alignment and further sequence profile searches were used to identify other domains that were not present in the Pfam database. Signal peptides and transmembrane segments were detected using the TMHMM [225] and Phobius [226] programs. The HHpred program [227] was used for profile-profile comparisons to either unify poorly characterized families to proteins with a known structure in the PDB database or to group related families of toxins or immunity domains. Structure similarity searches were performed using the DaliLite program [228]. Phylogenetic analysis was conducted using an approximately-maximum-likelihood method implemented in the FastTree 2.1 program under default parameters [229]. Predicted lateral transfers to eukaryotes were further evaluated for false positives by ensuring they were embedded in contigs or complete chromosome sequences with other genes typical of eukaryotes, comparing exon-intron structure of the genes, studying their phyletic distribution within eukaryotes and comparing the protein distances of the predicted eukaryotic proteins (as measured by bit scores) with bacterial homologs. Structural visualization and manipulations were performed using the VMD [230] and PyMol (http://www.pymol.org) programs. Automatic aspects of large-scale analysis of sequences, structures and genome context were perfomed by using the in-house TASS package, which comprises a collection of Perl scripts. Supplementary material can also be accessed at ftp://ftp.ncbi.nih.gov/pub/aravind/TOXIMM/toximDBsupplementary.html.

## Competing interests

The authors declare that they have no competing interests.

## Authors’ contributions

DZ, LMI and LA designed the study; DZ, LMI, VA and LA obtained the data; RFdeS wrote the custom scripts for analyzing, managing and interpreting the data; DZ, LMI, RFdeS and LA performed data analysis and interpretation; LA wrote the manuscript with inputs from DZ; LMI prepared the tables; DZ and RFdeS prepared the figures. All authors read and approved the final manuscript.

## Reviewers’ comments

Reviewer 1: Dr. Igor Zhulin (Oak Ridge National Laboratory, USA)

I have conflicting views on this paper. On one hand, I have read Introduction, the beginning of Results & Discussion (the authors lost me half through this section though as it become very descriptive and I had a hard time connecting the pieces), and Conclusions with a great interest. The topic is fascinating and the amount of work that has been done is unbelievable. The authors analyzed an enormous amount of data, both published and results of their computational research, and presented not only a catalog of proteinaceous toxin systems, but a multi-scale picture of their roles in various biological processes. On the other hand, it all came at a high price of lacking necessary details regarding computational analyses and focus. I perfectly understand that presenting such a huge amount of information requires sacrifices in some areas, but I do not think that it should be in describing “experimental procedures”. It is a generally accepted policy in science that procedures must be presented in a sufficient detail, so experiments can be independently reproduced. This paper, in my opinion, does not fulfill this requirement. The section “Search strategy to identify new toxins and immunity proteins”, which serves the purpose of providing such details, gives only a very general description.

***Authors’ response****: We have altered the Material and Methods to provide more extensive details regarding the procedures we followed with respect to sequence and structure analysis. We do not agree with the referee’s statement that experimental procedures have been sacrificed. In essence all the sequence and structure analysis was performed using publically available programs, which have been published and are well-known in the computational biology community, if not more widely. In the current version of the Material and Methods we describe these without omission and any reader with access to appropriate computer resources can use the same. We also disagree with the referee’s allegation of the lack of sufficient information for independent reproducibility – see below for further details in this regard.*

Finally, the length and overall organization of this paper makes it very difficult to follow it through and the lack of page numbers is inexcusable for a manuscript that has 130 of them. Nearly each of the 38 subchapters of this paper has its own introduction and reads as a separate story. As a result, we do have an encyclopedia of polymorphic toxin systems, but its true scientific quality is hard to estimate.

Personally, I would rather see much smaller pieces of this work presented in a concise way with all details of searches and analyses clearly shown. The global view that authors aimed at presenting is much better suited for review papers. Here we have a lot of original work mixed up with a review of literature: the number of references in this paper is higher than in many comprehensive reviews on similar topics. I think the quality of both original work and review suffers from this mix.

The bottom line is that to me this is a paper that reaches very interesting conclusions, but which is very difficult to comprehend in its entirety and some (if not many) of its results cannot be verified (or are very difficult to verify) independently.

***Authors’ response****: We regret the inconvenience caused by the lack of page numbers, which stems from using a PDF reader which provides the page numbers as against a print version. The referee raises three basic issues which we address below-*

*(i) Length of the article – single long* versus *multiple short papers: Short articles are useful when a single domain or computational observation needs to be succinctly presented. Indeed, upon our initial discovery of these systems we published two shorter articles outlining just the details of specific aspects of them. However, upon further investigation it became clear that neither those two works nor subsequent experimental studies on these systems really do justice to the magnitude of domain diversity seen in these systems. Unlike many other systems, despite these proteins being around and accumulating in the non-redundant protein database for now more than a decade, there has been hardly any comprehensive study on them. This is testified by the rather rudimentary annotation borne by most of them in protein databases. This being the first such treatment on a long-neglected class of highly represented proteins meant a particularly long paper. Furthermore, the practical aspects of publication meant it was quite infeasible to prepare numerous separate small papers and submit each for peer-review. We realized in course of our study that splitting the individual discoveries into multiple manuscripts would dilute the big picture emerging from these systems. With respect to shorter works being easier to read than a comprehensive manuscript as this we opine that it is largely a matter of taste. It may be noted that referee two, despite finding the length remarkable, commented regarding its easy readability. The apparent self-sufficiency of the sub-sections is primarily to help readers who might be more interested in one or few of toxin or immunity domain families but the text has been edited to minimize redundancy. Hence there is no repetition of material between sections.*

*(ii) Review* versus *original paper admixture: We disagree with the referee in saying that it is a mixture of review and original research. The “review” aspect is limited to the introduction and general conclusions, as is typical of any research paper. It should be kept in mind that any kind computational analysis work based on sequence/structure analysis needs to place newly identified domains in the context of what is already known in order to make new functional predictions. This is exactly what we do – this necessitates the mention of previous studies and also precedence of biochemical activities for functional inference. We do not see this as being a mixture of review with new results but merely an aspect of building a functional argument. While there are several domains and ideas presented in this study, we were particular in only emphasizing those that are novel and discovered in this study. In our calculation, ~ 85% of our dataset (that has about 250 toxin and immunity domains) is not found in any domain database. Those that are already present in protein domain databases like PFAM, they are typically listed as domains of unknown function (DUFs) and are need of functional annotation.*

(iii) Reproducibility: As noted above, we do not accept the claim that our results are not reproducible. Of course, the ease of reproducibility depends entirely on the time available to one attempting it. We should emphasize that all the computational discoveries reported here use standard sequence/structure analysis techniques laid out in the Material and Methods, as is typical of a paper in this field. Those cases involving more difficult detections we explicitly mention in the paper program used and statistical support for the particular relationship or the Z score cutoffs used by DALIlite for structural relationships. Since we have provided Genbank identifiers (gis) for the prototypical proteins of every group, all the remaining relationships can be reproduced by running profile searches with PSI-BLAST, HMMsearch3, JACKHmmer or HHpred on the Web or locally, either in a unidirectional or transitive fashion. Most importantly we have provided one of the most extensive supplements for a sequence/structure analysis paper -- alignments for each toxin and immunity domain have been provided; hence, obtaining starting points for reproducing searches should not pose any difficulty. The gis of all proteins under consideration are also provided along with an appropriate classification. This allows for independent verification of architectures and operonic associations. In addition to the extensive tables in the body of the article which provide details regarding active sites and phyletic patterns, the data is also provided in the supplement as searchable tables, where readers can browse the data by species, domain, operons, and pathway of secretion. We fear the referee did not peruse the extensive supplement that provides all the material for reproducing the presented analysis. In the revised version we have further improved the presentation of the supplement to improve ease of access to the alignments. We will also upload all the new alignments to protein databases such as Pfam making the material available upon publication to facilitate easy reproduction and use of the presented results.

**Reviewer’s reponse to above**:

I am not persuaded with authors’ arguments regarding their description of “experimental procedures”.

Let me consider just the first paragraph of Materials and Methods, which is shown below (in italics) in its entirety and is fragmented only by my interjections.

*As described in the search strategy, protein sequences corresponding to predicted toxins, trafficking, presentation, processing and immunity domains were isolated using diagnostic domain architectures and gene-neighborhood templates, that were initially identified in previous studies*[17,18]*(*Figure 1*). These domains were then used as seeds in iterative profile searches with the PSI-BLAST*[217]*and JACKHMMER*[218]*programs that run against the non-redundant (NR) protein database of National Center for Biotechnology Information (NCBI), to identify further homologs.*

This is a very general statement, which provides very little detail. Cleary, each PSI-BLAST and JACKHMMER search is carried out not with “domains”, but with one concrete protein sequence, which has a name and coordinates of the region that was used as a query.

***Authors’ response****: We concede that the word domain in this context might be confusing for some readers. However, it is should be noted that in this context we obviously imply the amino acid sequence corresponding to a given domain. This point has been emended.*

A search is performed against a specific database of a certain size and content. The size of NR database has doubled in less than 3 years and is changing every day. Thus, it is important either to work with a fixed version of NR or to report which version was used in a given search. Here is the excerpt from the authors’ own work, which provides a good example of how “experimental procedure” should be described:

“A PSI-BLAST search was initiated with the conserved N-terminal extension of the SGC (human SGC1β, gi: 4504215, region 1–360), using an inclusion threshold of .01, and compositional bias based statistics to eliminate false positives arising due to peculiarities of sequence composition. Both the N- and the C-terminal parts of this extension gave several distinct hits to different bacterial proteins, supporting the presence of two distinct globular domains in this extension. Based on these hits we divided the extension into N- and C-terminal parts and initiated separate PSI-BLAST searches with them. Searches with the N-terminal part of the extension gave significant hits to bacterial proteins of the length 180–195 residues within the first 3 iterations (eg. Mdge1313 from Microbulbifer degradans is detected with an expect-value (e) of 10–4 in the first iteration)…” (LM Iyer, V Anantharaman and L Aravind 2003 BMC Genomics 2003 4:5)”. Although some details are still lacking and the NR version was not specified (not that critical for the year 2003), this description is thorough enough to reproduce the steps that were taken during the domain identification process. I regret that ten years later authors think that providing search details is no longer necessary. Once again, I understand the reason for not providing details for numerous searches that they have carried out, and once again I disagree with this position.

***Authors’ response****: We appreciate the referee quoting from a former work of ours. Obviously we have neither forgotten nor changed our philosophy to domain discovery or analysis in the past 8 years. We note that the referee states that he understands why we do not give these details in the same manner as it is done when reporting the discovery of a single/few domains. We should reiterate that when such an analysis is scaled up to hundreds of domainsf providing descriptions as that pasted by the referee would result in an extraordinary and tedious prolixity for most readers (users) of the article. Hence, the report in the actual manuscript focuses on the points of biochemical/biological interest with only a general description of the search strategy for most cases. This does not mean that the issues raised by the referee are inaccessible. They are simply provided in the supplementary material. Herein a reader might find a collection of the actual saved PSI-BLAST searches for all the notable domains described herein. The same files should supply the specifics of the nr database at the point of the run. Furthermore, another file in the supplement provides the query gi with sequence coordinates of all seeds used for the domain-specific searches. Yet another file provides the searches with all the profiles, which we created for this work (either PSI-BLAST or HMM) against the NR database from May 23*^*rd*^*2012. The links have been made explicit in the additional file.*

***Referee’s comment resumes***: *For most searches in which were used to report the relationships presented in this work a cut-off e-value of .01 was used to assess significance.*

Let us leave alone the fact that something is missing from this sentence (what were used?) and focus on the main point. This statement means that for some searches a cut-off E value other than 0.01 was used.

***Authors’ response***: *This sentence had a typo which we have now corrected and appreciate the referee pointing the same.*

FOR WHICH ONES? WHY? No details provided. Furthermore, 0.01 is already a “dangerous” level, when it comes to false positives. The description provided by authors leaves a possibility that some searches were carried out even with a worse E value. It does not automatically mean the results are incorrect, but it does mean that a special care must be taken when considering such relationships and description must be provided.

***Authors’ response***: *The .01 cutoff is dangerous only in the hands of the untrained sequence analyst. Obviously we took special care to manually examine every iteration of searches with every domain reported in this study. Thus, we ensured that the new sequences being included are unlikely to be false positives.*

***Referee’s comment resumes***: *This was further confirmed with other aids such as secondary structure prediction and superposition on known structures, if available. For each toxin or immunity gene, the gene neighborhood was also comprehensively analyzed using a custom Perl script of the inhouse TASS package. The process was carried out iteratively and exhaustively and resulted in the identification of over 250 toxin and immunity domains.*

I am guessing that the first sentence refers to assessing the validity of multiple sequence alignments (which is described in the next paragraph). This indeed is a common technical element, which requires no further description. However, the next sentence makes quite a difference. What is meant by “comprehensive analysis of the gene neighborhood”? How many genes in the vicinity of the gene of interest were analyzed? How were they analyzed: by their RefSeq annotation? COGs? Best BLAST hit? Gene neighborhood analysis is a very important element of computational genomics of prokaryotes; however, there is no publically available, published program or even a single, commonly accepted approach on how to do this analysis. Thus, it is important to provide details.

***Authors’ response***: *The Material and Methods have emended to include further details on neighborhood analysis.*

“*The process was carried out iteratively and exhaustively…*” Which process? The entire process of domain identification or only the PSI-BLAST searches? I understand how the latter can be done iteratively and exhaustively, but I can only guess what it means with respect to the entire process, and certainly cannot distinguish between these possibilities.

***Authors’ response***: *The Material and Methods have emended to remove the potential confusion arising from this statement.*

In response to my original critique authors replied that they “do not agree with the referee’s statement that experimental procedures have been sacrificed. In essence all the sequence and structure analysis was performed using publically available programs, which have been published and are well-known in the computational biology community, if not more widely”. In essence, yes, but in some cases, obviously, no: a custom Perl script of the in-house package… Custom scripts execute specific actions. We do not need to know what the script is, but we certainly do need to know what the action was. “Comprehensive analysis of gene neighborhoods” to me is a prototype example of sacrificing the description of “experimental procedures”. Even when it comes to publicly available and published tools, procedure details should be provided. In experimental biology, it is not enough to state that PCR was used to amplify a given gene – exact primers must be provided. Perhaps, this is not the best analogy, but it illustrates the point.

***Authors’ response***: *The Material and Methods have been emended to describe the action of the script which in essence provides the details pertaining to the gene-neighborhood analysis raised above.*

On the final note, I would like to emphasize that I have an utmost respect for the authors, who have been leaders in the field for many years now, and who produced a series of groundbreaking papers in computational genomics. Without doubts, their results and conclusions are both correct and important. Furthermore, I applaud their decision to submit all domain models to the public repository (Pfam). However, I do disagree with their position on attention to detail in describing “experimental procedures”. I can expand on this point substantially; however, this is not the place for such a debate.

***Authors’ response***: *We too believe that this is not the place for a general debate on methodology.*

Reviewer 2: Dr. Arcady Mushegian (Stowers Institute for Medical Research, USA)

The manuscript by Zhang et al. is a magisterial treatment of a large and heterogeneous group of bacterial complex toxin proteins as well as the immunity proteins that countervail the action of these toxins. It is a comprehensive collection of old and new protein families, genome contexts and phyletic distributions of these important functional modules in prokaryotes, which also crosses over to partially analyze the sequence relationships of secretion systems in bacteria. I have no concerns about the quality of sequence comparison, domain definition and genome context analysis. This is a catalog of novel predicted functions, which can guide the work of experimentalists for years to come. I do have, however, several small concerns about data presentation and some comments that have to do with the broader discussion of bacterial evolution. More specifically:

***Authors’ response***: *We thank the reviewer for his positive comments and suggestions*.

p. 21–22: a few homologs of multidomain polymorphic bacterial toxins are purported to be present in eukaryotes (e.g. gi 321474287 in Daphnia and Tox-REase-8 in a subset of insects), and it is surmised that they have been horizontally transferred from bacteria. How do we know that these genes are indeed found in the genomes of these eukaryotes, and do not represent endosymbiont DNA or other contamination? Have the genomic contigs been assembled, do these genes display eukaryotic features - e.g., introns?

***Authors’ response****: In our analysis, we were particularly careful in eliminating false assignments of lateral transfer to eukaryotes and used several parameters to decide if the laterally transferred genes were indeed encoded by the eukaryotic species. In the simplest scenario, the presence of introns was indicative of their eukaryotic presence. For example, the gene for gi 321474287 in Daphnia contains 11 introns, whereas most Tox-REase-8 genes in insects at least contain one intron, eliminating the possibility of these genes being contaminants. Other parameters that were considered include: 1) Elimination of sequences that were identical or almost identical to bacterial sequences. In our dataset, none of the proteins assigned as laterally transferred showed any identities or near identities to bacterial sequences; 2) Most proteins assigned as laterally transferred to eukaryotes also showed a presence in more than one eukaryotic species, which further helps in eliminating false lateral transfer assignments. For* e.g. *Tox-REase-8 is present in crustaceans, insects and placozoans. Similarly, Tox-GHH domains are present in five major lineages of bacteria, while in the eukaryotes they are only found in multiple metazoan species (TCAP domains of teneurins). In response to this comment and to that made by Reviewer 3, we have explained this procedure in more detail in the Materials and Methods.*

p. 44–45. The gene neighborhood network shown in Figure 12: I am not sure what it is supposed to visualize. The authors state that the direction of the edges is important, i.e., it shows the 5' to 3' order of genes or protein domains; but the arrowheads are barely visible even in the pdf at magnification 250%, and will not be seen online. In any case, the edge density is so high that the main message seems to be 'anything can link to anything'. The graphs become more sparse when clade-specific connections are shown - this is more interesting, but perhaps visualization would be better if the density of connections is modeled by the edges of different thickness.

***Authors’ response****: We agree with the reviewer that the full view of the domain architectural network was too dense for a detailed view. We have now added a simplified graph next to the central graph that further combines all nodes into metanodes based on their functional type. This simplified graph gives a better view of the follow on connectivities across all toxin polypeptides. For example, it clearly shows that toxin domains detected in this study are almost always at the C-terminus of the protein.*

The next several comments have to do with somewhat superficial and inconsistent discussion of relative plausibility of various evolutionary scenarios.

To wit:

p. 46 "The phyletic pattern of this system suggests that it might have emerged inthe proteobacteria-bacteroidetes assemblage (members of the group I bacterial division [183]) followed by transfer to a subset of group II lineages such as negativicutes and fusobacteria." --- Why not the other direction, or ancestral origin followed by gene losses (especially given that these scenarios are discussed later for essentially the same phyletic vectors)?

***Authors’ response****: The above argument is based on parsimony. In this study, we notice a strict correlation between the occurrence of T5SS and the presence of an outer membrane. Most lineages from Group I bacteria (including all proteobacteria and bacteroidetes) contain an outer membrane and also components of T5SS. In contrast, most lineages of Group II bacteria contain only one membrane layer around the cell further encapsulated by a cell wall. Some exceptions include the negativicutes which are a subset of firmicutes that have an outer membrane. Since the ancestral state of the Group I and Group II bacteria can be generally reconstructed as possessing an outer membrane in the former and containing a single membrane layer in the latter, we propose that the T5SS were laterally transferred to the negativicutes and fusobacteria .We have added an additional remarks in this regard in the revised manuscript.*

***Referee’s further response***: *The explanation is fine in this case, but compare it to the following point-counterpoint.*

p. 52–53: "This general rarity of the polymorphic toxin systems is in striking contrast to the general prevalence of the toxin-antitoxin systems across archaea [22]. This distribution, with a dominant presence in most major clades of both group-I and group-II bacteria, suggests that polymorphic toxin systems could have been present in the ancestral bacterium." --- First, what is meant by "this distribution"? My understanding is that "this distribution" includes "general rarity" of polymorphic toxins in archaea. How can rarity of a system in archaea suggest its presence in bacterial stem, as opposed to later invention in bacteria? I suspect that this is mostly unfortunate wording that should be edited. In contrast, my second concern is more fundamental: essentially, any phyletic distribution may be interpreted as 1. ancestral presence of a gene followed by gene losses, or 2. later invention in one clade followed by horizontal transfers to to the other clades; or else 3. some combination of ancestral presence, losses and HGT. To turn these scenarios from mere hand waving to something supported by the evidence, one has to specify the model of gene gain and gene loss more explicitly, or to bring in some auxiliary evidence that favors one of the explanations. I do not see much of this here.

***Authors’ response****: We agree that this section was a bit unclear and we have now revised it. Similar to the previous point, the polymorphic toxin systems that we report in this study are present in all major lineages of bacteria. While there is no denial that extensive lateral transfer of these systems occurs, the presence in the ancestral bacterium with divergence mirroring the evolution of different secretion systems within the bacterial superkingdom is a parsimonious argument. In contrast only a few archaeal “species” contain these systems suggesting that they were probably not present in the ancestral archaeon. Parsimoniously, this suggests that the few archaeal polymorphic toxin systems were acquired from bacterial versions, because alternatively it would require a large number of gene losses in different archaeal linaeges.*

***Referee’s further response***: *In the previous exchange, the presence of a gene at the root of group I only, but not at the root of group II nor at joint root of I + II, was called “parsimonious”. Now, presence at the root of all bacteria is believed to be parsimonious, when the same set of taxa is examined. What kind of parsimony is invoked in each case? (I think I can discern the answer from the next two sentences, but please correct me if I am wrong). The authors appear to understand parsimony as the explanation that requires the smaller number of events. I cannot accept this as an always-preferable explanation, when it does not matter what these events are and how are they counted; in a moderate form, however, we can use parsimony as a criterion of selecting the null hypothesis,* i.e.*, “choose the scenario with the smallest number of events, unless the additional evidence suggests that a more complex scenario has to be considered”. I think that, in this case, however, precisely such additional evidence is available in the form of evolutionary estimates of the relative rate of gene gain and gene loss: almost every estimate suggests that on average gene losses are moderately to highly more frequent than gene gains. So, unweighted parsimony will not work in these cases – a scenario with 1:1 gain-to-loss ratio will be actually making an additional assumption of a relative loss rate that is constrained to be lower than what is observed in nature. Everything is then hanging on the word “large” – how large the excess of losses in archaea is, so that this makes the scenario so unlikely?*

***Authors’ response***: *We agree that the general frequencies of gene loss tend to exceed those of gains. However, with respect to the toxin systems in archaea we are dealing with the following situation: The non-redundant database has representatives from over 225 completely sequenced WGS sequences. Classical polymorphic toxin-like systems are found only in about 15 of them. Thus, there are approximately 15 times the archaeal genomes which lack these as those which have these systems. Approximately more 1/3*^*rd*^*of the bacterial genomes have at least one such system. Hence, although the referee is right in pointing to the differences in the rates of loss exceeding gain, we believe our original reasoning based on the parsimony argument is a valid one.*

**Referee’s further response**:

This is also supported in phylogenetic trees, where the archaeal toxins or immunity domains group with particular bacterial versions.

Is this true for the trees of all families, or only some?

***Authors’ response***: *Baring the barnases where the relationship is difficult to ascertain one way or another, consistently the other toxin domains shows the archaeal branches embedded within the bacterial radiation.*

p. 53, the following sentence: "However, it should be noted that these genes and cassettes are highly prone to lateral transfer as suggested by the sporadic phyletic distribution of both toxin domains and immunity proteins [17]. Hence, the distribution of these systems might also reflect in part the secondary dispersion of such systems across diverse bacteria by lateral transfer." --- Essentially, this is the same as to say that inheritance of any genetic element may be either vertical or horizontal. So?

***Authors’ response****: While the sentence might on the surface appear trivial but needs to be seen in light of the earlier comment on the polymorphic toxins being inferred present in the stem of the bacterial superkingdom. While that inference can be made based on the distribution of the toxins and their corresponding secretion systems, we intended to provide a more realistic picture (the above sentences), lest it be taken that their evolutionary history was predominantly vertical since their emergence early in bacterial evolution.*

***Referee’s further response****: Once again, in the exchange regarding the statement on p. 46, the inference was that certain toxin was present in the step of proteobacteria + Bactoroidetes, but not in the stem of all bacteria. I suppose the scenarios are really different for different toxins – can this be made more explicit?*

***Authors’ response***: *The toxin distributions in bacteria are certainly affected by lateral transfer so we cannot be certain of the inference of particular toxin in the common ancestor. Nevertheless, based on the differential distributions, we can tentatively propose that some of the widespread versions, such as the barnase, HNH and deaminase domain toxins might have been present in the stems of the major bacterial clades such as those uniting the group-I bacteria or group-II bacteria.*

p. 53: "Certain patterns of distribution of polymorphic toxin systems appear to transcend phyletic boundaries… 1) the hyperthermophiles, which are often chemoautotrophs, from both bacteria and archaea show a strong tendency to lack such systems." --- this seems to be the case of multiple losses in bacteria, possibly favored by similarity in the habitats, and possibly ancestral absence in archaea. Ecological adaptations like this 'transcend phyletic boundaries' more or less by definition - is this the point?

***Authors’ response****: While adaptations directly related to an ecological niche are indeed obvious in terms of transcending phyletic boundaries, this is not necessarily the case with inter-organismal conflict systems, which do not directly relate to the ecological niche. Since we nevertheless found correlations between these systems and ecology, we felt it would be useful to point them out. This would help understanding the more subtle effects of ecology of a species on their interactions with conspecifics and other organisms.*

***Referee’s further response***: *The correlation has been observed between hyperthermophily and lack of polymorphic toxins. As the authors imply, this may in fact be the correlation between chemoautotrophy and lack of toxins – or is it? Which effects here are gross, and which are subtle? Could it be, for example, that hyperthermophily is generally correlated with reduced repertoire of all kinds of secreted proteins, which would be more easily destabilized and inactivated by adverse environment outside the cell?*

***Authors’ response***: *We agree that the point raised by the referee regarding temperature affecting protein stability and thereby placing a selective constraint on the number of toxins could be in principle a valid alternative explanation. However, beyond certain compositional and length distribution differences the total number of secreted and membrane proteins in hyperthermophiles do not appear to be significantly different from other organisms (*e.g. *Nilson et al. Proteins. 2005 Sep 1;60(4):606–16.) Hence, we are not certain if this explanation might be more relevant than autotrophy, which additionally accounts for the comparable situation in photosynthetic autotrophs.*

p. 56: in the case of oral microbiomes, I am not sure how some species were assigned to 'biofilm-forming' category and others to 'cheaters' - I think that at least some species in the latter category are biofilm-forming in their own right.

***Authors’ response****: As pure cultures, all these species are likely to form biofilms, but the oral environment is a mixed population of diverse bacterial species, and it is well known that oral biofilms are comprised of mixed bacterial species (Paster BJ* et al. *Bacterial diversity in human subgingival plaque, ref 198). In this context, we hypothesize that the number of toxin and immunity domains predicts how a species will interact with another one during the formation of a mixed biofilm.*

Reviewer 3: Dr Frank Eisenhaber (Bioinformatics Institute, Singapore)

I agreed to be a reviewer when reading the author list only to find out that MS is by far the longest that I have ever seen as reviewer in my life. Despite of the initial horror and of the impressive length, the text is a fine reading - both as a research paper and as a review of this specific field. One would not think to shorten it by a page. The thoughts and results are plausible (there is no hope to repeat the calculations even partially). There is considerable care for the detail throughout the text, figures and additional files (except for very minor things such as ref. 144 appearing incomplete).

I find the generous addition of supplementary information especially notable.

Possibly, this will be of greatest benefit for people creating annotation pipelines and sequence databases. For practical purposes, the authors might think to add archives with all the individual alignments in single files and domain models in several formats such as the HMMR2, HMMER3, etc. ready made.

I think that the work is a welcome addition to the scientific literature.

***Authors’ response****: We thank the reviewer for his positive comments and suggestions. A more user-friendly supplementary file is now provided with the alignments of the toxins and immunity domains as separate files in a zipped format. We will additionally upload all alignments to protein domain databases such as Pfam, so that researchers can access them more easily. Ref. 144 has been updated in the revision.*

## Supplementary Material

Additional file 1 Polymorphic toxin systems: comprehensive characterization of trafficking modes, processing, mechanisms, immunity and ecology using comparative genomics. Click here for file

## References

[B1] Rochat H Martin-Eauclaire H Animal toxins: facts and protocols 2000 Basel Boston: Birkhauser Verlag

[B2] Keeler RF Tu AT Toxicology of plant and fungal compounds 1991 New York: Dekker

[B3] Mackessy SP Handbook of venoms and toxins of reptiles 2010 Boca Raton: CRC Press

[B4] Alouf JE Popoff MR The comprehensive sourcebook of bacterial protein toxins 2006 3 Amsterdam; Boston: Elsevier Academic Press

[B5] Walsh C Antibiotics: actions, origins, resistance 2003 Washington, D.C.: ASM Press

[B6] Proft T Microbial toxins: molecular and cellular biology 2005 Norfolk, England: BIOS Scientific

[B7] Rappuoli R Montecucco C Guidebook to protein toxins and their use in cell biology 1997 Oxford; New York: Oxford University Press

[B8] Dhananjaya BL CJ DS: An overview on nucleases (DNase, RNase, and phosphodiesterase) in snake venoms Biochemistry (Mosc) 2010 75 1 1 6 10.1134/S0006297910010013 20331418

[B9] Endo Y Tsurugi K Mechanism of action of ricin and related toxic lectins on eukaryotic ribosomes Nucleic Acids Symp Ser 1986 17 187 190 3562265

[B10] Chakrabarti A Jha BK Silverman RH New insights into the role of RNase L in innate immunity J Interferon Cytokine Res 2011 31 1 49 57 10.1089/jir.2010.0120 21190483 PMC3021357

[B11] Wiesner J Vilcinskas A Antimicrobial peptides: the ancient arm of the human immune system Virulence 2010 1 5 440 464 10.4161/viru.1.5.12983 21178486

[B12] Li WM Barnes T Lee CH Endoribonucleases–enzymes gaining spotlight in mRNA metabolism FEBS J 2010 277 3 627 641 10.1111/j.1742-4658.2009.07488.x 19968858

[B13] Rosenberg HF RNase A ribonucleases and host defense: an evolving story J Leukoc Biol 2008 83 5 1079 1087 10.1189/jlb.1107725 18211964 PMC2692241

[B14] Merritt EA Hol WG AB5 toxins Curr Opin Struct Biol 1995 5 2 165 171 10.1016/0959-440X(95)80071-9 7648317

[B15] Russell AB Hood RD Bui NK LeRoux M Vollmer W Mougous JD Type VI secretion delivers bacteriolytic effectors to target cells Nature 2011 475 7356 343 347 10.1038/nature10244 21776080 PMC3146020

[B16] Aoki SK Poole SJ Hayes CS Low DA Toxin on a stick: modular CDI toxin delivery systems play roles in bacterial competition Virulence 2011 2 4 356 359 10.4161/viru.2.4.16463 21705856 PMC3173679

[B17] Zhang D Iyer LM Aravind L A novel immunity system for bacterial nucleic acid degrading toxins and its recruitment in various eukaryotic and DNA viral systems Nucleic Acids Res 2011 39 11 4532 4552 10.1093/nar/gkr036 21306995 PMC3113570

[B18] Iyer LM Zhang D Rogozin IB Aravind L Evolution of the deaminase fold and multiple origins of eukaryotic editing and mutagenic nucleic acid deaminases from bacterial toxin systems Nucleic Acids Res 2011 39 22 9473 9497 10.1093/nar/gkr691 21890906 PMC3239186

[B19] Sisto A Cipriani MG Morea M Lonigro SL Valerio F Lavermicocca P An Rhs-like genetic element is involved in bacteriocin production by Pseudomonas savastanoi pv. savastanoi Antonie Van Leeuwenhoek 2010 98 4 505 517 10.1007/s10482-010-9468-7 20563849

[B20] Cascales E Buchanan SK Duche D Kleanthous C Lloubes R Postle K Riley M Slatin S Cavard D Colicin biology Microbiol Mol Biol Rev 2007 71 1 158 229 10.1128/MMBR.00036-06 17347522 PMC1847374

[B21] Kobayashi I Behavior of restriction-modification systems as selfish mobile elements and their impact on genome evolution Nucleic Acids Res 2001 29 18 3742 3756 10.1093/nar/29.18.3742 11557807 PMC55917

[B22] Anantharaman V Aravind L New connections in the prokaryotic toxin-antitoxin network: relationship with the eukaryotic nonsense-mediated RNA decay system Genome Biol 2003 4 12 R81 10.1186/gb-2003-4-12-r81 14659018 PMC329420

[B23] Engelberg-Kulka H Glaser G Addiction modules and programmed cell death and antideath in bacterial cultures Annu Rev Microbiol 1999 53 43 70 10.1146/annurev.micro.53.1.43 10547685

[B24] Van Melderen L Toxin-antitoxin systems: why so many, what for? Curr Opin Microbiol 2010 13 6 781 785 10.1016/j.mib.2010.10.006 21041110

[B25] Aepfelbacher M Aktories K Just I Bacterial protein toxins 2000 Berlin; New York: Springer

[B26] Nguyen VT Kamio Y Cooperative assembly of beta-barrel pore-forming toxins J Biochem 2004 136 5 563 567 10.1093/jb/mvh160 15632294

[B27] Gilbert RJ Pore-forming toxins Cell Mol Life Sci 2002 59 5 832 844 10.1007/s00018-002-8471-1 12088283 PMC11146115

[B28] Leplae R Geeraerts D Hallez R Guglielmini J Dreze P Van Melderen L Diversity of bacterial type II toxin-antitoxin systems: a comprehensive search and functional analysis of novel families Nucleic Acids Res 2011 39 13 5513 5525 10.1093/nar/gkr131 21422074 PMC3141249

[B29] MacIntyre DL Miyata ST Kitaoka M Pukatzki S The Vibrio cholerae type VI secretion system displays antimicrobial properties Proc Natl Acad Sci USA 2010 107 45 19520 19524 10.1073/pnas.1012931107 20974937 PMC2984155

[B30] Schwarz S West TE Boyer F Chiang WC Carl MA Hood RD Rohmer L Tolker-Nielsen T Skerrett SJ Mougous JD Burkholderia type VI secretion systems have distinct roles in eukaryotic and bacterial cell interactions PLoS Pathog 2010 6 8 e1001068 10.1371/journal.ppat.1001068 20865170 PMC2928800

[B31] Linhartova I Bumba L Masin J Basler M Osicka R Kamanova J Prochazkova K Adkins I Hejnova-Holubova J Sadilkova L RTX proteins: a highly diverse family secreted by a common mechanism FEMS Microbiol Rev 2010 34 6 1076 1112 20528947 10.1111/j.1574-6976.2010.00231.x PMC3034196

[B32] Holberger LE Garza-Sanchez F Lamoureux J Low DA Hayes CS A novel family of toxin/antitoxin proteins in Bacillus species FEBS Lett 2012 586 2 132 136 10.1016/j.febslet.2011.12.020 22200572 PMC3259279

[B33] Iyer LM Makarova KS Koonin EV Aravind L Comparative genomics of the FtsK-HerA superfamily of pumping ATPases: implications for the origins of chromosome segregation, cell division and viral capsid packaging Nucleic Acids Res 2004 32 17 5260 5279 10.1093/nar/gkh828 15466593 PMC521647

[B34] Alvarez-Martinez CE Christie PJ Biological diversity of prokaryotic type IV secretion systems Microbiol Mol Biol Rev 2009 73 4 775 808 10.1128/MMBR.00023-09 19946141 PMC2786583

[B35] Cornelis GR The type III secretion injectisome Nat Rev Microbiol 2006 4 11 811 825 10.1038/nrmicro1526 17041629

[B36] Hayes CS Aoki SK Low DA Bacterial contact-dependent delivery systems Annu Rev Genet 2010 44 71 90 10.1146/annurev.genet.42.110807.091449 21047256

[B37] Delattre AS Clantin B Saint N Locht C Villeret V Jacob-Dubuisson F Functional importance of a conserved sequence motif in FhaC, a prototypic member of the TpsB/Omp85 superfamily FEBS J 2010 277 22 4755 4765 10.1111/j.1742-4658.2010.07881.x 20955520

[B38] Bonemann G Pietrosiuk A Mogk A Tubules and donuts: a type VI secretion story Mol Microbiol 2010 76 4 815 821 10.1111/j.1365-2958.2010.07171.x 20444095

[B39] Basler M Pilhofer M Henderson GP Jensen GJ Mekalanos JJ Type VI secretion requires a dynamic contractile phage tail-like structure Nature 2012 483 7388 182 186 10.1038/nature10846 22367545 PMC3527127

[B40] Yang G Dowling AJ Gerike U ffrench-Constant RH Waterfield NR Photorhabdus virulence cassettes confer injectable insecticidal activity against the wax moth J Bacteriol 2006 188 6 2254 2261 10.1128/JB.188.6.2254-2261.2006 16513755 PMC1428146

[B41] Hurst MR Glare TR Jackson TA Cloning Serratia entomophila antifeeding genes–a putative defective prophage active against the grass grub Costelytra zealandica J Bacteriol 2004 186 15 5116 5128 10.1128/JB.186.15.5116-5128.2004 15262948 PMC451664

[B42] Bowen D Rocheleau TA Blackburn M Andreev O Golubeva E Bhartia R ffrench-Constant RH Insecticidal toxins from the bacterium Photorhabdus luminescens Science 1998 280 5372 2129 2132 10.1126/science.280.5372.2129 9641921

[B43] Ellermeier CD Losick R Evidence for a novel protease governing regulated intramembrane proteolysis and resistance to antimicrobial peptides in Bacillus subtilis Genes Dev 2006 20 14 1911 1922 10.1101/gad.1440606 16816000 PMC1522089

[B44] Aoki SK Diner EJ de Roodenbeke CT Burgess BR Poole SJ Braaten BA Jones AM Webb JS Hayes CS Cotter PA A widespread family of polymorphic contact-dependent toxin delivery systems in bacteria Nature 2010 468 7322 439 442 10.1038/nature09490 21085179 PMC3058911

[B45] Jackson AP Thomas GH Parkhill J Thomson NR Evolutionary diversification of an ancient gene family (rhs) through C-terminal displacement BMC Genomics 2009 10 584 10.1186/1471-2164-10-584 19968874 PMC2935791

[B46] Kung VL Khare S Stehlik C Bacon EM Hughes AJ Hauser AR An rhs gene of Pseudomonas aeruginosa encodes a virulence protein that activates the inflammasome Proc Natl Acad Sci USA 2012 109 4 1275 1280 10.1073/pnas.1109285109 22232685 PMC3268321

[B47] Yongqing T Potempa J Pike RN Wijeyewickrema LC The lysine-specific gingipain of Porphyromonas gingivalis: importance to pathogenicity and potential strategies for inhibition Adv Exp Med Biol 2011 712 15 29 10.1007/978-1-4419-8414-2_2 21660656

[B48] Tonello F Montecucco C The anthrax lethal factor and its MAPK kinase-specific metalloprotease activity Mol Aspects Med 2009 30 6 431 438 10.1016/j.mam.2009.07.006 19665472

[B49] Sheahan KL Cordero CL Satchell KJ Autoprocessing of the Vibrio cholerae RTX toxin by the cysteine protease domain EMBO J 2007 26 10 2552 2561 10.1038/sj.emboj.7601700 17464284 PMC1868911

[B50] Shao F Merritt PM Bao Z Innes RW Dixon JE A Yersinia effector and a Pseudomonas avirulence protein define a family of cysteine proteases functioning in bacterial pathogenesis Cell 2002 109 5 575 588 10.1016/S0092-8674(02)00766-3 12062101

[B51] Rossetto O de Bernard M Pellizzari R Vitale G Caccin P Schiavo G Montecucco C Bacterial toxins with intracellular protease activity Clin Chim Acta 2000 291 2 189 199 10.1016/S0009-8981(99)00228-4 10675723

[B52] Pei J Grishin NV Prediction of a caspase-like fold in Tannerella forsythia virulence factor PrtH Cell Cycle 2009 8 9 1453 1455 10.4161/cc.8.9.8243 19305154

[B53] Makarova KS Aravind L Koonin EV A superfamily of archaeal, bacterial, and eukaryotic proteins homologous to animal transglutaminases Protein Sci 1999 8 8 1714 1719 10.1110/ps.8.8.1714 10452618 PMC2144420

[B54] Gordon VM Leppla SH Proteolytic activation of bacterial toxins: role of bacterial and host cell proteases Infect Immun 1994 62 2 333 340 8300195 10.1128/iai.62.2.333-340.1994 PMC186112

[B55] McNulty C Thompson J Barrett B Lord L Andersen C Roberts IS The cell surface expression of group 2 capsular polysaccharides in Escherichia coli: the role of KpsD, RhsA and a multi-protein complex at the pole of the cell Mol Microbiol 2006 59 3 907 922 10.1111/j.1365-2958.2005.05010.x 16420360

[B56] Hill CW Sandt CH Vlazny DA Rhs elements of Escherichia coli: a family of genetic composites each encoding a large mosaic protein Mol Microbiol 1994 12 6 865 871 10.1111/j.1365-2958.1994.tb01074.x 7934896

[B57] Lupardus PJ Shen A Bogyo M Garcia KC Small molecule-induced allosteric activation of the Vibrio cholerae RTX cysteine protease domain Science 2008 322 5899 265 268 10.1126/science.1162403 18845756 PMC3272704

[B58] Tinel A Janssens S Lippens S Cuenin S Logette E Jaccard B Quadroni M Tschopp J Autoproteolysis of PIDD marks the bifurcation between pro-death caspase-2 and pro-survival NF-kappaB pathway EMBO J 2007 26 1 197 208 10.1038/sj.emboj.7601473 17159900 PMC1782377

[B59] Janssens S Tinel A The PIDDosome, DNA-damage-induced apoptosis and beyond Cell Death Differ 2012 19 1 13 20 10.1038/cdd.2011.162 22095286 PMC3252840

[B60] Ponting CP Hofmann K Bork P A latrophilin/CL-1-like GPS domain in polycystin-1 Curr Biol 1999 9 16 R585 R588 10.1016/S0960-9822(99)80379-0 10469603

[B61] Mans BJ Anantharaman V Aravind L Koonin EV Comparative genomics, evolution and origins of the nuclear envelope and nuclear pore complex Cell Cycle 2004 3 12 1612 1637 10.4161/cc.3.12.1345 15611647

[B62] Hurst MR Glare TR Jackson TA Ronson CW Plasmid-located pathogenicity determinants of Serratia entomophila, the causal agent of amber disease of grass grub, show similarity to the insecticidal toxins of Photorhabdus luminescens J Bacteriol 2000 182 18 5127 5138 10.1128/JB.182.18.5127-5138.2000 10960097 PMC94661

[B63] Pei J Mitchell DA Dixon JE Grishin NV Expansion of type II CAAX proteases reveals evolutionary origin of gamma-secretase subunit APH-1 J Mol Biol 2011 410 1 18 26 10.1016/j.jmb.2011.04.066 21570408 PMC3155266

[B64] Frias M Gonzalez C Brito N BcSpl1, a cerato-platanin family protein, contributes to Botrytis cinerea virulence and elicits the hypersensitive response in the host New Phytol 2011 192 2 483 495 10.1111/j.1469-8137.2011.03802.x 21707620

[B65] Aravind L Iyer LM Anantharaman V Spiro S, Dixon R Natural history of sensor domains in bacterial signaling systems Sensory Mechanisms in Bacteria: Molecular Aspects of Signal Recognition 2010 Norfolk, UK: Caister Academic Press

[B66] Aravind L Koonin EV Classification of the caspase-hemoglobinase fold: detection of new families and implications for the origin of the eukaryotic separins Proteins 2002 46 4 355 367 10.1002/prot.10060 11835511

[B67] Barrett AJ Rawlings ND Evolutionary lines of cysteine peptidases Biol Chem 2001 382 5 727 733 11517925 10.1515/BC.2001.088

[B68] Kitadokoro K Kamitani S Miyazawa M Hanajima-Ozawa M Fukui A Miyake M Horiguchi Y Crystal structures reveal a thiol protease-like catalytic triad in the C-terminal region of Pasteurella multocida toxin Proc Natl Acad Sci USA 2007 104 12 5139 5144 10.1073/pnas.0608197104 17360394 PMC1829276

[B69] Zhu M Shao F Innes RW Dixon JE Xu Z The crystal structure of Pseudomonas avirulence protein AvrPphB: a papain-like fold with a distinct substrate-binding site Proc Natl Acad Sci USA 2004 101 1 302 307 10.1073/pnas.2036536100 14694194 PMC314180

[B70] Kagawa TF Cooney JC Baker HM McSweeney S Liu M Gubba S Musser JM Baker EN Crystal structure of the zymogen form of the group A Streptococcus virulence factor SpeB: an integrin-binding cysteine protease Proc Natl Acad Sci USA 2000 97 5 2235 2240 10.1073/pnas.040549997 10681429 PMC15784

[B71] Anantharaman V Aravind L Evolutionary history, structural features and biochemical diversity of the NlpC/P60 superfamily of enzymes Genome Biol 2003 4 2 R11 10.1186/gb-2003-4-2-r11 12620121 PMC151301

[B72] Pei J Grishin NV The Rho GTPase inactivation domain in Vibrio cholerae MARTX toxin has a circularly permuted papain-like thiol protease fold Proteins: Structure, Function, and Bioinformatics 2009 77 2 413 419 10.1002/prot.22447 PMC3688474 19434753

[B73] Wood MW Williams C Upadhyay A Gill AC Philippe DL Galyov EE van den Elsen JM Bagby S Structural analysis of Salmonella enterica effector protein SopD Biochim Biophys Acta 2004 1698 2 219 226 10.1016/j.bbapap.2003.12.003 15134655

[B74] Richards GP Watson MA Crane EJ Burt IG Bushek D Shewanella and Photobacterium spp. in oysters and seawater from the Delaware Bay Appl Environ Microbiol 2008 74 11 3323 3327 10.1128/AEM.00060-08 18378645 PMC2423050

[B75] Burroughs AM Iyer LM Aravind L Comparative genomics and evolutionary trajectories of viral ATP dependent DNA-packaging systems Genome Dyn 2007 3 48 65 18753784 10.1159/000107603

[B76] Nanao MH Tcherniuk SO Chroboczek J Dideberg O Dessen A Balakirev MY Crystal structure of human otubain 2 EMBO Rep 2004 5 8 783 788 10.1038/sj.embor.7400201 15258613 PMC1299112

[B77] Wertz IE O'Rourke KM Zhou H Eby M Aravind L Seshagiri S Wu P Wiesmann C Baker R Boone DL De-ubiquitination and ubiquitin ligase domains of A20 downregulate NF-kappaB signalling Nature 2004 430 7000 694 699 10.1038/nature02794 15258597

[B78] Birtles RJ Rowbotham TJ Michel R Pitcher DG Lascola B Alexiou-Daniel S Raoult D Candidatus Odyssella thessalonicensis' gen. nov., sp. nov., an obligate intracellular parasite of Acanthamoeba species Int J Syst Evol Microbiol 2000 50 Pt 1 63 72 10826788 10.1099/00207713-50-1-63

[B79] Schmitz-Esser S Tischler P Arnold R Montanaro J Wagner M Rattei T Horn M The genome of the amoeba symbiont "Candidatus Amoebophilus asiaticus" reveals common mechanisms for host cell interaction among amoeba-associated bacteria J Bacteriol 2010 192 4 1045 1057 10.1128/JB.01379-09 20023027 PMC2812958

[B80] Loureiro J Ploegh HL Antigen presentation and the ubiquitin-proteasome system in host-pathogen interactions Adv Immunol 2006 92 225 305 17145306 10.1016/S0065-2776(06)92006-9 PMC7112114

[B81] Iyer LM Leipe DD Koonin EV Aravind L Evolutionary history and higher order classification of AAA + ATPases J Struct Biol 2004 146 1–2 11 31 15037234 10.1016/j.jsb.2003.10.010

[B82] Bonemann G Pietrosiuk A Diemand A Zentgraf H Mogk A Remodelling of VipA/VipB tubules by ClpV-mediated threading is crucial for type VI protein secretion EMBO J 2009 28 4 315 325 10.1038/emboj.2008.269 19131969 PMC2646146

[B83] Dhanaraj V Ye QZ Johnson LL Hupe DJ Ortwine DF Dunbar JB Rubin JR Pavlovsky A Humblet C Blundell TL X-ray structure of a hydroxamate inhibitor complex of stromelysin catalytic domain and its comparison with members of the zinc metalloproteinase superfamily Structure 1996 4 4 375 386 10.1016/S0969-2126(96)00043-3 8740360

[B84] ffrench-Constant RH Dowling A Waterfield NR Insecticidal toxins from Photorhabdus bacteria and their potential use in agriculture Toxicon 2007 49 4 436 451 10.1016/j.toxicon.2006.11.019 17207509

[B85] Pechy-Tarr M Bruck DJ Maurhofer M Fischer E Vogne C Henkels MD Donahue KM Grunder J Loper JE Keel C Molecular analysis of a novel gene cluster encoding an insect toxin in plant-associated strains of Pseudomonas fluorescens Environ Microbiol 2008 10 9 2368 2386 10.1111/j.1462-2920.2008.01662.x 18484997

[B86] Rodou A Ankrah DO Stathopoulos C Toxins and Secretion Systems of Photorhabdus luminescens Toxins (Basel) 2010 2 6 1250 1264 22069636 10.3390/toxins2061250 PMC3153242

[B87] Daborn PJ Waterfield N Silva CP Au CP Sharma S ffrench-Constant RH A single Photorhabdus gene, makes caterpillars floppy (mcf), allows Escherichia coli to persist within and kill insects Proc Natl Acad Sci USA 2002 99 16 10742 10747 10.1073/pnas.102068099 12136122 PMC125031

[B88] Wei CF Kvitko BH Shimizu R Crabill E Alfano JR Lin NC Martin GB Huang HC Collmer A A Pseudomonas syringae pv. tomato DC3000 mutant lacking the type III effector HopQ1–1 is able to cause disease in the model plant Nicotiana benthamiana Plant J 2007 51 1 32 46 10.1111/j.1365-313X.2007.03126.x 17559511

[B89] Li X Lin H Zhang W Zou Y Zhang J Tang X Zhou JM Flagellin induces innate immunity in nonhost interactions that is suppressed by Pseudomonas syringae effectors Proc Natl Acad Sci USA 2005 102 36 12990 12995 10.1073/pnas.0502425102 16123135 PMC1200263

[B90] Masuda M Betancourt L Matsuzawa T Kashimoto T Takao T Shimonishi Y Horiguchi Y Activation of rho through a cross-link with polyamines catalyzed by Bordetella dermonecrotizing toxin EMBO J 2000 19 4 521 530 10.1093/emboj/19.4.521 10675321 PMC305590

[B91] Dirix G Monsieurs P Dombrecht B Daniels R Marchal K Vanderleyden J Michiels J Peptide signal molecules and bacteriocins in Gram-negative bacteria: a genome-wide in silico screening for peptides containing a double-glycine leader sequence and their cognate transporters Peptides 2004 25 9 1425 1440 10.1016/j.peptides.2003.10.028 15374646

[B92] Ishii S Yano T Ebihara A Okamoto A Manzoku M Hayashi H Crystal structure of the peptidase domain of Streptococcus ComA, a bifunctional ATP-binding cassette transporter involved in the quorum-sensing pathway J Biol Chem 2010 285 14 10777 10785 10.1074/jbc.M109.093781 20100826 PMC2856284

[B93] Kelly M Hart E Mundy R Marches O Wiles S Badea L Luck S Tauschek M Frankel G Robins-Browne RM Essential role of the type III secretion system effector NleB in colonization of mice by Citrobacter rodentium Infect Immun 2006 74 4 2328 2337 10.1128/IAI.74.4.2328-2337.2006 16552063 PMC1418941

[B94] Wong AR Pearson JS Bright MD Munera D Robinson KS Lee SF Frankel G Hartland EL Enteropathogenic and enterohaemorrhagic Escherichia coli: even more subversive elements Mol Microbiol 2011 80 6 1420 1438 10.1111/j.1365-2958.2011.07661.x 21488979

[B95] Iyer LM Koonin EV Aravind L Novel predicted peptidases with a potential role in the ubiquitin signaling pathway Cell Cycle 2004 3 11 1440 1450 10.4161/cc.3.11.1206 15483401

[B96] Odagaki Y Hayashi A Okada K Hirotsu K Kabashima T Ito K Yoshimoto T Tsuru D Sato M Clardy J The crystal structure of pyroglutamyl peptidase I from Bacillus amyloliquefaciens reveals a new structure for a cysteine protease Structure 1999 7 4 399 411 10.1016/S0969-2126(99)80053-7 10196127

[B97] Takamatsu H Imamura A Kodama T Asai K Ogasawara N Watabe K The yabG gene of Bacillus subtilis encodes a sporulation specific protease which is involved in the processing of several spore coat proteins FEMS Microbiol Lett 2000 192 1 33 38 10.1111/j.1574-6968.2000.tb09355.x 11040425

[B98] Biarrotte-Sorin S Hugonnet JE Delfosse V Mainardi JL Gutmann L Arthur M Mayer C Crystal structure of a novel beta-lactam-insensitive peptidoglycan transpeptidase J Mol Biol 2006 359 3 533 538 10.1016/j.jmb.2006.03.014 16647082

[B99] Bielnicki J Devedjiev Y Derewenda U Dauter Z Joachimiak A Derewenda ZS B. subtilis ykuD protein at 2.0 A resolution: insights into the structure and function of a novel, ubiquitous family of bacterial enzymes Proteins 2006 62 1 144 151 16287140 10.1002/prot.20702 PMC2792008

[B100] Degnan PH Moran NA Diverse phage-encoded toxins in a protective insect endosymbiont Appl Environ Microbiol 2008 74 21 6782 6791 10.1128/AEM.01285-08 18791000 PMC2576707

[B101] Oliver KM Degnan PH Hunter MS Moran NA Bacteriophages encode factors required for protection in a symbiotic mutualism Science 2009 325 5943 992 994 10.1126/science.1174463 19696350 PMC5473335

[B102] Aravind L Walker DR Koonin EV Conserved domains in DNA repair proteins and evolution of repair systems Nucleic Acids Res 1999 27 5 1223 1242 10.1093/nar/27.5.1223 9973609 PMC148307

[B103] Aravind L Makarova KS Koonin EV Holliday junction resolvases and related nucleases: identification of new families, phyletic distribution and evolutionary trajectories Nucleic Acids Res 2000 28 18 3417 3432 10.1093/nar/28.18.3417 10982859 PMC110722

[B104] Mak AN Lambert AR Stoddard BL Folding, DNA recognition, and function of GIY-YIG endonucleases: crystal structures of R. Eco29kI Structure 2010 18 10 1321 1331 10.1016/j.str.2010.07.006 20800503 PMC2955809

[B105] Zhao L Bonocora RP Shub DA Stoddard BL The restriction fold turns to the dark side: a bacterial homing endonuclease with a PD-(D/E)-XK motif EMBO J 2007 26 9 2432 2442 10.1038/sj.emboj.7601672 17410205 PMC1864971

[B106] Stoddard BL Homing endonuclease structure and function Q Rev Biophys 2005 38 1 49 95 16336743 10.1017/S0033583505004063

[B107] Yang W Nucleases: diversity of structure, function and mechanism Q Rev Biophys 2011 44 1 1 93 10.1017/S0033583510000181 20854710 PMC6320257

[B108] Anantharaman V Aravind L The NYN domains: novel predicted RNAses with a PIN domain-like fold RNA Biol 2006 3 1 18 27 10.4161/rna.3.1.2548 17114934

[B109] Carr S Walker D James R Kleanthous C Hemmings AM Inhibition of a ribosome-inactivating ribonuclease: the crystal structure of the cytotoxic domain of colicin E3 in complex with its immunity protein Structure 2000 8 9 949 960 10.1016/S0969-2126(00)00186-6 10986462

[B110] Graille M Mora L Buckingham RH van Tilbeurgh H de Zamaroczy M Structural inhibition of the colicin D tRNase by the tRNA-mimicking immunity protein EMBO J 2004 23 7 1474 1482 10.1038/sj.emboj.7600162 15014439 PMC391069

[B111] Ghosh M Meiss G Pingoud A London RE Pedersen LC Structural insights into the mechanism of nuclease A, a betabeta alpha metal nuclease from Anabaena J Biol Chem 2005 280 30 27990 27997 10.1074/jbc.M501798200 15897201

[B112] Guthrie EP Quinton-Jager T Moran LS Slatko BE Kucera RB Benner JS Wilson GG Brooks JE Cloning, expression and sequence analysis of the SphI restriction-modification system Gene 1996 180 1–2 107 112 8973353 10.1016/s0378-1119(96)00415-5

[B113] Woo EJ Kim YG Kim MS Han WD Shin S Robinson H Park SY Oh BH Structural mechanism for inactivation and activation of CAD/DFF40 in the apoptotic pathway Mol Cell 2004 14 4 531 539 10.1016/S1097-2765(04)00258-8 15149602

[B114] Lugovskoy AA Zhou P Chou JJ McCarty JS Li P Wagner G Solution structure of the CIDE-N domain of CIDE-B and a model for CIDE-N/CIDE-N interactions in the DNA fragmentation pathway of apoptosis Cell 1999 99 7 747 755 10.1016/S0092-8674(00)81672-4 10619428

[B115] Minet AD Rubin BP Tucker RP Baumgartner S Chiquet-Ehrismann R Teneurin-1, a vertebrate homologue of the Drosophila pair-rule gene ten-m, is a neuronal protein with a novel type of heparin-binding domain J Cell Sci 1999 112 Pt 12 2019 2032 10341219 10.1242/jcs.112.12.2019

[B116] Silva JP Lelianova VG Ermolyuk YS Vysokov N Hitchen PG Berninghausen O Rahman MA Zangrandi A Fidalgo S Tonevitsky AG Latrophilin 1 and its endogenous ligand Lasso/teneurin-2 form a high-affinity transsynaptic receptor pair with signaling capabilities Proc Natl Acad Sci USA 2011 108 29 12113 12118 10.1073/pnas.1019434108 21724987 PMC3141932

[B117] Topf U Chiquet-Ehrismann R Genetic interaction between Caenorhabditis elegans teneurin ten-1 and prolyl 4-hydroxylase phy-1 and their function in collagen IV-mediated basement membrane integrity during late elongation of the embryo Mol Biol Cell 2011 22 18 3331 3343 10.1091/mbc.E10-10-0853 21795395 PMC3172259

[B118] Qian X Barsyte-Lovejoy D Wang L Chewpoy B Gautam N Al Chawaf A Lovejoy DA Cloning and characterization of teneurin C-terminus associated peptide (TCAP)-3 from the hypothalamus of an adult rainbow trout (Oncorhynchus mykiss) Gen Comp Endocrinol 2004 137 2 205 216 10.1016/j.ygcen.2004.02.007 15158132

[B119] Aravind L Koonin EV Prokaryotic homologs of the eukaryotic DNA-end-binding protein Ku, novel domains in the Ku protein and prediction of a prokaryotic double-strand break repair system Genome Res 2001 11 8 1365 1374 10.1101/gr.181001 11483577 PMC311082

[B120] Aravind L Iyer LM The HARE-HTH and associated domains: novel modules in the coordination of epigenetic DNA and protein modifications Cell Cycle 2012 11 1 119 131 10.4161/cc.11.1.18475 22186017 PMC3272235

[B121] Vosman B Kuiken G Kooistra J Venema G Transformation in Bacillus subtilis: involvement of the 17-kilodalton DNA-entry nuclease and the competence-specific 18-kilodalton protein J Bacteriol 1988 170 8 3703 3710 2841296 10.1128/jb.170.8.3703-3710.1988 PMC211348

[B122] Johnson EP Mincer T Schwab H Burgin AB Helinski DR Plasmid RK2 ParB protein: purification and nuclease properties J Bacteriol 1999 181 19 6010 6018 10498713 10.1128/jb.181.19.6010-6018.1999 PMC103628

[B123] Jonsson TJ Murray MS Johnson LC Poole LB Lowther WT Structural basis for the retroreduction of inactivated peroxiredoxins by human sulfiredoxin Biochemistry 2005 44 24 8634 8642 10.1021/bi050131i 15952770 PMC3928543

[B124] Chen S Wang L Deng Z Twenty years hunting for sulfur in DNA Protein Cell 2010 1 1 14 21 10.1007/s13238-010-0009-y 21203994 PMC4875114

[B125] Iyer LM Tahiliani M Rao A Aravind L Prediction of novel families of enzymes involved in oxidative and other complex modifications of bases in nucleic acids Cell Cycle 2009 8 11 1698 1710 10.4161/cc.8.11.8580 19411852 PMC2995806

[B126] Burroughs AM Iyer LM Aravind L Functional diversification of the RING finger and other binuclear treble clef domains in prokaryotes and the early evolution of the ubiquitin system Mol Biosyst 2011 7 7 2261 2277 10.1039/c1mb05061c 21547297 PMC5938088

[B127] Iyer LM Burroughs AM Aravind L The prokaryotic antecedents of the ubiquitin-signaling system and the early evolution of ubiquitin-like beta-grasp domains Genome Biol 2006 7 7 R60 10.1186/gb-2006-7-7-r60 16859499 PMC1779556

[B128] Burns KE Baumgart S Dorrestein PC Zhai H McLafferty FW Begley TP Reconstitution of a new cysteine biosynthetic pathway in Mycobacterium tuberculosis J Am Chem Soc 2005 127 33 11602 11603 10.1021/ja053476x 16104727 PMC2536522

[B129] Sarkar S Iyer G Wu J Glass NL Nonself recognition is mediated by HET-C heterocomplex formation during vegetative incompatibility EMBO J 2002 21 18 4841 4850 10.1093/emboj/cdf479 12234924 PMC126278

[B130] Wichmann G Sun J Dementhon K Glass NL Lindow SE A novel gene, phcA from Pseudomonas syringae induces programmed cell death in the filamentous fungus Neurospora crassa Mol Microbiol 2008 68 3 672 689 10.1111/j.1365-2958.2008.06175.x 18363647

[B131] Hough E Hansen LK Birknes B Jynge K Hansen S Hordvik A Little C Dodson E Derewenda Z High-resolution (1.5 A) crystal structure of phospholipase C from Bacillus cereus Nature 1989 338 6213 357 360 10.1038/338357a0 2493587

[B132] Romier C Dominguez R Lahm A Dahl O Suck D Recognition of single-stranded DNA by nuclease P1: high resolution crystal structures of complexes with substrate analogs Proteins 1998 32 4 414 424 10.1002/(SICI)1097-0134(19980901)32:4<414::AID-PROT2>3.0.CO;2-G 9726413

[B133] Kawano M Aravind L Storz G An antisense RNA controls synthesis of an SOS-induced toxin evolved from an antitoxin Mol Microbiol 2007 64 3 738 754 10.1111/j.1365-2958.2007.05688.x 17462020 PMC1891008

[B134] Structural Classification of Proteinshttp://scop.mrc-lmb.cam.ac.uk/scop/index.html

[B135] Gioia U Laneve P Dlakic M Arceci M Bozzoni I Caffarelli E Functional characterization of XendoU, the endoribonuclease involved in small nucleolar RNA biosynthesis J Biol Chem 2005 280 19 18996 19002 10.1074/jbc.M501160200 15755742

[B136] Raines RT Ribonuclease A Chem Rev 1998 98 3 1045 1066 10.1021/cr960427h 11848924

[B137] Ng CL Lang K Meenan NA Sharma A Kelley AC Kleanthous C Ramakrishnan V Structural basis for 16 S ribosomal RNA cleavage by the cytotoxic domain of colicin E3 Nat Struct Mol Biol 2010 17 10 1241 1246 10.1038/nsmb.1896 20852642 PMC3755339

[B138] Duron O Insights beyond Wolbachia-Drosophila interactions: never completely trust a model: insights from cytoplasmic incompatibility beyond Wolbachia-Drosophila interactions Heredity (Edinb) 2008 101 6 473 474 10.1038/hdy.2008.113 18941470

[B139] Yarbrough ML Li Y Kinch LN Grishin NV Ball HL Orth K AMPylation of Rho GTPases by Vibrio VopS disrupts effector binding and downstream signaling Science 2009 323 5911 269 272 10.1126/science.1166382 19039103

[B140] Feng F Yang F Rong W Wu X Zhang J Chen S He C Zhou JM A Xanthomonas uridine 5'-monophosphate transferase inhibits plant immune kinases Nature 2012 485 7396 114 118 10.1038/nature10962 22504181

[B141] Goto Y Li B Claesen J Shi Y Bibb MJ van der Donk WA Discovery of unique lanthionine synthetases reveals new mechanistic and evolutionary insights PLoS Biol 2010 8 3 e1000339 10.1371/journal.pbio.1000339 20351769 PMC2843593

[B142] You YO Levengood MR Ihnken LA Knowlton AK van der Donk WA Lacticin 481 synthetase as a general serine/threonine kinase ACS Chem Biol 2009 4 5 379 385 10.1021/cb800309v 19292452 PMC2709986

[B143] Reinert DJ Jank T Aktories K Schulz GE Structural basis for the function of Clostridium difficile toxin B J Mol Biol 2005 351 5 973 981 10.1016/j.jmb.2005.06.071 16054646

[B144] Degnan PH Yu Y Sisneros N Wing RA Moran NA Hamiltonella defensa, genome evolution of protective bacterial endosymbiont from pathogenic ancestors Proc Natl Acad Sci USA 2009 106 22 9063 9068 10.1073/pnas.0900194106 19451630 PMC2690004

[B145] Fieldhouse RJ Turgeon Z White D Merrill AR Cholera- and anthrax-like toxins are among several new ADP-ribosyltransferases PLoS Comput Biol 2010 6 12 e1001029 10.1371/journal.pcbi.1001029 21170356 PMC3000352

[B146] Otto H Reche PA Bazan F Dittmar K Haag F Koch-Nolte F In silico characterization of the family of PARP-like poly(ADP-ribosyl)transferases (pARTs) BMC Genomics 2005 6 139 10.1186/1471-2164-6-139 16202152 PMC1266365

[B147] Bazan JF Koch-Nolte F Sequence and structural links between distant ADP-ribosyltransferase families Adv Exp Med Biol 1997 419 99 107 10.1007/978-1-4419-8632-0_12 9193642

[B148] de Souza RF Aravind L Identification of novel components of NAD-utilizing metabolic pathways and prediction of their biochemical functions Mol Biosyst 2012 8 6 1661 1677 10.1039/c2mb05487f 22399070

[B149] Jorgensen R Purdy AE Fieldhouse RJ Kimber MS Bartlett DH Merrill AR Cholix toxin, a novel ADP-ribosylating factor from Vibrio cholerae J Biol Chem 2008 283 16 10671 10678 10.1074/jbc.M710008200 18276581

[B150] Yates SP Jorgensen R Andersen GR Merrill AR Stealth and mimicry by deadly bacterial toxins Trends Biochem Sci 2006 31 2 123 133 10.1016/j.tibs.2005.12.007 16406634

[B151] Reinert DJ Carpusca I Aktories K Schulz GE Structure of the mosquitocidal toxin from Bacillus sphaericus J Mol Biol 2006 357 4 1226 1236 10.1016/j.jmb.2006.01.025 16483607

[B152] Hayashi S Ishii T Matsunaga T Tominaga R Kuromori T Wada T Shinozaki K Hirayama T The glycerophosphoryl diester phosphodiesterase-like proteins SHV3 and its homologs play important roles in cell wall organization Plant Cell Physiol 2008 49 10 1522 1535 10.1093/pcp/pcn120 18718934

[B153] Kang TS Georgieva D Genov N Murakami MT Sinha M Kumar RP Kaur P Kumar S Dey S Sharma S Enzymatic toxins from snake venom: structural characterization and mechanism of catalysis FEBS J 2011 278 23 4544 4576 10.1111/j.1742-4658.2011.08115.x 21470368

[B154] Sandoval-Calderon M Geiger O Guan Z Barona-Gomez F Sohlenkamp C A eukaryote-like cardiolipin synthase is present in Streptomyces coelicolor and in most actinobacteria J Biol Chem 2009 284 26 17383 17390 10.1074/jbc.M109.006072 19439403 PMC2719378

[B155] Dowhan W Molecular basis for membrane phospholipid diversity: why are there so many lipids? Annu Rev Biochem 1997 66 199 232 10.1146/annurev.biochem.66.1.199 9242906

[B156] Nambu T Minamino T Macnab RM Kutsukake K Peptidoglycan-hydrolyzing activity of the FlgJ protein, essential for flagellar rod formation in Salmonella typhimurium J Bacteriol 1999 181 5 1555 1561 10049388 10.1128/jb.181.5.1555-1561.1999 PMC93546

[B157] Henrissat B Callebaut I Fabrega S Lehn P Mornon JP Davies G Conserved catalytic machinery and the prediction of a common fold for several families of glycosyl hydrolases Proc Natl Acad Sci USA 1995 92 15 7090 7094 10.1073/pnas.92.15.7090 7624375 PMC41477

[B158] Copley RR Bork P Homology among (betaalpha)(8) barrels: implications for the evolution of metabolic pathways J Mol Biol 2000 303 4 627 641 10.1006/jmbi.2000.4152 11054297

[B159] Aravind L Koonin EV DNA polymerase beta-like nucleotidyltransferase superfamily: identification of three new families, classification and evolutionary history Nucleic Acids Res 1999 27 7 1609 1618 10.1093/nar/27.7.1609 10075991 PMC148363

[B160] Potrykus K Cashel M (p)ppGpp still magical? Annu Rev Microbiol 2008 62 35 51 10.1146/annurev.micro.62.081307.162903 18454629

[B161] Liu Q Kriksunov IA Graeff R Munshi C Lee HC Hao Q Crystal structure of human CD38 extracellular domain Structure 2005 13 9 1331 1339 10.1016/j.str.2005.05.012 16154090

[B162] Guse AH Lee HC NAADP: a universal Ca2+ trigger Sci Signal 2008 1 44 re10 10.1126/scisignal.144re10 18984909

[B163] Chini EN CD38 as a regulator of cellular NAD: a novel potential pharmacological target for metabolic conditions Curr Pharm Des 2009 15 1 57 63 10.2174/138161209787185788 19149603 PMC2883294

[B164] Iacovache I van der Goot FG Pernot L Pore formation: an ancient yet complex form of attack Biochim Biophys Acta 2008 1778 7–8 1611 1623 18298943 10.1016/j.bbamem.2008.01.026

[B165] Gonzalez MR Bischofberger M Pernot L van der Goot FG Freche B Bacterial pore-forming toxins: the (w)hole story? Cell Mol Life Sci 2008 65 3 493 507 10.1007/s00018-007-7434-y 17989920 PMC11131829

[B166] Rescher U Gerke V Annexins–unique membrane binding proteins with diverse functions J Cell Sci 2004 117 Pt 13 2631 2639 15169834 10.1242/jcs.01245

[B167] Rohou A Nield J Ushkaryov YA Insecticidal toxins from black widow spider venom Toxicon 2007 49 4 531 549 10.1016/j.toxicon.2006.11.021 17210168 PMC2517654

[B168] Dulubova IE Krasnoperov VG Khvotchev MV Pluzhnikov KA Volkova TM Grishin EV Vais H Bell DR Usherwood PN Cloning and structure of delta-latroinsectotoxin, a novel insect-specific member of the latrotoxin family: functional expression requires C-terminal truncation J Biol Chem 1996 271 13 7535 7543 10.1074/jbc.271.13.7535 8631785

[B169] King JG Vernick KD Hillyer JF Members of the salivary gland surface protein (SGS) family are major immunogenic components of mosquito saliva J Biol Chem 2011 286 47 40824 40834 10.1074/jbc.M111.280552 21965675 PMC3220476

[B170] Klasson L Kambris Z Cook PE Walker T Sinkins SP Horizontal gene transfer between Wolbachia and the mosquito Aedes aegypti BMC Genomics 2009 10 33 10.1186/1471-2164-10-33 19154594 PMC2647948

[B171] Aschtgen MS Gavioli M Dessen A Lloubes R Cascales E The SciZ protein anchors the enteroaggregative Escherichia coli Type VI secretion system to the cell wall Mol Microbiol 2010 75 4 886 899 10.1111/j.1365-2958.2009.07028.x 20487285

[B172] Parsons LM Lin F Orban J Peptidoglycan recognition by Pal, an outer membrane lipoprotein Biochemistry 2006 45 7 2122 2128 10.1021/bi052227i 16475801

[B173] Neumann U Schiltz E Stahl B Hillenkamp F Weckesser J A peptidoglycan binding domain in the porin-associated protein (PAP) of Rhodospirillum rubrum FR1 FEMS Microbiol Lett 1996 138 1 55 58 10.1111/j.1574-6968.1996.tb08134.x 8674970

[B174] Park JS Lee WC Yeo KJ Ryu KS Kumarasiri M Hesek D Lee M Mobashery S Song JH Kim SI Mechanism of anchoring of OmpA protein to the cell wall peptidoglycan of the gram-negative bacterial outer membrane FASEB J 2012 26 1 219 228 10.1096/fj.11-188425 21965596 PMC3250236

[B175] Babu MM Priya ML Selvan AT Madera M Gough J Aravind L Sankaran K A database of bacterial lipoproteins (DOLOP) with functional assignments to predicted lipoproteins J Bacteriol 2006 188 8 2761 2773 10.1128/JB.188.8.2761-2773.2006 16585737 PMC1446993

[B176] Leipe DD Koonin EV Aravind L STAND, a class of P-loop NTPases including animal and plant regulators of programmed cell death: multiple, complex domain architectures, unusual phyletic patterns, and evolution by horizontal gene transfer J Mol Biol 2004 343 1 1 28 10.1016/j.jmb.2004.08.023 15381417

[B177] Schwefel D Frohlich C Eichhorst J Wiesner B Behlke J Aravind L Daumke O Structural basis of oligomerization in septin-like GTPase of immunity-associated protein 2 (GIMAP2) Proc Natl Acad Sci USA 2010 107 47 20299 20304 10.1073/pnas.1010322107 21059949 PMC2996681

[B178] Velikovsky CA Deng L Tasumi S Iyer LM Kerzic MC Aravind L Pancer Z Mariuzza RA Structure of a lamprey variable lymphocyte receptor in complex with a protein antigen Nat Struct Mol Biol 2009 16 7 725 730 10.1038/nsmb.1619 19543291 PMC2722044

[B179] Wolf YI Rogozin IB Kondrashov AS Koonin EV Genome alignment, evolution of prokaryotic genome organization, and prediction of gene function using genomic context Genome Res 2001 11 3 356 372 10.1101/gr.GR-1619R 11230160

[B180] Koonin EV Wolf YI Aravind L Prediction of the archaeal exosome and its connections with the proteasome and the translation and transcription machineries by a comparative-genomic approach Genome Res 2001 11 2 240 252 10.1101/gr.162001 11157787 PMC311015

[B181] Poole SJ Diner EJ Aoki SK Braaten BA t'Kint de Roodenbeke C Low DA Hayes CS Identification of functional toxin/immunity genes linked to contact-dependent growth inhibition (CDI) and rearrangement hotspot (Rhs) systems PLoS Genet 2011 7 8 e1002217 10.1371/journal.pgen.1002217 21829394 PMC3150448

[B182] Kampstra P Beanplot: A Boxplot Alternative for Visual Comparison of Distributions J Stat Softw 2008 28 1 1 9

[B183] Diner EJ Beck CM Webb JS Low DA Hayes CS Identification of a target cell permissive factor required for contact-dependent growth inhibition (CDI) Genes Dev 2012 26 5 515 525 10.1101/gad.182345.111 22333533 PMC3305988

[B184] Iyer LM Koonin EV Aravind L Evolution of bacterial RNA polymerase: implications for large-scale bacterial phylogeny, domain accretion, and horizontal gene transfer Gene 2004 335 73 88 15194191 10.1016/j.gene.2004.03.017

[B185] Vollmer W Bacterial outer membrane evolution via sporulation? Nat Chem Biol 2012 8 1 14 18 22173345 10.1038/nchembio.748

[B186] Simeone R Bottai D Brosch R ESX/type VII secretion systems and their role in host-pathogen interaction Curr Opin Microbiol 2009 12 1 4 10 10.1016/j.mib.2008.11.003 19155186

[B187] Pallen MJ Chaudhuri RR Henderson IR Genomic analysis of secretion systems Curr Opin Microbiol 2003 6 5 519 527 10.1016/j.mib.2003.09.005 14572546

[B188] Bateman A Bycroft M The structure of a LysM domain from E. coli membrane-bound lytic murein transglycosylase D (MltD) J Mol Biol 2000 299 4 1113 1119 10.1006/jmbi.2000.3778 10843862

[B189] Finn RD Mistry J Tate J Coggill P Heger A Pollington JE Gavin OL Gunasekaran P Ceric G Forslund K The Pfam protein families database Nucleic Acids Res 2010 38 Database issue D211 222 19920124 10.1093/nar/gkp985 PMC2808889

[B190] Ponting CP Aravind L Schultz J Bork P Koonin EV Eukaryotic signalling domain homologues in archaea and bacteria Ancient ancestry and horizontal gene transfer. J Mol Biol 1999 289 4 729 745 10.1006/jmbi.1999.2827 10369758

[B191] Wren BW A family of clostridial and streptococcal ligand-binding proteins with conserved C-terminal repeat sequences Mol Microbiol 1991 5 4 797 803 10.1111/j.1365-2958.1991.tb00752.x 1830357

[B192] Dean P Functional domains and motifs of bacterial type III effector proteins and their roles in infection FEMS Microbiol Rev 2011 35 6 1100 1125 10.1111/j.1574-6976.2011.00271.x 21517912

[B193] Hayes F Van Melderen L Toxins-antitoxins: diversity, evolution and function Crit Rev Biochem Mol Biol 2011 46 5 386 408 10.3109/10409238.2011.600437 21819231

[B194] Ishikawa K Fukuda E Kobayashi I Conflicts targeting epigenetic systems and their resolution by cell death: novel concepts for methyl-specific and other restriction systems DNA Res 2010 17 6 325 342 10.1093/dnares/dsq027 21059708 PMC2993543

[B195] Iyer LM Babu MM Aravind L The HIRAN domain and recruitment of chromatin remodeling and repair activities to damaged DNA Cell Cycle 2006 5 7 775 782 10.4161/cc.5.7.2629 16627993

[B196] Iyer LM Abhiman S Aravind L MutL homologs in restriction-modification systems and the origin of eukaryotic MORC ATPases Biol Direct 2008 3 8 10.1186/1745-6150-3-8 18346280 PMC2292703

[B197] Juhas M van der Meer JR Gaillard M Harding RM Hood DW Crook DW Genomic islands: tools of bacterial horizontal gene transfer and evolution FEMS Microbiol Rev 2009 33 2 376 393 10.1111/j.1574-6976.2008.00136.x 19178566 PMC2704930

[B198] Nazina TN Tourova TP Poltaraus AB Novikova EV Grigoryan AA Ivanova AE Lysenko AM Petrunyaka VV Osipov GA Belyaev SS Taxonomic study of aerobic thermophilic bacilli: descriptions of Geobacillus subterraneus gen. nov., sp. nov. and Geobacillus uzenensis sp. nov. from petroleum reservoirs and transfer of Bacillus stearothermophilus, Bacillus thermocatenulatus, Bacillus thermoleovorans, Bacillus kaustophilus, Bacillus thermodenitrificans to Geobacillus as the new combinations G. stearothermophilus, G. th. Int J Syst Evol Microbiol 2001 51 Pt 2 433 446 11321089 10.1099/00207713-51-2-433

[B199] Viswanathan VK Eating in, eating out Gut Microbes 2010 1 4 207 208 10.4161/gmic.1.4.12321 21327026 PMC3023601

[B200] Paster BJ Boches SK Galvin JL Ericson RE Lau CN Levanos VA Sahasrabudhe A Dewhirst FE Bacterial diversity in human subgingival plaque J Bacteriol 2001 183 12 3770 3783 10.1128/JB.183.12.3770-3783.2001 11371542 PMC95255

[B201] Jain R Poulos MG Gros J Chakravarty AK Shuman S Substrate specificity and mutational analysis of Kluyveromyces lactis gamma-toxin, a eukaryal tRNA anticodon nuclease RNA 2011 17 7 1336 1343 10.1261/rna.2722711 21610213 PMC3138569

[B202] Klassen R Paluszynski JP Wemhoff S Pfeiffer A Fricke J Meinhardt F The primary target of the killer toxin from Pichia acaciae is tRNA(Gln) Mol Microbiol 2008 69 3 681 697 10.1111/j.1365-2958.2008.06319.x 18532979

[B203] Lu J Huang B Esberg A Johansson MJ Bystrom AS The Kluyveromyces lactis gamma-toxin targets tRNA anticodons RNA 2005 11 11 1648 1654 10.1261/rna.2172105 16244131 PMC1370851

[B204] Conticello SG The AID/APOBEC family of nucleic acid mutators Genome Biol 2008 9 6 229 10.1186/gb-2008-9-6-229 18598372 PMC2481415

[B205] Kanazawa T Watanabe M Matsushima-Hibiya Y Kono T Tanaka N Koyama K Sugimura T Wakabayashi K Distinct roles for the N- and C-terminal regions in the cytotoxicity of pierisin-1, a putative ADP-ribosylating toxin from cabbage butterfly, against mammalian cells Proc Natl Acad Sci USA 2001 98 5 2226 2231 10.1073/pnas.051628898 11226221 PMC30120

[B206] Orth JH Schorch B Boundy S ffrench-Constant R Kubick S Aktories K Cell-free synthesis and characterization of a novel cytotoxic pierisin-like protein from the cabbage butterfly Pieris rapae Toxicon 2011 57 2 199 207 10.1016/j.toxicon.2010.11.011 21112350

[B207] van Kooij M de Groot K van Vugt H Aten J Snoek M Genotype versus phenotype: conflicting results in mapping a lung tumor susceptibility locus to the G7c recombination interval in the mouse MHC class III region Immunogenetics 2001 53 8 656 661 10.1007/s00251-001-0381-0 11797099

[B208] Kumanovics A Lindahl KF G7c in the lung tumor susceptibility (Lts) region of the Mhc class III region encodes a von Willebrand factor type A domain protein Immunogenetics 2001 53 1 64 68 10.1007/s002510100297 11261934

[B209] Taylor M Mediannikov O Raoult D Greub G Endosymbiotic bacteria associated with nematodes, ticks and amoebae FEMS Immunol Med Microbiol 2012 64 1 21 31 10.1111/j.1574-695X.2011.00916.x 22126456

[B210] Yu C Feng W Wei Z Miyanoiri Y Wen W Zhao Y Zhang M Myosin VI undergoes cargo-mediated dimerization Cell 2009 138 3 537 548 10.1016/j.cell.2009.05.030 19665975

[B211] Zhang J Xu LG Han KJ Shu HB Identification of a ZU5 and death domain-containing inhibitor of NF-kappaB J Biol Chem 2004 279 17 17819 17825 10.1074/jbc.M310737200 14769797

[B212] Georgiades K Madoui MA Le P Robert C Raoult D Phylogenomic analysis of Odyssella thessalonicensis fortifies the common origin of Rickettsiales, Pelagibacter ubique and Reclimonas americana mitochondrion PLoS One 2011 6 9 e24857 10.1371/journal.pone.0024857 21957463 PMC3177885

[B213] Fournier GP Huang J Gogarten JP Horizontal gene transfer from extinct and extant lineages: biological innovation and the coral of life Philos Trans R Soc Lond B Biol Sci 2009 364 1527 2229 2239 10.1098/rstb.2009.0033 19571243 PMC2873001

[B214] Wolf YI Aravind L Koonin EV Rickettsiae and Chlamydiae: evidence of horizontal gene transfer and gene exchange Trends Genet 1999 15 5 173 175 10.1016/S0168-9525(99)01704-7 10322483

[B215] Iyer LM Abhiman S de Souza RF Aravind L Origin and evolution of peptide-modifying dioxygenases and identification of the wybutosine hydroxylase/hydroperoxidase Nucleic Acids Res 2010 38 16 5261 5279 10.1093/nar/gkq265 20423905 PMC2938197

[B216] Aravind L Abhiman S Iyer LM Natural history of the eukaryotic chromatin protein methylation system Prog Mol Biol Transl Sci 2011 101 105 176 21507350 10.1016/B978-0-12-387685-0.00004-4

[B217] Smith EE Sims EH Spencer DH Kaul R Olson MV Evidence for diversifying selection at the pyoverdine locus of Pseudomonas aeruginosa J Bacteriol 2005 187 6 2138 2147 10.1128/JB.187.6.2138-2147.2005 15743962 PMC1064051

[B218] Altschul SF Madden TL Schaffer AA Zhang J Zhang Z Miller W Lipman DJ Gapped BLAST and PSI-BLAST: a new generation of protein database search programs Nucleic Acids Res 1997 25 17 3389 3402 10.1093/nar/25.17.3389 9254694 PMC146917

[B219] Eddy SR A new generation of homology search tools based on probabilistic inference Genome Inform 2009 23 1 205 211 20180275

[B220] Lassmann T Frings O Sonnhammer EL Kalign2: high-performance multiple alignment of protein and nucleotide sequences allowing external features Nucleic Acids Res 2009 37 3 858 865 10.1093/nar/gkn1006 19103665 PMC2647288

[B221] Edgar RC MUSCLE: a multiple sequence alignment method with reduced time and space complexity BMC Bioinforma 2004 5 113 10.1186/1471-2105-5-113 PMC517706 15318951

[B222] Pei J Sadreyev R Grishin NV PCMA: fast and accurate multiple sequence alignment based on profile consistency Bioinformatics 2003 19 3 427 428 10.1093/bioinformatics/btg008 12584134

[B223] Cole C Barber JD Barton GJ The Jpred 3 secondary structure prediction server Nucleic Acids Res 2008 36 Web Server issue W197 201 18463136 10.1093/nar/gkn238 PMC2447793

[B224] Buchan DW Ward SM Lobley AE Nugent TC Bryson K Jones DT Protein annotation and modelling servers at University College London Nucleic Acids Res 2010 38 Web Server issue W563 568 20507913 10.1093/nar/gkq427 PMC2896093

[B225] Krogh A Larsson B von Heijne G Sonnhammer EL Predicting transmembrane protein topology with a hidden Markov model: application to complete genomes J Mol Biol 2001 305 3 567 580 10.1006/jmbi.2000.4315 11152613

[B226] Kall L Krogh A Sonnhammer EL Advantages of combined transmembrane topology and signal peptide prediction--the Phobius web server Nucleic Acids Res 2007 35 Web Server issue W429 432 17483518 10.1093/nar/gkm256 PMC1933244

[B227] Soding J Biegert A Lupas AN The HHpred interactive server for protein homology detection and structure prediction Nucleic Acids Res 2005 33 Web Server issue W244 248 15980461 10.1093/nar/gki408 PMC1160169

[B228] Holm L Kaariainen S Rosenstrom P Schenkel A Searching protein structure databases with DaliLite v. 3 Bioinformatics 2008 24 23 2780 2781 10.1093/bioinformatics/btn507 18818215 PMC2639270

[B229] Price MN Dehal PS Arkin AP FastTree 2–approximately maximum-likelihood trees for large alignments PLoS One 2010 5 3 e9490 10.1371/journal.pone.0009490 20224823 PMC2835736

[B230] Humphrey W Dalke A Schulten K VMD: visual molecular dynamics J Mol Graph 1996 14 1 33 38 10.1016/0263-7855(96)00018-5 8744570

