# Supplementary material for: Polymorphic toxin systems: Comprehensive characterization of trafficking modes, processing, mechanisms of action, immunity and ecology using comparative genomics
Source: Biol Direct. 2012 Jun 25;7:18. doi: 10.1186/1745-6150-7-18 (PMC3482391; doi:10.1186/1745-6150-7-18)
Supplement: Additional file 1 — Polymorphic toxin systems: comprehensive characterization of trafficking modes, processing, mechanisms, immunity and ecology using comparative genomics. [file 1745-6150-7-18-S1.html]

Supplementary material for the manuscript "Polymorphic toxin systems: comprehensive characterization of trafficking modes, processing, mechanisms, immunity and ecology using comparative genomics"


  
**Polymorphic toxin systems: comprehensive characterization of trafficking modes, processing, mechanisms, immunity and ecology using comparative genomics**  
  
Dapeng Zhang1, Robson F. de Souza1,2, Vivek Anantharaman1, Lakshminarayan M. Iyer1 and L. Aravind1,\*,   
\* *Address for correspondence: L. Aravind (aravind@mail.nih.gov)*  
  

*1National Center for Biotechnology Information, National Library of Medicine, National Institutes of Health, Bethesda, MD 20894, USA
  
2Departamento de Microbiologia, Instituto de Ciencias Biomedicas, Universidade de Sao Paulo, Sao Paulo, Brazil*  
  

---

|  |
| --- |
|  |
| **Abstract** Paste abstract here |

---

|  |  |
| --- | --- |
|  | **Multiple sequence alignments**  - Multiple sequence alignments of all domains described in this study - Comprehensive multiple sequence alignment and domain architectures of the PrsW family of peptidases  **Toxin Classification**  - Hierarchical classification of toxin domains based on their molecular functions  **Database-related files**  - Comprehensive list of gene neighborhoods associated with polymorphic toxins - **Tables**     - Functional classification of all domains present in toxin and toxin-related proteins - Compact representation of gene neighborhoods - Compact representation of polyimmunity loci - Comprehensive tabular representation of gene neighborhoods for toxin and immunity proteins This is the data used to generate the barplots in Figure 14 - Gene neighborhood network for toxin and immunity proteins [Cytoscape file] [PDF] - Domain architecture for all proteins analysed in this study - Diverse domain architectures of Amoebophilus asiaticus proteins containing the APD1 and APD2-like prodomains - Secretory system assignments for toxins - Life style and protein abundance per taxa: number of toxins, active toxins and immunity proteins and life style information as seen in figure 13- **Sequences**       - All toxins- All immunity proteins- **Protein identifiers (GI numbers)**         - List of all toxins (NCBI GI numbers) - List of active toxins: active toxins are toxins with N-terminal secretory system markers or that are encoded downstream to genes containing such markers - List of all immunity proteins- **Sequence analysis details**           - PSI-BLAST searches leading to the identification of novel             domains described in this study. - Seeds used for domain discovery in the above searches/analysis - Seeds for all domains discussed in this work - Domain coordinates in various toxins, immunity proteins and relevant proteins in their gene neighborhood - Tabular output of searches with all the profiles created for this work (either PSI-BLAST or HMM) against the NR database on May 23rd 2012 |
